# N-terminal domain of polypyrimidine-tract binding protein is a dynamic folding platform for adaptive RNA recognition

**DOI:** 10.1093/nar/gkae713

**Published:** 2024-08-24

**Authors:** Fred F Damberger, Miroslav Krepl, Rajika Arora, Irene Beusch, Christophe Maris, Georg Dorn, Jiří Šponer, Sapna Ravindranathan, Frédéric H-T Allain

**Affiliations:** Institute of Biochemistry, ETH Zurich, 8093 Zurich, Switzerland; Department of Biology, ETH Zurich, 8093 Zurich, Switzerland; Institute of Biophysics of the Czech Academy of Sciences, Kralovopolska 135, Brno 612 00, Czech Republic; Institute of Biochemistry, ETH Zurich, 8093 Zurich, Switzerland; Department of Biology, ETH Zurich, 8093 Zurich, Switzerland; Department of Biology, ETH Zurich, 8093 Zurich, Switzerland; Department of Biology, ETH Zurich, 8093 Zurich, Switzerland; Institute of Biophysics of the Czech Academy of Sciences, Kralovopolska 135, Brno 612 00, Czech Republic; Central NMR Facility, CSIR-National Chemical Laboratory, Pune 411008, India; Institute of Biochemistry, ETH Zurich, 8093 Zurich, Switzerland; Department of Biology, ETH Zurich, 8093 Zurich, Switzerland

## Abstract

The N-terminal RNA recognition motif domain (RRM1) of polypyrimidine tract binding protein (PTB) forms an additional C-terminal helix α3, which docks to one edge of the β-sheet upon binding to a stem-loop RNA containing a UCUUU pentaloop. Importantly, α3 does not contact the RNA. The α3 helix therefore represents an allosteric means to regulate the conformation of adjacent domains in PTB upon binding structured RNAs. Here we investigate the process of dynamic adaptation by stem-loop RNA and RRM1 using NMR and MD in order to obtain mechanistic insights on how this allostery is achieved. Relaxation data and NMR structure determination of the free protein show that α3 is partially ordered and interacts with the domain transiently. Stem-loop RNA binding quenches fast time scale dynamics and α3 becomes ordered, however microsecond dynamics at the protein-RNA interface is observed. MD shows how RRM1 binding to the stem-loop RNA is coupled to the stabilization of the C-terminal helix and helps to transduce differences in RNA loop sequence into changes in α3 length and order. IRES assays of full length PTB and a mutant with altered dynamics in the α3 region show that this dynamic allostery influences PTB function in cultured HEK293T cells.

## Graphical Abstract

**Graphical Abstract:**
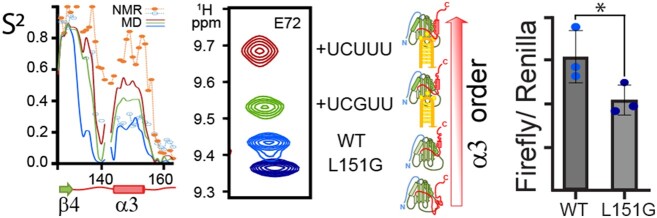

## Introduction

Specific molecular interactions are the basis for the complex organization of the cell. Recognition between molecular partners is achieved by the encounter of complementary interfaces and their mutual adaptation. Due to the need to react to changing demands from the environment these interactions are often transient and dynamic, particularly when the interacting molecules play a regulatory role. An understanding of biology at the molecular level therefore requires not only static pictures of key molecular interactions (often delivered by crystal structures and more recently cryoEM methods) but also a dynamic view of mutual adaptation between the binding partners, that sample fluctuating ensembles whose populations dictate regulatory outcomes (1). RNA is a central molecule in the regulation of cellular metabolism, acting as both translator and messenger of the genome, as well as regulator of these functions. Generally, RNA is accompanied by a range of RNA-binding proteins to form complex dynamic ribonucleoprotein assemblies. Examples are the ribosome, spliceosome, or RNA exosome, which regulate different stages of the RNA life cycle. In all these molecular machines, the RNA/protein interfaces play an essential role, and the dynamic changes accompanying binding events regulate their function (2). Some structural studies of protein-RNA interactions have shown a shape specific recognition mechanism where the binding interface on both partners is preformed and is largely rigid, resulting in high affinity binding and a high degree of specificity. More often mutual adaptation is facilitated by conformational flexibility of one or both interacting partners, and this is an important aspect of molecular recognition, which is often a hallmark of less specific interactions with moderate affinity (3). Recent studies have shown that both proteins and RNA can sample an ensemble of conformations, which may include a sub population corresponding to the bound state conformation (4). Thus, recognition occurs by conformational selection and the interaction can be further stabilized by rearrangements reminiscent of an induced fit mechanism (1). Despite the need to understand the dynamic changes in binding partners during protein-RNA complex formation, there are still relatively few studies characterizing this process for both binding partners in detail.

Polypyrimidine tract binding protein (PTB, also called PTBP or PTBP1) is a pivotal protein in RNA metabolism, involved in numerous processes such as alternative splicing, 3′ end processing, RNA transport and stability as well as internal ribosomal entry site (IRES) mediated translation (5). IRESs are complex structured RNA sequences occurring in the 5′ non-coding region of some viral and eukaryotic mRNAs, which can recruit the ribosome to initiate translation without the requirement of a CAP modification at the 5′ end. IRESs were discovered in picornaviral mRNAs but have since been shown to occur in approximately 10% of eukaryotic mRNAs where they allow translation of specific mRNAs involved in cell development, metabolic functions, and under conditions of stress (6). As its name implies, PTB prefers tracts of pyrimidines and it is able to bind a wide range of RNA targets and participate in their remodeling. PTB consists of four RNA recognition motifs (RRMs) (7) separated by linkers of variable length. The structures of the individual RRMs (or tandem RRMs) of PTB bound to short single-stranded CU-rich RNA have been reported previously and show that each RRM binds 3 to 5 single-stranded nucleotides on the exposed β-sheet surface (8).

Evidence has accumulated that PTB can act as an RNA chaperone, stabilizing a particular three-dimensional arrangement of the IRES secondary structure elements required for binding eukaryotic initiation factors which then recruit the ribosome and initiate translation (9–11). In both its roles in alternative splicing and IRES-mediated translation, PTB has been proposed to use its RRM modules as adaptors to grasp the RNA substrate and stabilize particular conformations. Recently, integrative structural biology has shown that PTB binding restricts the conformational space of adjacent IRES binding domains while maintaining considerable RNA flexibility (11). However, it is still unclear how these PTB-mediated RNA conformational changes are controlled.

We previously reported the structure of the N-terminal RRM of PTB including 20- and 30- residue N- and C-terminal flanking sequences (PTB RRM1) bound to a stem-loop RNA containing the pentaloop sequence UCUUU (12), which is frequently bound by PTB in viral IRES. We showed that the region just C-terminal to RRM1 undergoes a conformational change upon binding with the formation of an additional helix, α3, which interacts with one edge (strand β2) of the β-sheet RNA-binding platform. Importantly, α3 showed no contacts to the RNA. Furthermore, RNA stem-loops containing variant loop sequences substituting G for U at various positions in the UCUUU pentaloop showed nearly the same affinity, but significantly lower α-helical content for α3 as indicated by the analysis of protein chemical shift changes. We concluded that the downstream region of RRM1 acts as a sensor of RNA secondary structure and can transmit the binding of a stem–loop by RRM1 to RRM2, as the interdomain linker will change its length depending on which sequence is bound. Formation of α3 can therefore also influence the relative distance of the RNA secondary structure elements, which are bound by the two RRMs, representing a mechanism for PTB-mediated control of the global RNA conformation. In cell translation assays and *in vitro* gel shift assays with a PTB α3 deletion mutant supported the importance of the conserved α3 region for PTBs ability to enhance IRES-mediated translation and provided evidence for changes in the IRES–RNA complex influenced by α3. This is in line with work showing that PTB helps IRES to attain conformations similar to those needed to recruit the ribosome (13). Although this work shed light on the importance of the α3 region in controlling PTB function, it was unclear how these conformational changes are achieved, since the SL RNA made no direct contacts with the α3 helix in the PTB RRM1 complex. In, addition, the mechanistic basis for the dynamic regulation of α3 helix order by binding of SL RNAs with different loop sequences remained to be elucidated.

In order to establish the dynamic origin of this allosteric regulation and elucidate its mechanism, we have performed fast and slow timescale dynamics studies of PTB RRM1 and SL UCUUU in the free and bound states by NMR spectroscopy, and determined the structure of free PTB RRM1. We show that a partially structured α3 is already present in the dynamic ensemble of the free state with evidence that part of the ensemble includes contacts between α3 and the RRM domain similar to those in the complex. We then performed microsecond standard and enhanced sampling molecular dynamics simulations of the free protein, as well as complexes with SL RNAs to show how a network of solvent bridging interactions couple stem-loop binding to α3 helix folding, accounting for the influence of the RNA loop sequence on the α3 helix. Finally, IRES mediated translation assays with a full length PTB, incorporating a mutation which shifts the conformational equilibrium of α3 to a less ordered state, establishes the importance of this dynamic transition for the control of PTB function in cells. Together our data demonstrate that α3 helix formation in PTB is an example of dynamic allostery, a general means of coupling disorder/order transitions to functional outcomes. More globally, our comprehensive dynamics study shows that the entire C-terminal half of the RRM is highly dynamic, indicating that the RRM is more than an RNA binding module, but a dynamically regulated entity which can respond to different effectors by transmitting information across the RRM interface or via the RRM interdomain linkers.

## Materials and methods

### NMR sample preparation

The NMR samples employed in this study were prepared as described previously (12). Briefly, PTB RRM1 and the variant PTB RRM1[L151G], were recombinantly expressed in *E. coli* in minimal media supplemented with ^15^NH_4_Cl and ^13^C_6_-glucose as an intein-tagged fusion protein. After intein digestion and purification, the isolated protein contained only the native (or L151G mutant) protein sequence (human PTBP1, accession number X62006, residues 41–163). RNA was prepared using run-off transcription with either standard NTPs or uniformly ^13^C,^15^N-labeled NTPs prepared in-house, and purified by denaturing anion-exchange chromatography and snap-cooled in dilute conditions to ensure hairpin formation. NMR samples range from 0.5–1.0 mM in protein and RNA concentration. NMR buffer consisted of 20mM NaCl, 10 mM NaH_2_PO_4_ adjusted to pH 6.5 with NaOH and included 10% D_2_O.

### NMR spectroscopy

NMR experiments were performed on Bruker 500, 700, 750 and 900 MHz Avance spectrometers equipped with triple resonance z-axis gradient probes and triple resonance z-axis gradient cryo-probes. ^15^N R_1_, R_2_ and NOE experiments were carried out on a 700 MHz spectrometer at 313K employing published pulse sequences (14–16). The R_1_ experiment applied periodic selective inversion of the amide ^1^H region during the T_1_ relaxation period to suppress the influence of ^15^N CSA/^1^H-^15^N dipolar cross-correlated relaxation. R_1_ relaxation was sampled with delays ranging from 40–1520 ms for PTB RRM1 and 80–1800 ms for the PTB RRM1-SL UCUUU complex with two repeat measurements each. R_2_ was estimated from on-resonance R_1ρ_ measurements based on the relation *R*_1ρ_ = *R*_1_ cos^2^θ + *R*_2_ sin^2^θ, where θ = arctan(ω_SL_/ω_off_), and ω_SL_ and ω_off_ are the field strength and irradiation offset of the spin-lock field respectively. The highest possible spin-lock field strength (2.4 kHz) was employed to ensure that the on-resonance or near-resonance condition was satisfied across the entire ^15^N spectral range, thereby avoiding offset related issues, which are observed in CPMG based R_2_ measurements employing a single CPMG carrier position at the center of the ^15^N spectral range (17,18). The relaxation rate measurements employed 8 delays of 8–120 ms for PTB RRM1 and 8–96 ms for PTB RRM1-SL UCUUU complex with two repeat measurements each. All measurements were carried out in an interleaved manner with the relaxation delay incremented after each scan, alternating long and short relaxation delays. For heteronuclear NOE experiments, ^1^H irradiation was applied for the last 3 s of a 5 s recycle delay and was omitted in the reference experiment.

The relaxation compensated I_z_S_z_ (RCZZ) experiment (19,20) was carried out on a 750 MHz spectrometer at 298K, for mapping out slow exchange events in PTB RRM1 and its L151G mutant. Two spectra were recorded in an interleaved manner, in which the relaxation experiment included an additional delay 2τ = *n*/*J*_NH_ = 129.6 ms whereas the reference experiment omitted this delay. The ratio of intensities from the two experiments, *I* and *I*_ref_ gives the relaxation rate *R*_2_^RCZZ^ = -(1/2τ_he_)ln(*I*/*I*_ref_). The slow exchange contribution to relaxation was calculated using the relation *R*_ex_ = *R*_2_^RCZZ^ – κη_xy_ + *R*_1_/2, where *R*_1_ is the longitudinal relaxation rate, η_xy_ is the ^15^N CSA/^1^H-^15^N dipolar cross correlated transverse relaxation rate, and κη_xy_ represents the intrinsic transverse relaxation rate due to ^15^N CSA and ^1^H-^15^N dipolar interactions alone, which was calculated assuming (21,22)


(1) \begin{eqnarray*}{\mathrm{\kappa \ = \ }} - \frac{{6\left[ {\left( {{{d}^2}/8} \right) + \left( {{{c}^2}/6} \right)} \right]}}{{\sqrt 3 cd\,{{P}_2}( {\cos \beta })}}\end{eqnarray*}

The CSA and dipolar interaction constants are given by *c* = ω_N_(σ_$\|$_-σ_$\perp$_)/3^1/2^ and *d* = (μ_0_*h*γ_H_γ_N_)/8π^2^*r*^3^_NH_, respectively, where β defines the orientation of the CSA component σ_$\|$_ with respect to the NH bond vector. Assuming an N–H interatomic distance of 1.02 Å, an average ^15^N CSA magnitude of −164 ppm, and that the major axis of the ^15^N CSA makes an angle of 18° with respect to the N-H dipolar interaction (23), κ is calculated to be 1.2283 at 750 MHz. Cross-correlated relaxation rates were measured with standard pulse sequences (24,25). Two sets of measurements were carried out with relaxation delays *T* of 16, 32, 40 ms, in which experiment ‘A’ measures signals proportional to the initial coherence *N_x_*, and experiment ‘B’ measures signals proportional to the coherence 2N_*x*_H_*z*_, which results from CSA/dipole cross correlation. The cross correlated transverse relaxation rate is obtained from a fit of the intensity ratios to the expression *I*_B_/*I*_A_ = tanh(η_xy_*T*). The *R*_1_ measurements were carried out with 8 delays of 80–1200 ms with two repeat measurements.

^15^N CPMG relaxation dispersion measurements were carried out for PTB RRM1 and its L151G mutant on 750 and 900MHz spectrometers at 298 K using published sequences (26–28). The experiments were measured in a pseudo 3D manner with CPMG relaxation occurring for a constant time of 80 ms at 750 MHz and 64ms at 900 MHz. The experiments on the 750MHz spectrometer were obtained for ^15^N 180° pulse repetition frequencies (υ_CP_) of 25–750 Hz, whereas on the 900 MHz spectrometer CPMG frequencies of 31.25–750 Hz were employed. Relaxation rates were calculated as *R*_2_^eff^ = −(1/*T*)ln(*I*_CPMG_/*I*_ref_). The measurements were made at two CPMG irradiation offsets of 116 ppm and 125 ppm. For wild type PTB RRM1, the CPMG relaxation dispersion measurements were also performed at 313K with CPMG offsets of 116, 120 and 124 ppm on both the 750 MHz and 900 MHz spectrometers. Different offsets were chosen to ensure that small dispersion effects are visible, which would otherwise be obscured by large offset related distortions of relaxation rates, which are known to occur in CPMG based methods (29).

^15^N **R**_1ρ_ relaxation dispersion experiments were carried out for PTB RRM1 bound to SL UCUUU on a 750 MHz spectrometer at 313 K, employing off-resonance and on-resonance spin-lock irradiation, using published sequences (30,31). In off-resonance experiments, R_1ρ_ was measured as a function of the effective field ω^2^_eff_ = ω^2^_SL_ + ω^2^_off_, where ω_SL_ is the spin-lock field strength, and ω_off_ is the spin-lock irradiation offset. The spin-lock field strength was set to 1400 Hz for 10 irradiation frequencies of 79–168 ppm. The irradiation offsets correspond to tilt angles in the range 90−19.5 degrees. Alignment of the magnetization with the effective field was obtained by using adiabatic pulses with atanh/tan amplitude/frequency modulation function (30). Relaxation delays ranged from 8–180 ms for the *R*_1ρ_ measurements, depending on the ω_eff_ values. On-resonance *R*_1ρ_ measurements were performed for spin-lock field strengths of 800, 900 and 1100 Hz. In this case, only resonances with ω_off_/ω_SL_ ≤ 0.4 were analyzed to minimize alignment errors (31).

^13^C *R*_1ρ_ relaxation dispersion experiments for uniformly ^13^C-labeled SL UCUUU in the free and PTB RRM1 bound states were carried out on a 500 MHz spectrometer at 313 K. Standard pulse sequences, implementing an INEPT transfer, variable relaxation period, ^13^C frequency labeling, and final INEPT transfer for proton detection, were employed for the relaxation rate measurements (32). In the case of C1′ and C6 carbons, which have ^13^C neighbors, shaped pulses (IBURP) were used for selective excitation of the desired carbon resonances, and ^13^C frequency labeling was implemented in a constant time manner (33,34). *R*_1ρ_ was measured as a function of effective field, keeping the irradiation offset constant and varying the spin-lock field strength in the range 700–6000 Hz. The carrier was positioned so that ω_off_/ω_SL_ ≤ 0.4, ensuring the magnetization is aligned along the effective field. Relaxation delays ranged from 10–600 ms for R_1_ experiments and 2–34 ms for *R*_1ρ_ experiments. The *R*_2_ rate was calculated from the measured *R*_1ρ_ and *R*_1_ rates as described above for ^15^N measurements.

The C1′ carbons, which showed *R*_1ρ_ dispersion in the 2D experiments, were sufficiently resolved to allow more extensive *R*_1ρ_ measurements to be carried out by 1D experiments employing selective Hartmann−Hahn polarization transfers, exciting only the resonance of interest (35,36). On-resonance *R*_1ρ_ dispersion experiments were performed for spin-lock field strengths ranging from 100–4000 Hz, while off-resonance *R*_1ρ_ dispersion experiments were carried out at a fixed spin-lock field strength of 200 Hz with offsets ranging from −1500 to +1500 Hz.

### NMR data processing and analysis

Data were processed using Bruker Topspin software, and peak intensities were extracted using Sparky (www.cgl.ucsf.edu/home/sparky/) or CARA (www.nmr.ch) (37,38). MOLMOL was used to analyze and generate images of structures (39). Analysis of the relaxation data based on reduced spectral density mapping (16,40) is described in Supplementary note S1. Analysis of the relaxation data based on the extended model-free approach are well documented, and were carried out as described previously (41,42).

The CPMG relaxation dispersion data were analyzed in terms of the two-site general exchange equation. The effective relaxation rate is given by (28,43),


(2a) \begin{eqnarray*}R_2^{eff}\left( {{{\upsilon }_{CP}}} \right) &=& \frac{1}{2}\left( {{R}_a} + {{R}_b} + {{k}_{ex}} - 2{{\upsilon }_{CP}} \right. \nonumber\\ && \left. \times \, \left[ {{D}_ + }cosh\left( {{{\eta }_ + }} \right) - {{D}_ - }cos\left( {{{\eta }_ - }} \right) \right] \right)\end{eqnarray*}

(2b) \begin{eqnarray*}{{D}_ \pm } = \frac{1}{2}\left[ { \pm 1 + \frac{{\psi + 2{\mathrm{\Delta }}{{\omega }^2}}}{{{{{\left( {{{\psi }^2} + {{\xi }^2}} \right)}}^{1/2}}}}} \right]\end{eqnarray*}

(2c) \begin{eqnarray*}{{\eta }_ \pm } = \frac{1}{{\sqrt 8 {{\nu }_{CP}}}}{{\left[ { \pm \psi + {{{\left( {{{\psi }^2} + {{\xi }^2}} \right)}}^{1/2}}} \right]}^{1/2}}\end{eqnarray*}

(2d) \begin{eqnarray*}\psi = {{\left[ {{{R}_a} + {{R}_b} + \left( {{{p}_b} - {{p}_a}} \right){{k}_{ex}}} \right]}^2} - {\mathrm{\Delta }}{{\omega }^2} + 4{{p}_a}{{p}_b}k_{ex}^2\end{eqnarray*}

(2e) \begin{eqnarray*}\xi = 2{\mathrm{\Delta }}\omega \left[ {{{R}_a} - {{R}_b} + \left( {{{p}_b} - {{p}_a}} \right){{k}_{ex}}} \right]\end{eqnarray*}

where *p*_a_ and *p*_b_ are the fractional populations of the two sites, which have a chemical shift difference of Δω, *k*_ex_, is the rate of exchange between the two sites, and *R*_a_ and *R*_b_ are the intrinsic relaxation rates of the two sites. In calculations, the intrinsic relaxation rates at two static magnetic fields are independently fitted parameters, however at a given field, they were assumed to be the same for the two exchanging sites (*R*_a_ = *R*_b_).

For *R*_1ρ_ dispersion experiments, *R*_2_^eff^ estimated from *R*_1ρ_ and *R*_1_ rates (see above) were analyzed in terms of the two-site fast exchange model (43,44)


(3) \begin{eqnarray*}R_2^{eff} = \left( {R_2^0} \right) + \frac{{{{p}_a}{{p}_b}{\mathrm{\Delta }}{{\omega }^2}{{k}_{ex}}}}{{\omega _{SL}^2 + \omega _{off}^2 + k_{ex}^2}}\end{eqnarray*}

where *R*_2_^0^ refers to the in-phase transverse relaxation rate in the absence of exchange and was fixed to κη_xy_ as discussed above for the RCZZ experiments (Eq. 1), ω_SL_ and ω_off_ are the spin-lock field strength and spin-lock irradiation offset respectively, and the fitted parameters are ϕ^SL^_ex_ = *p*_a_*p*_b_Δω^2^ and *k*_ex_.

For ^13^C relaxation dispersion experiments, the *R*_1ρ_ dispersion profiles were fit directly by fixing R_1_ to the values determined in longitudinal relaxation rate experiments and optimizing *R*_2_^0^, ϕ^SL^_ex_ = *p*_a_*p*_b_Δω^2^ and *k*_ex_. All calculations were carried out using in-house software and gnuplot scripts.

### Structure determination of free PTB RRM1

Complete resonance assignments of PTB RRM1 at 298 K were obtained using triple resonance experiments for backbone assignment, and both HCC(CO)NH and HCCH-type TOCSY experiments for the sidechains. Assignment was also supported by an analysis of 3D ^15^N- and ^13^C-resolved NOESY spectra (45). Both a ^15^N- and a ^13^C-resolved NOESY spectrum of the free protein were measured on an Avance III 900 spectrometer, using a mixing time of 60 ms, and either GARP4 for ^15^N decoupling, or BUSS (46) for ^13^C decoupling of both aliphatic and aromatic resonances, respectively. Resonance assignment was performed manually using CARA (38). Peaklists were obtained from NOESY spectra using the CYPICK peak-picking algorithm (47) followed by manual curation to remove picked artifacts. Assignments derived from CARA, and cleaned peaklists obtained with CYPICK, were provided to the NOEASSIGN algorithm in CYANA, and the CONSENSUS protocol was used for structure calculation (48). This protocol performs 20 independent structure calculations and combines the best conformer from each calculation into a ‘combined’ ensemble. A final calculation is then performed which combines the NOE-derived distance constraints occurring in a large fraction of the individual structure calculations into ambiguous distance constraints, to generate a ‘consensus’ ensemble of 20 structures consistent with the individual calculations. Because this ensemble represents a protein containing a region with dynamic disorder, and there is no established protocol for structure refinement using ambiguous distance restraints, we did not attempt to perform structure refinement using a realistic potential (e.g. AMBER).

### Calculation of correlation times for free and bound states

We estimated the correlation times of free and bound protein using the HullRad method (49), which was developed to rapidly calculate hydrodynamic properties of proteins and nucleic acids from their structure coordinates. HullRad determines the minimal convex volume including all coordinates and estimates hydrodynamic properties using a prolate ellipsoid with an equivalent volume. This volume is expanded by an empirically determined amount to account for hydration. HullRad calculates τ_c_ values at 20°C. We multiplied these values by the ratio of water viscosity at 313 K and 293 K: 0.653 mPa s/1.002 mPa s (50) to determine τ_c_ at 313 K. Including the flexible protein termini gave very large τ_c_ values due to their extension of the structure. We therefore calculated τ_c_ after removing atoms from the flexible N- and C-terminal regions (included residues 54–154 of the protein, and all of the SL RNA). Average values and standard deviation were calculated using all members of the NMR ensemble for free protein (PDB: 8BGF), and the complex (PDB: 2N3O).

### Molecular dynamics (MD) simulations

Simulations of PTB RRM1 in complex with SL UCUUU were started from the coordinates of the first conformer of its NMR structure (PDB: 2N3O). Simulations of the free RNA were started from the RNA coordinates of the first conformer of the NMR structure of the complex (12). Starting coordinates for the simulations of the free protein were obtained either by removal of the RNA from the first conformer of the complex structure, or by utilizing one of the conformers from NMR structural ensemble of the free protein (this work). The coordinates of the PTB RRM1 L151G mutant and PTB RRM1 bound to SL UCGUU were obtained by molecular modeling of the first conformer of the wildtype structure of PTB RRM1 bound to SL UCUUU (PDB: 2N3O). We have used ff12SB (51) and bsc0χ_OL3_ (52) force fields to describe protein and RNA, respectively. All simulations were run using the AMBER 16 program package (53). In all simulations, the biomolecules were immersed in octahedral boxes of SPC/E water molecules along with 0.15 M of KCl ions (54). The minimal distance between the solute and the box border was 12 Å. Specific simulation settings and the protocol for standard MD simulations were applied as previously described (55). In addition to the standard MD simulations, we have also run REST2 (Replica-Exchange Solute Tempering 2) enhanced sampling simulations (56) to obtain better sampling of the part of the protein chain which forms the α3 helix. REST2 belongs to the brute-force enhanced sampling methods, and is based on simulated annealing coupled with a replica-exchange simulation protocol (57). In REST2, several different copies of the system are simulated in a ladder of replicas where the interaction forces in an area of interest (i.e. the ‘hot region’) are progressively scaled down as the ladder is climbed to allow more frequent energy barrier crossings. Exchange of the simulation coordinates between adjacent rungs of the ladder allows each of them to access a wider conformational space. Assuming that an overall convergence is achieved, the reference (unbiased) replica collects equilibrium populations of the conformations and thus identifies the global minimum on the free-energy landscape as defined by the force field (57). In our REST2 simulations we included residues 140−156 (corresponding to the α3 helix and its flanking residues) in the hot zone in order to focus on conformational changes in the α3 region. Six replicas were used where the lowest and highest replica corresponded to a scaling of the hot zone interactions by λ=1 and λ=0.6, respectively, and the simulations were run for 10 μs. Using these settings, an average exchange ratio of 21% was achieved between simulation replicas. The REST2 simulations were performed at constant volume conditions and a Langevin thermostat was used to regulate temperature. Other settings were the same as in the standard MD simulations. All simulations were analyzed using the VMD (58) and cpptraj (59) programs. Order parameters for MD trajectories were calculated as described previously (60).

### Plasmids

The dicistronic reporter plasmid pRemcvF and expression plasmid for WT PTBP1 pCDNA3.1-hPTBP1 are described in (12). Plasmid encoding for the mutant protein pCDNA3.1-mutPTBP1-L151G was generated using the following primer pairs:

L151G-fwd 5′GGCGGCCGGCCAGGCGGTGAACTCGGTCC3’,

L151G-Rev5’ GCCTGGCCGGCCGCCTGGGCCCGCGC3’, and pCDNA3.1-hPTBP1 as template by PCR mutagenesis.

### Luciferase assay for IRES activity

50000 Hek293T cells were plated in 12-well plates the day before transfection. For each tested condition, cells were plated in triplicate. The next day cells were transfected with 7.5nM and 10nM siPools (siTOOLs Biotech) targeting the 3′UTR region of PTBP1 and nPTB respectively or non-targeting siCntrl RNAs using Lipofectamine RNAiMAX (Invitrogen) following supplier guidelines. Twenty-four hours after transfection with siRNAs, cells were transfected with a second round of siRNAs at the same concentration as above along with 250ng of the reporter construct pRemcvF and either 250 ng of empty vector control plasmid pCDNA3.1, or plasmids expressing WT PTB pCDNA3.1-hPTBP1, or expressing mutant PTB protein pCDNA3.1-mutPTBP1-L151G, using Lipofectamine 2000 (Invitrogen) following supplier guidelines. Twenty-four hours after the second transfection, cells were processed for luciferase assay using the Dual‐Glow Luciferase Assay kit (E2920 Promega) following supplier guidelines. Half of the lysed cells from the luciferase assay were used for western blot analysis to confirm knockdown of endogenous protein and ectopic expression of the various PTB proteins. A CLARIOstar^®^PLUS (BMG Labtech) plate reader was used to detect renilla and firefly luminescence. Results are means and error bars are standard deviation (SD) from three independent experiments.

### Western blot analysis

10–20μg total cellular proteins extracted from the transfected Hek293T cells were subjected to SDS-PAGE electrophoresis and transferred to PVDF membranes for Western blot analysis. The following antibodies were used: mouse anti‐Tubulin antibody (1:10000, A01410, GenScript), rabbit anti PTB-NT (1:3000) (12), rabbit anti nPTB (1:3000,55186–1-AP, Proteintech), mouse anti M2FlagHRP (1:5000, SLBD9930 Merck). Membranes were developed using the SuperSignal™ West Femto Maximum Sensitivity Substrate (Thermo Scientific) and imaged using an Amersham ImageQuant^TM^ 800 imaging system. All membranes were stained with Coomasie to control for equal loading of proteins.

## Results

**Figure 1.**
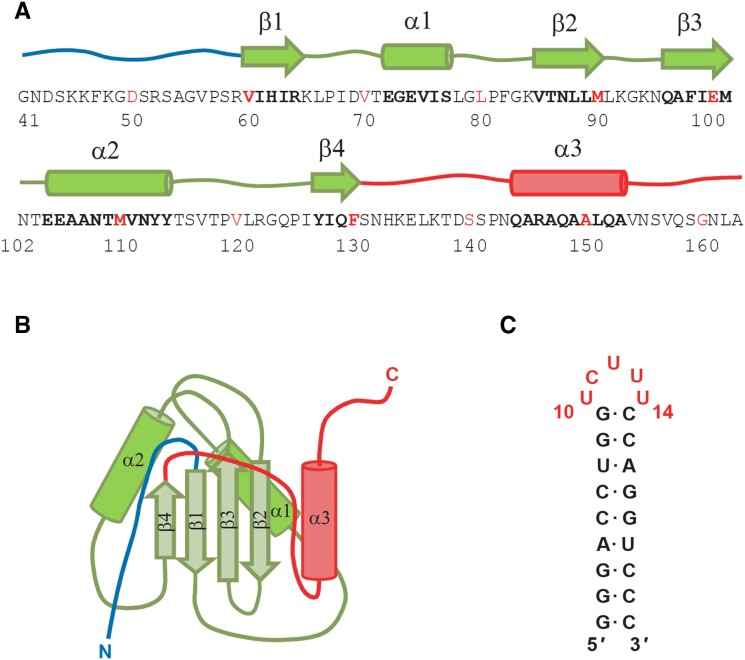
Schematic representation of (**A**) sequence and secondary structure of PTB RRM1, (**B**) structure of PTB RRM1 when bound to SL UCUUU and (**C**) sequence of SL UCUUU RNA.

The 123 amino acid protein construct employed for dynamics studies and the NMR structure determination shown in Figure 1A, B (PTB RRM1), includes the RRM1 domain of PTB along with the flanking 20 and 30 residues from the N and C termini respectively. Part of the long linker following β4 forms the α3 helix, which contacts β2 and α1, when the protein is bound to a stem-loop RNA (12). The stem-loop RNA is a 23-nucleotide hairpin, with a stem of nine Watson–Crick base pairs capped by a UCUUU pentaloop (Figure 1C). Dynamics changes upon complex formation were investigated quantitatively by a suite of ^15^N relaxation experiments for the protein and ^13^C relaxation experiments for the RNA and by molecular dynamics (MD) simulations.

### Extensive fast time scale dynamics of unbound PTB RRM1

**Figure 2.**
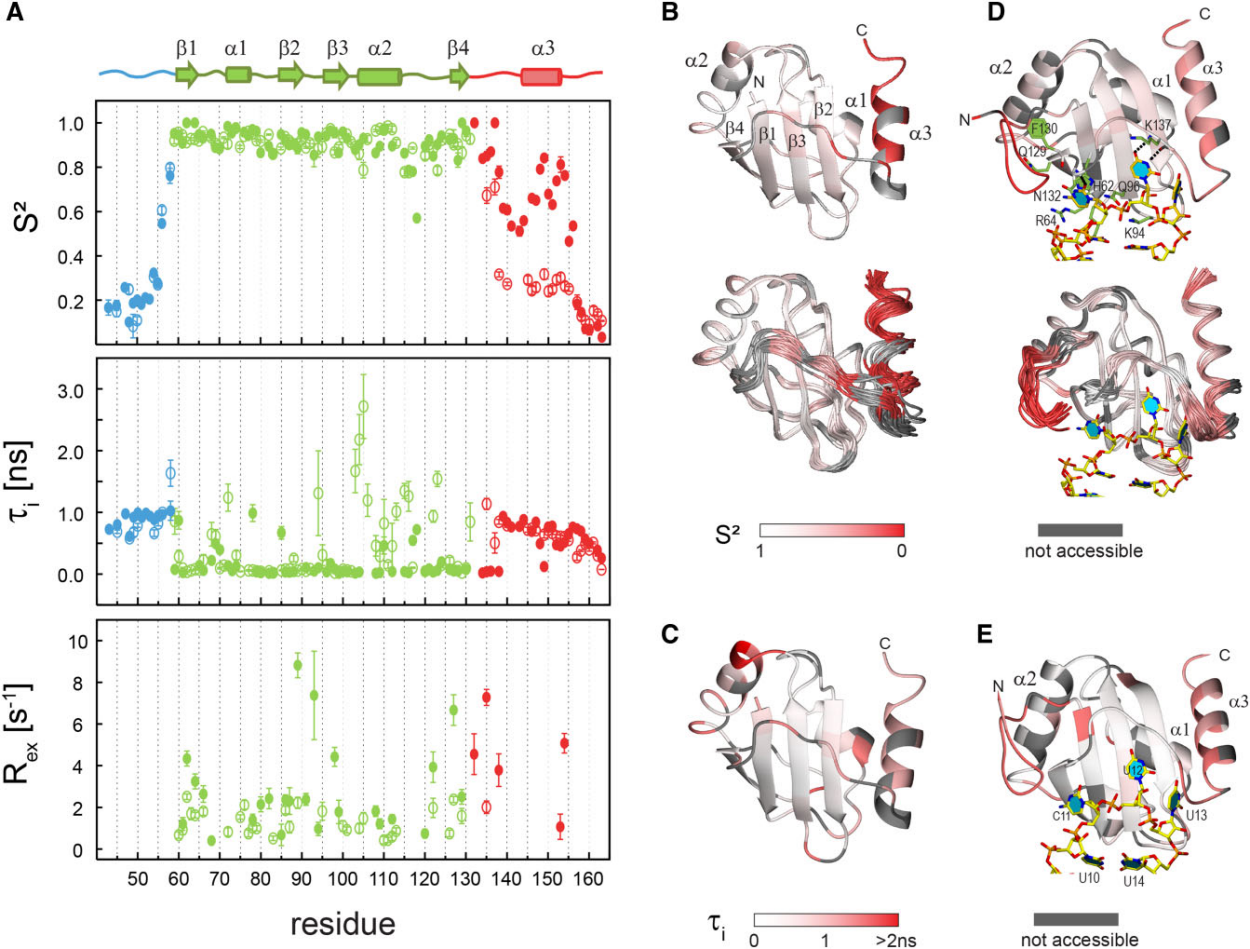
Model-free analysis of relaxation data for PTB RRM1 and PTB RRM1 bound to SL UCUUU RNA obtained at 313K. (**A**) Order parameter S^2^, internal correlation time τ_i_, and *R*_ex_ are plotted versus the residue number for free (open circles) and bound (filled circles) protein. Residues from the folded domain, the regions N-terminal and C-terminal to the domain are shown in green, blue and red, respectively. (B–E) Projection of dynamics parameters onto the structure of RRM1, where the backbone is shown by a color-coded ribbon, with red representing enhanced fast timescale mobility. (**B**) *S*^2^, and (**C**) τ_i_ of PTB RRM1, (**D**) *S*^2^, and (**E**) τ_i_ of PTB RRM1-SL UCUUU. In the lower panels of (B) and (D), *S*^2^ data are also projected on the ensemble of conformers for the free protein (PDB: 8BGF) and bound protein (PDB: 2N3O), respectively. Specific contacts between RNA and protein residues are shown in (D).

To obtain an initial characterization of dynamics changes of PTB RRM1 upon binding the stem-loop RNA, ^15^N relaxation rates (R_1_, R_2_, {^1^H}^15^N-NOE) were measured for PTB RRM1 in the free and SL UCUUU bound states (Supplementary Figure S1). The global correlation time of rotational diffusion was estimated for PTB RRM1 in the free and bound states using *R*_2_/*R*_1_ ratios of residues not subjected to slow conformational fluctuations and/or significant internal motions (41). PTB RRM1 tumbles isotropically with a correlation time of 6.54 ± 0.03 ns, whereas for PTB RRM1-SL UCUUU, an axially symmetric diffusion tensor with *D*_$\|$_/*D*_$\perp$_ ratio of 1.26 ± 0.08 between the major and minor axes, and a global correlation time of 11.79 ± 0.06 ns, were obtained. These correlation times are close to the estimates obtained from reduced spectral density mapping (RSDM) analysis (Supplementary Note S1, Supplementary Figure S2). Estimates of the correlation times using the HullRad method to calculate hydrodynamic properties based on the coordinates of the free and bound states excluding the flexible N- and C-termini of the protein (residues 54−154) give 4.15 ± 0.15 ns and 10.02 ± 0.25 ns for the free and bound states at 313 K, respectively. The larger experimental τ_c_ values, particularly for the free protein may be caused by a somewhat larger hydrodynamic radius due to the flexible regions. The ^15^N relaxation data of PTB RRM1 in the free and SL UCUUU bound states were analyzed by the model-free formalism to extract parameters describing the amplitudes and time scales of the amide NH vector reorientation. The model-free dynamics parameters for PTB RRM1 (Figure 2 and Supplementary Table S1) show a complex behavior in the free state with different dynamics characteristics for different regions. As expected, the order parameter S^2^ describing restriction of motional freedom is significantly lower for the unstructured N- and C-termini compared to the folded part of the domain, which has values close to 1. The *S*^2^ values for residues 145–155, which are C-terminal to the RRM fold, indicate that this segment has a uniform degree of restricted mobility, contrasting with the progressive decrease normally observed in a fully unstructured tail. This is also reflected in the clustering observed for data from these residues in the plot of spectral densities *J*(0) versus *J*(ω_N_), derived from RSDM (Supplementary Figure S2A) (61). This analysis suggests partial ordering of this segment in the free state. The low level of helical secondary structure indicated by ^13^C^α^ and ^13^C^β^ chemical shifts (12), suggests that this local order is due to a transient helical structure, which becomes fully folded upon RNA binding.

The correlation time for fast local motions τ_i_, shows extensive variations along the sequence in the free state (Figures 2A and C). Correlation times of hundreds of ps are consistently observed for all the N- and C-terminal residues. The relaxation rates for these residues are best described by the extended model-free spectral density function, which is typical for disordered regions with considerable amplitude for NH vector fluctuations (lower S^2^ values; Supplementary Table S1) (62). Within the structured part of the domain, most residues have τ_i_ in the 20–250 ps range, values, which are usually observed for restricted motions of residues involved in secondary structure within the folded part of the protein. A striking exception to this behavior is shown by Lys94 in the β2−β3 loop and most residues of helix α2 (105−115) along with its flanking loop residues. Even though the S^2^ values are high, the internal motions are at an intermediate time scale of 1−2ns. These unusually high τ_i_ values at the ends of helix α2 and its flanking loops could indicate collective motions or end fraying of the α2 helix or interactions with the disordered N-terminal tail. Nanosecond time scale internal motions for residues with *S*^2^> 0.85 have also been observed in regions subject to millisecond exchange events in GTP binding proteins (63). The residues 115−163 of PTB RRM1, following helix α2 are mostly comprised of long stretches of unstructured loops interrupted by the short β4 strand (residues 126−129) and some partial ordering in the 145−155 segment. The dynamics in this part of PTB RRM1 resembles those of partially folded proteins where a lack of extensive tertiary interactions can result in complex internal motions at an intermediate time scale between 250 ps (typical upper bound of internal correlation time in well folded globular proteins) and the overall correlation time (6.5 ns) (64). Several residues across PTB RRM1 also show exchange contribution, *R*_ex_, to the transverse relaxation rate (Figure 2A), consistent with their location at the extreme right in the RSDM plot (Supplementary Figure S2A). This indicates the presence of μs−ms time-scale exchange processes. Some of these residues are located within or near the β-strands, which form the RNA binding surface whereas others are in α1 and in the loops flanking α2, which are distal to the RNA binding surface. Several of these residues in PTB RRM1 exhibit CPMG dispersion curves, confirming extensive slow time scale dynamics (see below).

### Fast time scale dynamics of PTB RRM1 in the RNA bound state

Major changes in fast time scale dynamics occur in PTB RRM1 upon binding to SL UCUUU (Figure 2A, D, E, Supplementary Table S2). The *S*^2^ values increase in the C-terminal region and for residues 135−155, they are closer to values observed in the folded part of the domain. Binding to SL RNA enhances the backbone order of residues 145−155 from the α3 helix although they make no contacts to the RNA. Most of the residues at the RNA binding interface also show increased S^2^ compared to the free state. Fast time scale internal motions are quenched (S^2^= 1) for the amides of His62, Arg64, Gln129, Asn132 and Lys137. Most of these residues are involved in direct interactions with the RNA (Figure 2D, Supplementary Table S2) (12). His62 makes stacking interactions with the RNA loop nucleotide C11 while the backbone amide of Asn132 is hydrogen bonded to O2 of C11. Lys137 main chain makes hydrogen bonds to the Watson-Crick face of U12 while the sidechains of Arg64 interacts with phosphate oxygen of G9. All residues of the β2−β3 loop show increased S^2^ on RNA binding including Lys94 and Gln96, which both contact the mismatched UU base pair at the top of the RNA stem. These interactions rigidify the β2−β3 loop compared to the free state of the protein.

In contrast to the free state, in the complex most of the domain residues, with the exception of a few in the vicinity of loops, have τ_i_ values in the 20−250 ps range associated with small amplitude fluctuations of the NH vectors characteristic of well-structured regions (65). Excepting Met110, Val117 and Thr118, the residues of the α2 helix and flanking loops have τ_i_ values < 250ps whereas in the free state, the majority had τ_i_ close to 1 ns, indicating a major change in dynamical characteristics to a behavior more typical of rigid folded secondary structure. This change may be related to the positioning of the N-terminal region preceding β1 caused by interactions of R52 with the RNA backbone, or additional stabilization of the structured α2−β4 loop, which contacts α1. A similar effect on internal motion is also observed for residue Lys94, which is involved in a stacking interaction with the U10−U14 base pair. Whereas fast timescale motion is quenched for much of the protein, upon RNA binding, μs−ms time-scale motions are preserved in the RNA-bound state, with significant increases in *R*_ex_ for a number of residues, particularly in the β-sheet and connecting loops, as well as in the C-terminal extension (Figure 2A, Supplementary Table S2).

### PTB RRM1 shows extensive slow motions in the free state

**Figure 3.**
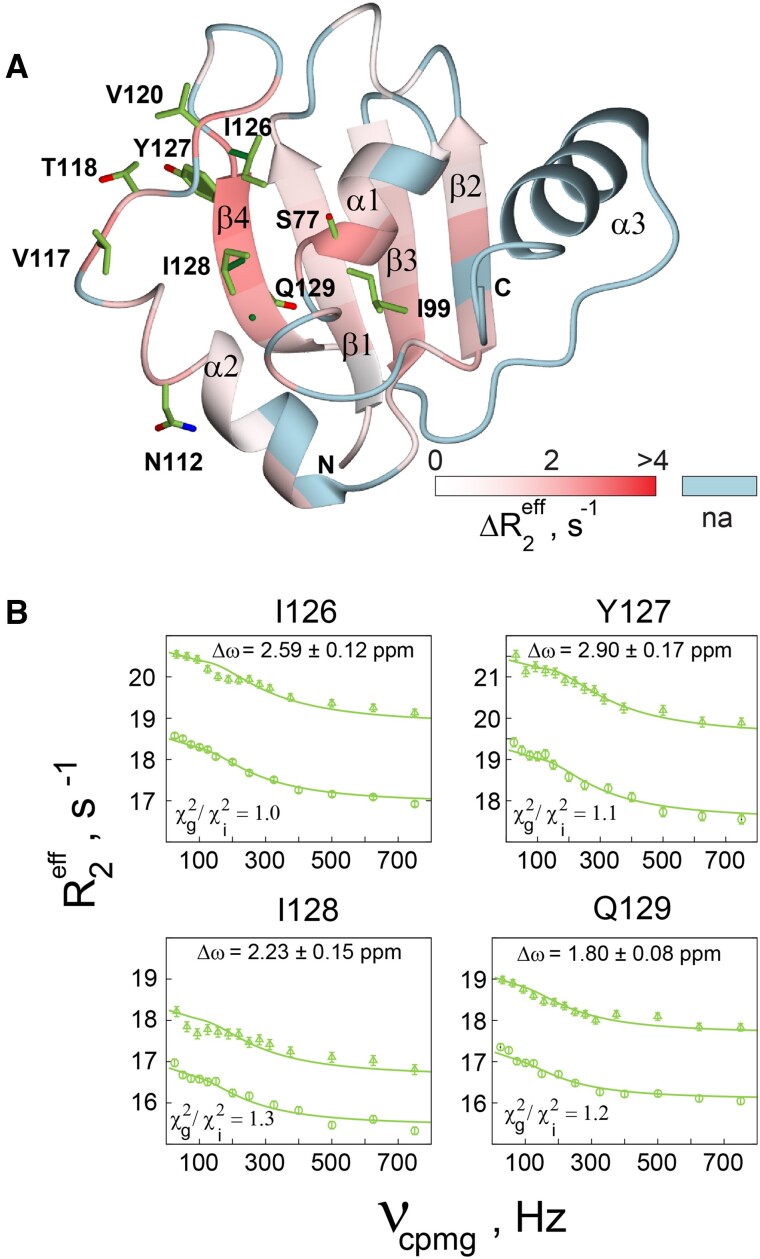
^15^N CPMG dispersion data of PTB RRM at 313 K. (**A**) Δ*R*_2_^eff^ =R_2_^eff^(υ_cp_= 25 Hz) − *R*_2_^eff^(υ_cp_= 750 Hz) measured for domain residues on a 750 MHz spectrometer mapped on the structure of PTB RRM1. Sidechains of residues analyzed by the general exchange equation are shown. (**B**) CPMG curves measured on 750 (circles) and 900 MHz (triangles) for the residues of the β4 strand in PTB RRM1. Solid curves correspond to global fits of the two-site general exchange equation. Chemical shift difference of the exchanging states, (Δω), and the ratio of χ^2^ values for global and individual fits (χ^2^_g_/χ^2^_i_) are indicated.

To obtain quantitative information about the slow exchange events mentioned above in the domain, we carried out CPMG relaxation dispersion experiments of the free PTB RRM1 at 313 K. While the N- and C-terminal residues are severely exchange broadened, preventing their analysis, many domain residues display relaxation dispersion, revealing μs−ms time-scale exchange events across the protein (Figure 3A and Supplementary Figure S3A). For those CPMG dispersion profiles, which could be fitted using both the two-site fast exchange equation and the two-site general exchange equation (Supplementary Figure S3B and S3C), fits to the general exchange equation, were found to be significantly better when applying the Akaike information criterion (Tables S3 and S4). Most of these residues are located in the α2−β4 loop and β4, and give large chemical shift differences of the exchanging states, Δω, in the range 1.5−4.0 ppm (Figure 3A, Supplementary Table S4). All the residues of the β4 strand show significant exchange and could be fitted globally yielding an exchange rate of 892 ± 53 s^−1^ (Figure 3B, Supplementary Table S4). The Δω values from the fits are similar to the difference between chemical shifts in the wild type protein and random coil shift values, suggesting that β4 residues transiently visit a random-coil like-environment. β4 is flanked by highly flexible loops and shows only one slowly exchanging amide which H-bonds to the adjacent strand (12), thus allowing it to explore a large conformational space in the free protein. Consistent with this, analysis of the backbone chemical shifts in this region indicate lower β-strand probability (Supplementary Figure S4) (66). In summary, characterization of slow exchange in the PTB RRM1 domain at 313 K revealed slow timescale motions in loop residues following α2, as well as in the β4 strand due to ms timescale exchange with random coil-like conformations. This may be related to the unusually large internal motion correlation times found in α2 (Figure 2C).

Altogether our dynamics study, reveals the free RRM1 is comprised of a compact rigid fold encompassing β1−α1−β2−β3, which is flanked by a very flexible upstream N-terminal part and a partially disordered downstream region extending from the α2−β4 loop up to α3, showing conformational dynamics on a range of timescales from ps−ms.

### Slow motions at the binding interface of the PTB RRM1-SL UCUUU complex

**Figure 4.**
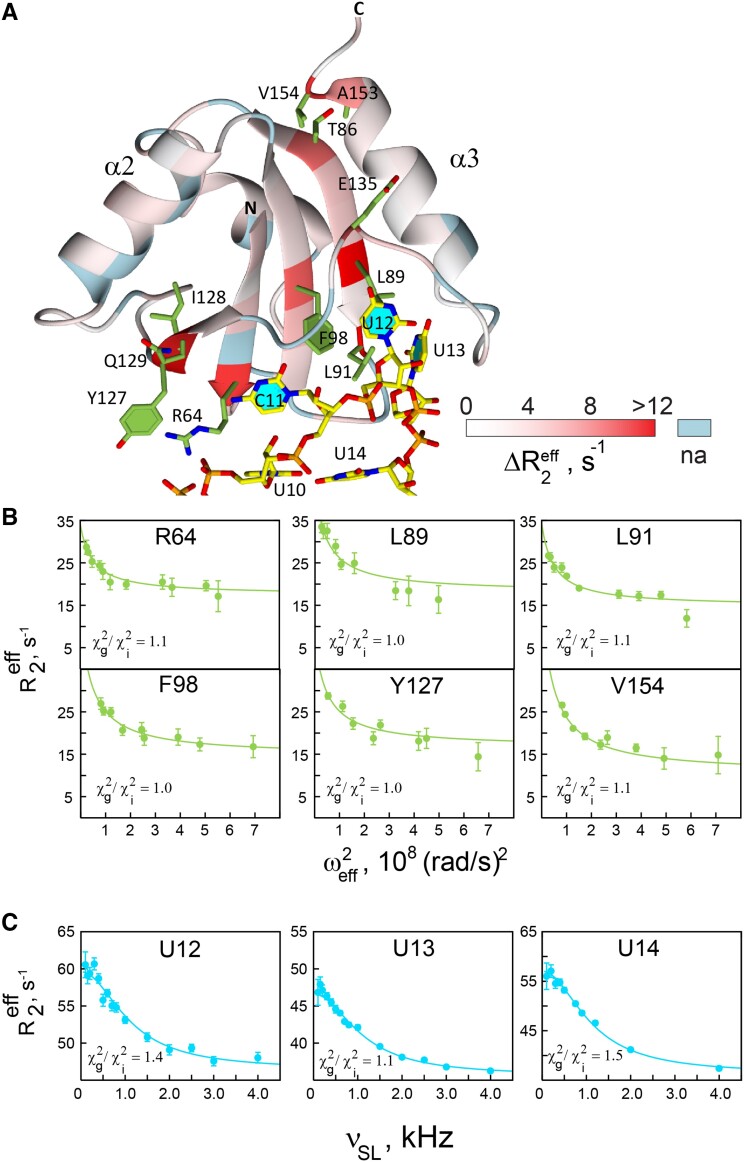
^15^N and ^13^C *R*_1ρ_ dispersion data of PTB RRM1-SL UCUUU complex at 313K. (**A**) Slow motions detected with Δ*R*_2_^eff^= *R*_2_^eff^(ω^2^_eff_= low) − *R*_2_^eff^(ω^2^_eff_= high) are indicated by a color scale projected onto the ribbon representation of the protein backbone; residues of RRM1 and the nucleotides of SL UCUUU with Δ*R*_2_^eff^> 5 s^−1^ are shown in green and cyan respectively. (**B**) ^15^N *R*_1ρ_ dispersion curves for some of the residues of PTB RRM1. (**C**) ^13^C *R*_1ρ_ dispersion curves for C1' sites in SL UCUUU RNA. Solid curves in (B) and (C) represent the global fit of the two-site fast exchange model (Eq. 3) to the experimental data obtained with a *k*_ex_ value of 6923 ± 229 s^−1^.

Slow motions in the complex were characterized with *R*_1ρ_ (*R*_2_) measurements by varying the spin-lock field strength or irradiation offsets (see Methods). A survey of relaxation dispersion in the protein shows strong *R*_ex_ contributions to *R*_2_ for residues Arg64, Thr86, Leu89, Leu91, Phe98, Tyr127, Ile128, Gln129, Glu135, Ala153 and Val154 in the RNA bound state (Figure 4 and Supplementary Figure S5). Except for Thr86, Ala153 and Val154, these residues are at the RNA binding interface and interact with the apical loop of the RNA. The millisecond (ms) exchange observed by CPMG dispersion measurements in the free protein for residues 75−78 of the α1 helix and residues 117−121 following the α2 helix (Figure 3A, Supplementary Figure S3) is quenched on RNA binding (Figures 4A and S5A). The absence of slow fluctuations in α1 (which is distant from the binding interface) upon binding to the stem–loop, could be due to stabilizing contacts with the well-folded C-terminal helix.

*R*_1ρ_ dispersion measurements were also carried out for ^13^C nuclei in SL UCUUU by conventional two-dimensional methods and one-dimensional methods based on selective excitation of the ^13^C of interest. In the bound state of SL UCUUU, dispersion is observed only for the C1′ carbons of residues in the apical loop, U12, U13 and U14 (Figure 4C). The C1′ signal of U10 and C11 are severely exchange broadened and their relaxation rates could not be measured accurately. None-the-less, this broadening also indicates slow motions for the sugars of these nucleotides. The protonated carbons of the loop's nucleotide bases do not show significant differences in *R*_1ρ_ rates measured at extreme values of the spin-lock effective fields and the dispersion profiles are flat (Supplementary Figures S6B and S7C). Slow exchange motions are also absent for both sugar and nucleotide base carbons of SL UCUUU in the free state (Supplementary Figures S6A, S7A and B). The slow exchange events detected in both the protein and RNA backbone upon binding are thus primarily localized at the binding interface (Figure 4A).

The ^15^N *R*_1ρ_ relaxation dispersion data were analyzed by fitting the two-site fast exchange model (Eq. 3, Methods) individually for each residue of PTB RRM1 to determine the parameters ϕ^SL^_ex_ and k_ex_ (Supplementary Figure S5B). The individual fits gave similar estimates of exchange rates for the different residues except Glu135, which could not be fitted satisfactorily (Supplementary Table S5). Separate fits of the two-site fast exchange model to the *R*_1ρ_ dispersion data of the C1′ carbons of RNA yielded similar estimates of *k*_ex_ for U12, U13 and U14 (Supplementary Figures S8 and S9). The *k*_ex_ values estimated for the C1′ sites are also close to those estimated from the ^15^N *R*_1ρ_ data for PTB RRM1 residues in the bound state (Supplementary Table S5). A simultaneous fit of the two-site fast exchange model resulted in a common exchange rate for the ^15^N *R*_1ρ_ dispersion data of PTB RRM1 and ^13^C *R*_1ρ_ data of SL UCUUU in the bound state of *k*_ex_ = 6923 ± 229 s^−1^ (Figure 4B and C). The ratio of χ^2^ values for the global and individual fits was <1.6, with the exception of A153 (χ^2^_g_/χ^2^_i_ = 2.3) which was therefore not included in the estimate of a global *k*_ex_ value (Supplementary Table S5).

It is interesting that slow exchange in the RNA loop occurs at the C1′ sites but not for ^13^C sites on the nucleotide bases. The nucleotide base of U12 stacks against residue Leu89 and its Watson-Crick face is hydrogen bonded to the backbone NH and C=O of Lys137. U13 interacts with the sidechains of Leu89, Leu91 and Asn103, while U14 forms a mismatched base pair with U10, which stacks over the G9–C15 base pair (Figure 4A). These interactions could restrict motions of the bases of U12, U13 and U14 and may account for the lack of slow exchange events detected at the base carbons. For the following reasons it is unlikely that the global μs dynamics at the binding interface is due to exchange between the free and bound state. First, the chemical shift difference for the C6 and C1′ carbons in the free and bound state range from 25−125 Hz at 500 MHz, which is too small to account for the observed exchange. Second, the exchange parameter ϕ^SL^_ex_ does not show a linear dependence on the square of chemical shift difference between the bound and free states as predicted by the fast exchange equation (Eq. 3) in this scenario (Supplementary Figure S10). In summary, global exchange caused by slow motions on ms−μs timescale appear in the backbone at the protein/RNA interface upon complex formation (Figure 4), whereas on timescales below the global correlation time, extensive dynamics in the protein is quenched, particularly at the protein/RNA interface (Figures 2 and 3).

### PTB RRM1 has a partially folded α3 helix, which transiently unfolds

The major structural change, which occurs in PTB RRM1 on interaction with SL UCUUU, is the formation of helix α3 at the C-terminus and its interaction with the rest of the domain. We wondered whether the presence of a transiently formed α3 helix in the unbound protein could be causing the slow dynamics observed in the rest of the domain. In the SL UCUUU-bound form of PTB RRM1, the α3 helix interacts with the RRM via contact between Ala147, Leu151 and Val154 in α3 and Ile76 in α1, and Val85, Thr86 and Leu88 in β2 from the domain. If the slow exchange events in the domain and the presence of a transient α3 helix in the free state are correlated, mutations, which disfavor α3 helix formation and/or helix contacts with the domain, should alter the dynamics of the domain residues. The point mutant L151G of PTB RRM1 was shown to have a disruptive effect on the formation of the C-terminal helix as ^13^C secondary shifts indicated near random coil character for this C-terminal region (12). We therefore compared the slow timescale dynamics of this mutant with that of the wild-type RRM1.

**Figure 5.**
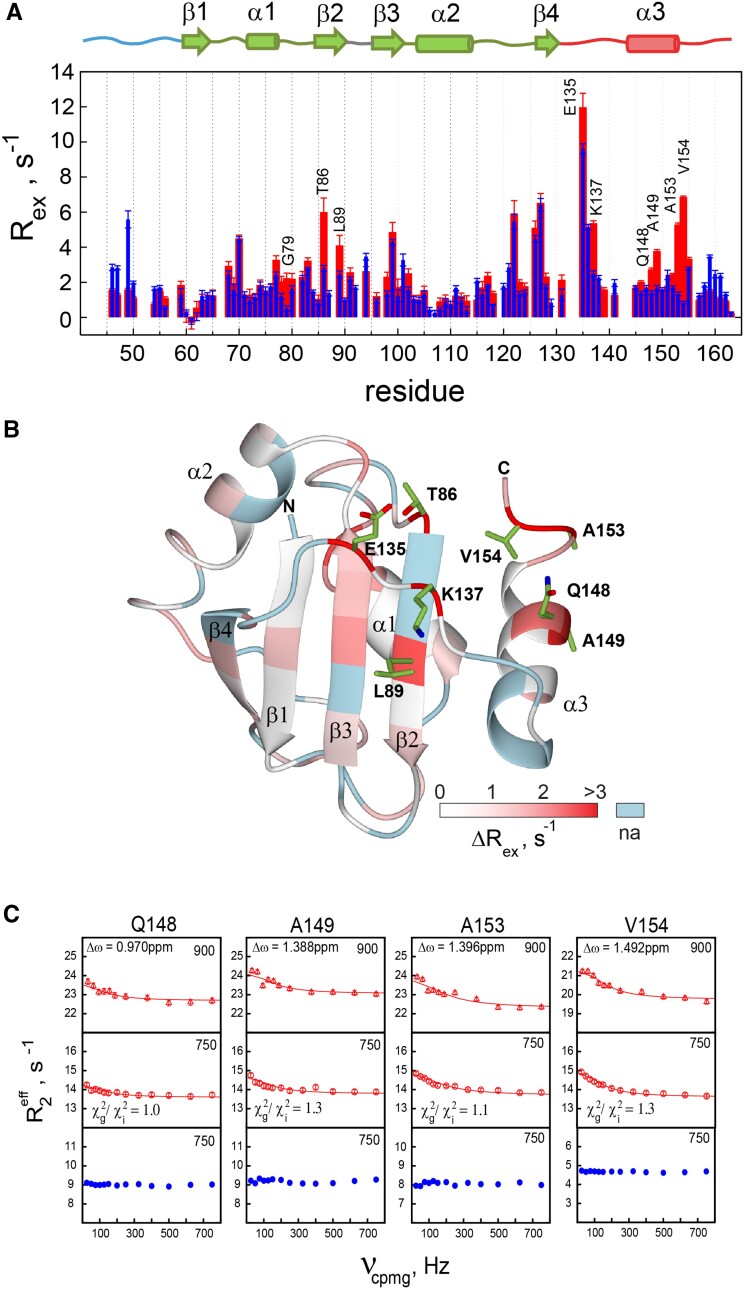
Relaxation dispersion differences between PTB RRM1 and PTB RRM1[L151G]. (**A**) Qualitative R_ex_ map obtained from RCZZ I_z_S_z_ experiment for wild type PTB RRM1 (red) and its L151G mutant (blue). (**B**) Regions with enhanced *R*_ex_ (Δ*R*_ex_) in the wildtype protein relative to the L151G mutation displayed on a ribbon drawing of residues 58–155. Sidechains of residues showing a Δ*R*_ex_ of more than 1.5 s^−1^ are displayed. (**C**) CPMG relaxation dispersion data obtained for the C-terminal residues of the wild type PTB RRM1 (in red) and L151G mutant (in blue). Solid line is the global fit of the general exchange equation to WT data from the four residues at two fields (750 MHz and 900 MHz). The parameter Δω was fixed to the ^15^N chemical shift difference between wild type and L151G mutant. Ratios of χ^2^ values for global and individual fits (χ^2^_g_/χ^2^_i_) are indicated.

An efficient way of obtaining a qualitative map of *R*_ex_ in proteins employs the Hahn echo to detect very slow exchange contributions to *R*_2_, which may be suppressed by CPMG pulse trains or spin-locking fields due to their relatively high effective spin-lock strength compared to the exchange rate. The Hahn echo-based relaxation compensated-I_z_S_z_ (RCZZ) method was applied to obtain a qualitative *R*_ex_ map for PTB RRM1 and its L151G mutant in the free state. Strong exchange broadening was observed for residues in the C-terminal region of PTB RRM1 at 313K, preventing quantitative analysis of RCZZ experiments, which employ long Hahn echo periods. We therefore measured *R*_ex_ in the wildtype protein at 298 K. As seen in Figure 5, extensive exchange contributions to *R*_2_ (*R*_ex_) occur throughout the wild-type protein. Whereas Ile126 and Tyr127 of the β4 strand have similar *R*_ex_ in the wild type and mutant forms of PTB RRM1, significant reductions in *R*_ex_ are observed in the L151G mutant for Ser77−Gly79 in α1, Thr86, Leu89 of the β2 strand, and Glu135, Lys137, Gln148, Ala149, Ala153 and Val154 in the C-terminal region (Figure 5A, Supplementary Table S6).

Interestingly, residues 148−154 are part of the segment, which forms the α3 helix, and Thr86 and Leu89 are adjacent to residues Val85 and Leu88 in β2, which contact Leu151 in the SL UCUUU-bound state. The residues Gln148 and Ala149 are located one helical turn before the mutation site, while Ala153 and Val154 are positioned one turn after it. These two pairs of residues are also positioned across from Leu89 and Thr86 in the complex, respectively (Figure 5B). In contrast, for residues Glu135 and Lys137, which are more remote from the α3 helix region and the β2 strand, although *R*_ex_ shows a decrease, significant exchange is still present in the mutant protein. The reduction in *R*_ex_ caused by the L151G mutation clearly indicates that these slow exchange events in the domain residues and in the C-terminal part (α3 helix) are correlated. These qualitative *R*_ex_ data are supported by comparison of CPMG dispersion curves obtained for the C-terminal residues of the wild type and L151G mutant (Figure 5C and Supplementary Figure S11). The ms exchange contribution to *R*_ex_ in the helix-forming C-terminal residues of the wild type protein are quenched in the L151G mutant. Altogether, these relaxation data suggest that the slow exchange observed for the C-terminal residues could correspond to an exchange equilibrium involving a partially organized α3 helix contacting the domain and a random coil conformation.

The observed CPMG dispersion curves for the C-terminal residues of PTB RRM1 are weak making it difficult to extract all the kinetic parameters reliably from a fit of the general exchange equation (Eq. 2, Methods). The parameter Δω was therefore set to the chemical shift difference between the wild type protein and the L151G mutant, which represents states with a partially organized C terminus and a random coil structure respectively. Making this assumption, the exchange rates could be obtained from fits of the dispersion data at two fields to each of the four C-terminal residues. The exchange rate *k*_ex_ ranges from 683 to 1095 s^−1^ with the minor state population <0.5% (Supplementary Table S7). The data at the two fields confirm this result as the four C terminal residues could also be fitted globally with an exchange rate of 837 ± 80 s^−1^ and a population of the minor state of *p*_b_= 0.33 ± 0.02% (Figure 5C). In summary, the *R*_ex_ of the C terminal residues in the wild-type protein are likely caused by exchange between random coil and a partially formed α3 helix structure with the latter being more populated. The *R*_ex_ seen for residues Ser77−Gly79, Thr86 and Leu89 (Figure 5A) can be explained by the loss of contacts with α1 and β2 of the domain accompanying the transient unfolding of the partially folded C terminal helix. We note that an alternative model, in which exchange occurs between a fully formed α3 and random coil state is excluded since the Δω values derived from the chemical shift differences between the PTB RRM1-SL UCUUU complex and L151G mutant are too large to be fit well by the CPMG data (Supplementary Table S7). Consistent with this exchange-broadening in wildtype PTB RRM1 being caused by transient contacts between α3 and the domain, we also observe ^13^C relaxation-dispersion in the side chains of key residues of the domain forming the interface in the complex, such as Ile76, Val85 and Leu89 (Supplementary Figure S12).

### NOESY spectra detect dynamic α3 in free PTB RRM1 contacting the domain

Both NMR relaxation characterizing fast and slow motion shown above suggested the presence of a partially folded helix in the region C terminal to the RRM that contacts the folded domain. Similarly, backbone chemical shifts indicated the presence of a helical population for residues 144−154 (Supplementary Figure S4A) and suggested docking of this region to the domain (12). We wished to confirm this by structural characterization with NMR. We note that previous NMR structural studies of free PTB RRM1 did not include the entire region of the α3 helix (residues 55−147) which would likely prevent the formation of a transient helix (67). We therefore measured ^13^C- and ^15^N-resolved NOESY spectra of PTB RRM1 at 298K with high resolution in the indirect dimensions to facilitate identification of NOEs in the poorly dispersed C-terminal region. We found NOEs indicating both a helical conformation for residues Gln144−Val154 (Supplementary Figure S13) as well as long-range contacts between the folded domain and this helical region (Supplementary Figures S14−S16). Systematic inspection of the NOESY spectra of the free protein revealed crosspeaks (or intensity in overlapped regions) supporting contacts between RRM1 and helix α3 similar to those observed in the RRM1-SL UCUUU complex. We therefore performed a structure calculation using peaklists derived from these NOESY spectra and the complete assignments of the free protein. Due to the dynamic nature of the free protein, we used the consensus-structure calculation method to sample more effectively the full extent of the structural ensemble. In the consensus-structure method, 20 independent structure calculations are performed with independent automated assignment of the NOEs and an ensemble is then derived from the combination of the single best conformer from each of these calculations (the combined ensemble). A final calculation is then performed using the constraints occurring most consistently in the individual calculations to obtain the consensus ensemble. (see Methods) (48). In total, 22 unambiguous contacts between the RRM and the α3 region could be detected in the NOESY spectra (Supplementary Figures S14−S16, Supplementary Table S8). The ensemble revealed a well-defined RRM with the same fold as for the complex, but with a slight straightening of the β4–α3 loop, and a less precisely defined helix α3 contacting the domain (Supplementary Figures S17 and S18, Supplementary Table S8, Figure 2B versus 2D). The lower precision of the helix in the free protein is probably due to the high ambiguity of the constraints in this region caused by the significantly lower chemical shift dispersion of residues in the flexible C-terminal part. It is interesting to note that even though PTB RRM1 has structural features similar to PTB RRM1-SL UCUUU, the structure is less precisely defined in the regions of PTB-RRM1 which show extensive dynamics, for example the α3 helix, the C-terminal end of α2 and the long loop following it (Supplementary Figure S18). In summary, dynamics data indicate partial ordering in the C-terminal region of PTB RRM1 (Figure 2, Supplementary Figure S2A), and its transient interaction with β2 and α1 of the RRM domain (Figure 5). Analysis of NOESY spectra confirm the presence of a partially ordered α3 helix which docks to the RRM domain in a manner similar to what is seen in the PTB RRM1-SL UCUUU complex (Figures 2B versus D, S14−S18) (12).

### MD reveals protein–RNA interface transitions, which are inaccessible in the free protein

NMR relaxation provided insight on the location, extent, and timescale of dynamics in the protein and RNA, and indicated the presence of a partially ordered and docked α3 helix in the free protein. However, it did not show us how RNA binding is coupled to the dynamic equilibrium controlling α3 helix ordering. We therefore performed MD simulations to obtain structural insight on this transition. Standard MD simulations of PTB RRM1 bound to SL UCUUU showed that the structure of the complex is stable, remaining within 2.5 Å RMSD of the average NMR backbone coordinates (S58−N155) and satisfying the vast majority of the NMR restraints, even on a 10 μs timescale (Tables S9 and S10). Similar performance was observed for simulations of the free protein starting from coordinates of PTB RRM1 with the RNA removed, which remained within 3.7 Å RMSD of the average NMR structure backbone coordinates for the entire 10 μs simulation. To investigate the role of the L151G mutation in disrupting the α3 helix we also performed simulations of the L151G mutant alone and bound to the SL RNA. Starting coordinates were derived from the respective coordinates of the wildtype protein. In simulations of the L151G mutant protein bound to SL UCUUU, we observed several partial disruptions of the α3 secondary structure associated with the mutation site. These were all reversible on the simulation timescale. A similar process was observed in simulations of the free L151G mutant, except the α3 disruptions were more large-scale than in the complex and irreversible by the simulation end. In both wildtype free protein and the complex, we observed fluctuations for the β4−α3 and β2−β3 loops (Supplementary Figure S19), which form part of the protein/RNA interface in the complex.

**Figure 6.**
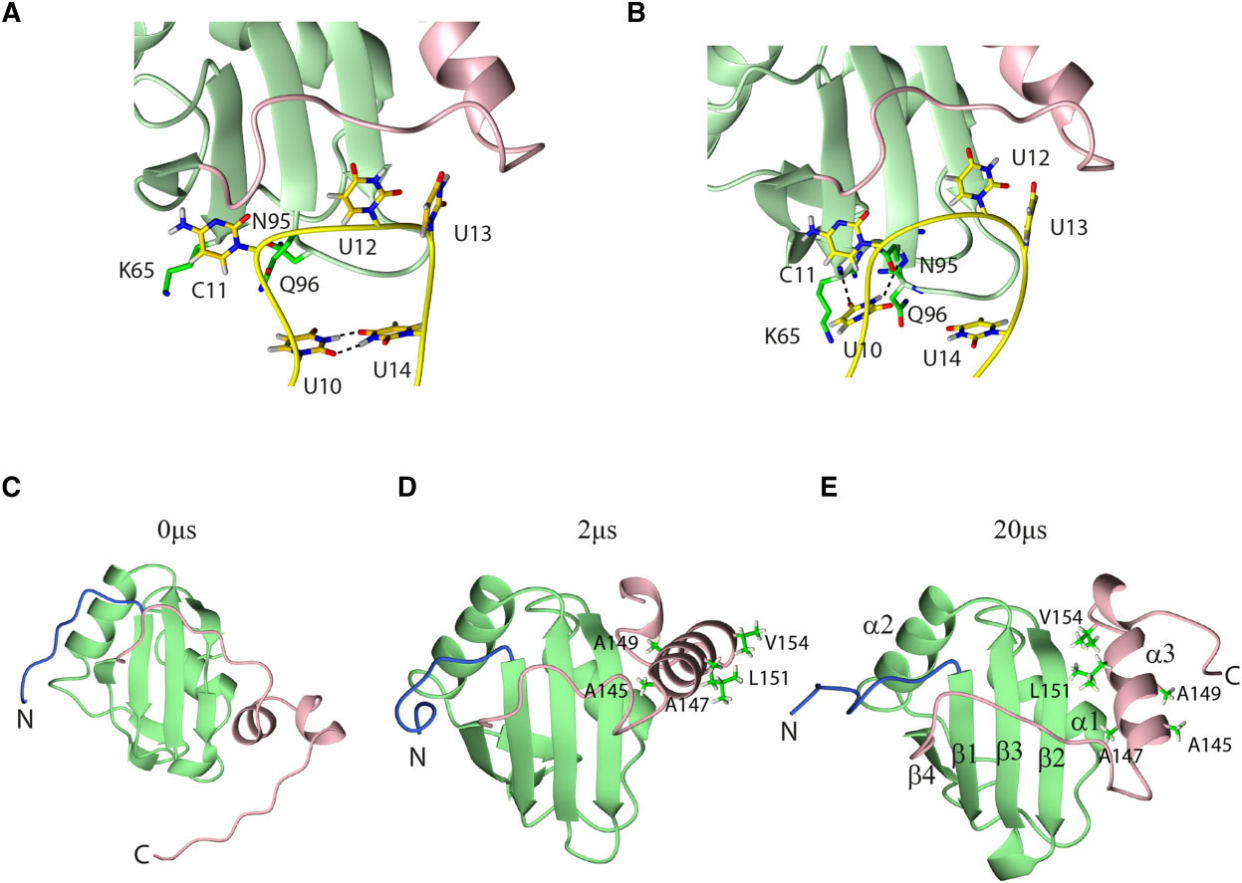
Standard MD simulations of free and bound PTB RRM1. (A–B) MD simulation of PTB RRM1-SL UCUUU illustrating opening of the U10−U14 basepair. (**A**) SL UCUUU bound to PTB RRM1 at start of simulation, and (**B**) after opening of U10−U14 base pair. U10 makes interactions with residues K65, N95 and Q96. (C–E) MD snapshots of PTB RRM1 conformer 3 from the combined ensemble at start of simulation (**C**), after 2 μs (**D**), and after 20 μs (**E**). Helix α3 folds, taking on a noncanonical docking to the domain and later is positioned as in the complex.

In the case of the complex, simulations revealed that dynamics of these protein loops was strongly influenced by the interactions formed between the U13 base and the β4−α3 loop residues just N-terminal to the α3 helix. Transient disruptions of these intermolecular interactions occurred in simulations due to fluctuations of either the α3 helix or the bound RNA. During some of these disruptions, we observed that the L136 sidechain within the β4−α3 loop shifted position and perturbed the conformation of the F98 side-chain. The χ1 dihedral angle of F98 was always in the gauche(−) region in the free protein but in the complex it fluctuated between gauche(−) and gauche(+) in response to RNA-related dynamics of the β4−α3 loop. In the NMR structure of the complex, the F98 side-chain is in contact with the L89 and L91 side-chains, as well as the ribose of C12. Possibly the μs dynamics of these residues in the complex detected by NMR correspond to the transitions revealed by our simulations (Figure 4A and B, Supplementary Figure S5). The second structural change observed in simulations was the transient loss of the U10−U14 base pair (Figure 6A and B). This sometimes occurred also in the free RNA (Supplementary Table S9) but was much more prevalent in the complex. Transient loss of this base pair is supported experimentally by the absence of imino signals for the U10−U14 base pair above 10C. During these disruptions, U10 typically formed H-bond interactions with the N95 backbone and stacking interactions with the R64 side-chain whereas U14 moved towards the U13 nucleotide and transiently interacted with it (Figure 6B). The sugar pucker of U14 was C3′-endo when the base pair U10−U14 was formed and transitioned between C2′/C3′-endo when it was disrupted. The sugar puckers of the other pentaloop nucleotides were usually in C2′-endo. These structural transitions involved residues showing μs dynamics (Figure 4). Sugar pucker transitions can be dramatically slowed down when coupled to large-scale structural transitions such as RNA secondary structural rearrangements and sugar carbons relaxation-dispersion can report on such slowed dynamics (68). We found that in both cases, the observed transitions were related to the dynamics of the U13 base and the N-terminal part of helix α3, either by inducing changes in the β4−α3 loop or by providing an alternative binding site for the U14 base when the U10−U14 base pair was transiently lost. This was intriguing since previous work showed that α3 helix content was strongly influenced by the binding of SL RNA, and was sensitive to the sequence of the bound pentaloop, despite there being no direct contacts between α3 and RNA (12).

### Standard MD Simulations reveal conformational heterogeneity of α3 helix in free PTB RRM1

The simulations of PTB RRM1 bound to SL UCUUU and of the free PTB RRM1 started from the complex structure with RNA removed revealed virtually the same degree of helix α3 ordering. This contrasts with the NMR data that indicated reduced α3 ordering in the free protein (Figure 2). The standard MD simulations managed to describe the global movements of helix α3 and their relation to protein-RNA interface dynamics (see above). However, it is likely that even at the 10-μs-timescale, standard MD is insufficient to capture differences in helix α3 ordering when starting from an initial structure where the helix is fully folded. Therefore, to explore the less ordered α3 conformations of the free protein, we next performed simulations starting from different conformers of the combined NMR ensemble of the free protein (see Methods), which showed a greater variety of α3 conformations than the complex. We started two independent 2 μs simulations from three different conformers of this ensemble, namely 1,3, and 10. In conformers 1 and 10, α3 is fairly well formed but with different domain contacts, whereas in conformer 3 it is only partly folded (Figure 6C). In simulations started from conformers 1 and 10, α3 remained folded (Supplementary Figure S20). However, we observed a divergent behavior for the two simulations started from conformer 3. In the first simulation, the partially helical segment completely unfolded (Supplementary Figure S20), whereas in the second simulation it propagated to a fully folded α-helix with alternative contacts to the domain (Figures 6D, S20). Whereas in the structure of the complex determined by NMR, α3 made hydrophobic contacts to the RRM mediated by Ala147, Leu151 and Val154, in the simulation, α3 was rotated so that Ala145 and Ala149 formed RRM contacts instead. We therefore extended this simulation to 20us. The helix remained stable in this conformation for 6 μs before these contacts were disrupted and the helix then sampled a large variety of interactions with the RRM domain for the next 10 μs. Thereafter, α3 established the contacts observed in the complex, and it remained in this position for the rest of this simulation (Figure 6E). In conclusion, this simulation of the free protein suggested that the positioning of α3 with respect to the RRM is less well defined when not constrained by the bound RNA, allowing the α3 segment to sample more diverse positions, some of which might be less supportive of a helical conformation. The ability of helix α3 to undergo such large-scale rearrangements provides a plausible rationale for the slow exchange seen in the free protein for residues of α1, β2, the β4−α3 linker and α3 (Figure 5) as well as the relaxation-dispersion detected for sidechains at the RRM-α3 interface (Supplementary Figure S12).

### Enhanced sampling simulations reveal helix α3 ordering is sensitive to the identity of the bound RNA sequence

**Figure 7.**
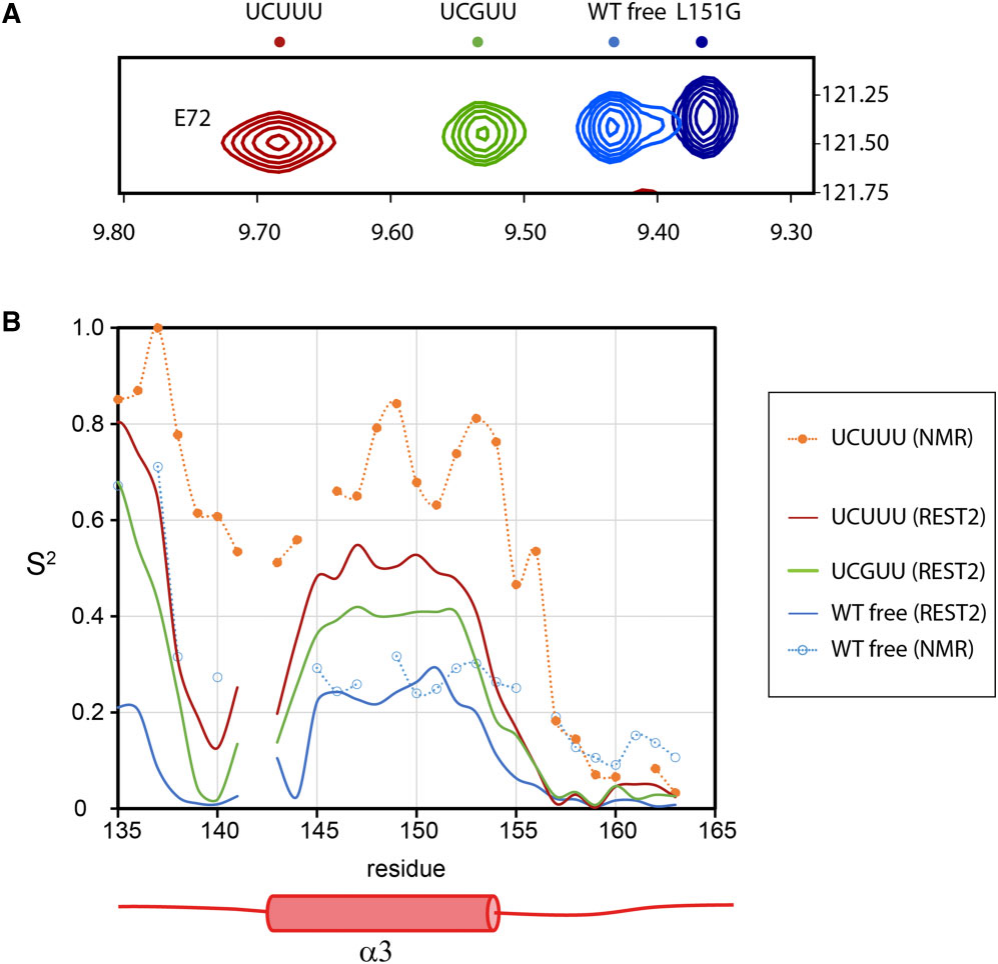
(**A**) Overlay of [^15^N,^1^H]HSQC spectra for wildtype PTB RRM1, free and bound to SL UCUUU and SL UCGUU, and the L151G mutant, showing the backbone signal of E72 which reports on degree of α3-docking to the RRM. The level of α3 helix formation ranges from zero (L151G on the right) to the maximum (SL UCUUU complex on the left). (**B**) NH bond order parameters of the C-terminal region calculated from all trajectories of REST2 simulations of PTB RRM1 unbound, and bound to SL UCUUU and SL UCGUU, compared to model-free NH order parameters obtained from NMR relaxation data.

NMR experiments revealed a relationship between helix α3 ordering and identity of the bound SL RNA sequence. Replacement of individual Uridines in the UCUUU pentaloop by Guanine reduced the α3 helix ordering as judged by chemical shift changes (12). We wished to explore the structural and dynamic basis of this phenomenon by performing molecular dynamics (MD) simulations of PTB RRM1 bound to different SL RNAs. Overall, the standard MD simulations of the complex demonstrated that both the protein/RNA interface and the C-terminal α3 helix are stable and well described with the used force fields. However, standard MD simulations were unable to observe a change in helix α3 ordering in the sampled simulation time even upon complete removal of the RNA (see above). Therefore, we performed REST2 enhanced sampling simulations focused on the α3 helix region of the free PTB RRM1, as well as its complex with SL UCUUU or SL UCGUU RNAs, respectively. In these simulations, several replicas of the system are simulated: the reference replica uses the standard force field and each of the other replicas simulates the system with reduced energy barriers in the region of interest (residues 140 −156). This allows larger structural transitions to be sampled than for standard simulations (see Methods for details). We observed clear differences in helix α3 ordering in these simulations. Whereas α3 was completely unfolded at the end of five out of six continuous REST2 trajectories of the free protein, in the SL UCUUU complex only one continuous REST2 trajectory showed α3 unfolding on the identical timescale (Supplementary Figure S21). The average degree of order of the α3 helix in the reference replica with the standard energy potential, as expressed by order parameters of the backbone NH groups, was uniformly high in both the free and SL UCUUU-bound RRM1. However, when all REST2 replicas were analyzed, thereby including the simulation trajectories with reduced energy potentials in the α3 region, we observed a pronounced decrease of order for α3 in the free versus RNA-bound RRM1 (Figure 7). Even in the reference replica, the α3 order parameters showed a pronounced decrease in later parts of the free PTB RRM1 REST2 simulations while remaining essentially unchanged for the complex (Supplementary Figure S22). Since the order parameters of the reference replica were still changing at the end of the REST2 simulation full quantitative convergence of the helix α3 dynamics was not achieved even on the 10 μs timescale of these REST2 simulations. None the less they are sufficient to visualize relative differences between the free and bound protein. Importantly, there is clear consistency in the trends observed for the α3 order parameters obtained by simulation with those measured by NMR for the free and the SL UCUUU-bound protein (Figure 7).

The REST2 simulations where we replaced U12 in the SL UCUUU by guanine (SL UCGUU) revealed that α3 unfolded in two of six continuous REST2 trajectories (Supplementary Figure S21). Analysis of the average NH order parameters showed a smaller degree of α3 order (Figure 7) and a larger decrease of order was observed in the reference replica in later stages of the SL UCGUU REST2 run compared to the corresponding SL UCUUU data (Supplementary Figure S22). This is consistent with the NMR experiments, which showed that this U to G variant of the stem-loop caused the smallest degree of α3 helix ordering upon binding to RRM1 as assessed by the relative magnitude of chemical shift changes upon binding (Figure 7A) (12).

### U12G substitution in SL UCUUU destabilizes the H-bonding network at the RRM-α3 interface

**Figure 8.**
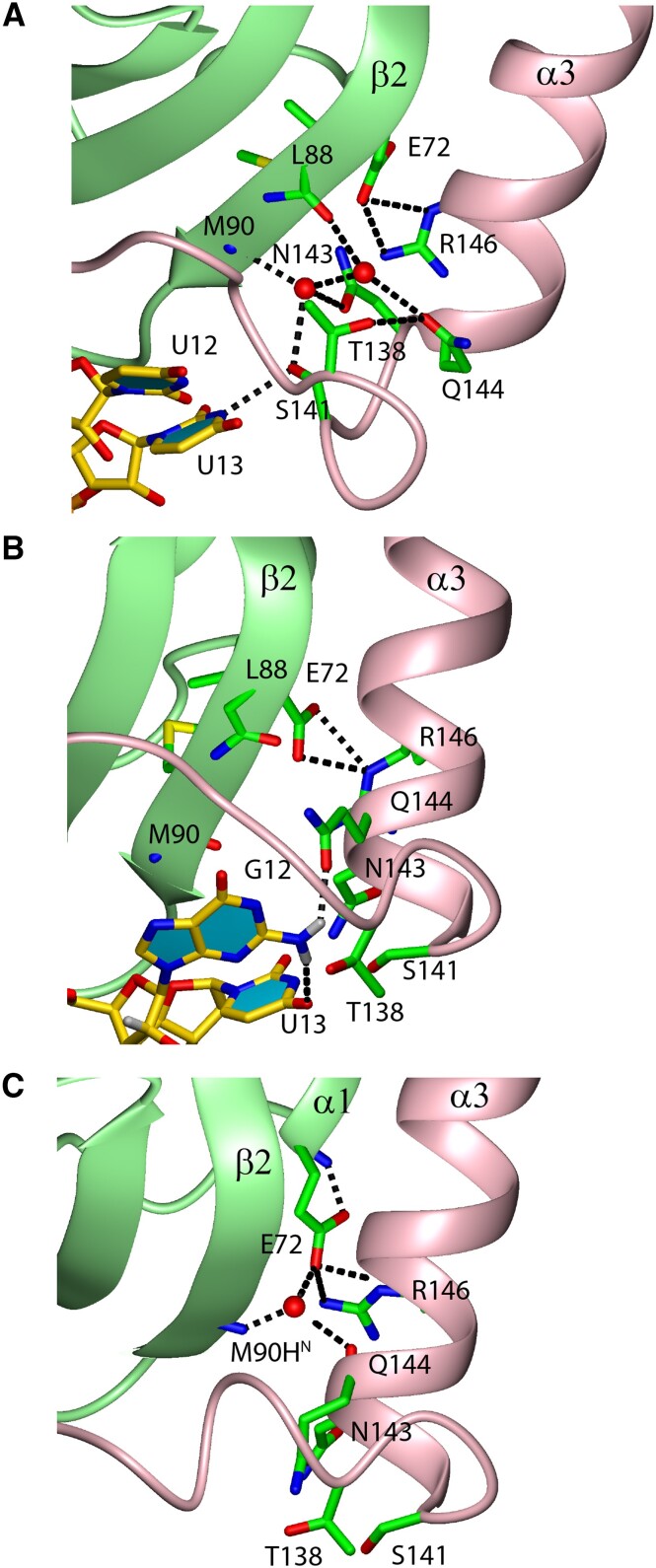
Comparison of H-bonding networks in MD simulations of PTB RRM1 in complex with SL UCUUU (**A**), SL UCGUU (**B**), and unbound PTB RRM1 (**C**) at the interface between the protein domain and the α3 helix. In the UCUUU complex, this network includes several waters, which mediate the interface contacts. The 2-amino group of G12 interferes with all of these interactions. In the free protein, increased disorder in the N-terminal loop preceding α3 due to the missing contacts with U13 results in less specific docking, and a more dynamic helix.

To understand why α3 is less ordered when U12 is substituted by G12 in the loop sequence, we analyzed the interactions between the RNA, the RRM, and the α3 helix region in our simulations. We found that SL UCUUU binding resulted in a network of H-bonds between U13 and residues just N-terminal to the α3 helix, which are also complemented by solvent molecules forming water bridges at this interface (Figure 8A). The sidechain of S141 typically formed an H-bond with the U13 N3 or O4 atoms. At the same time, it also H-bonded with a water molecule, which coordinated Met90HN in β2, the Asn143 sidechain, and a second water molecule. This created a network of H-bonds connecting the β4−α3 loop, β2, and the first helical turn of α3 (Figure 8A). Consistent with the formation of these stabilizing interactions, this part of the β4−α3 loop shows a prominent increase of NMR-derived order parameters when SL UCUUU is bound (Figure 2, Supplementary Table S2, Figure 7B). In contrast, in both the standard and REST2 simulation of the SL UCGUU complex, G12 displaces U13 from its original binding site, disrupting U13 H-bonding interactions with the β4−α3 loop and forming an intramolecular G12 N2/U13 O4 interaction or a G12 N2/Q144 OE1 intermolecular interaction. (Figure 8B). This also abolished the network of water-mediated interactions seen in the SL UCUUU complex. In conclusion, simulations indicate that loss of the interaction network involving U13, solvent molecules, the β4−α3 loop, β2, and the first helical turn of α3 are responsible for the experimentally observed reduced α3 helicity when U12 is replaced by G12 (12). Lastly, we looked for differences related to dynamics of the interface residues of the SL UCGUU complex compared to those observed in simulations of the SL UCUUU complex. The RNA related changes in the conformation of the β4−α3 loop greatly increased the frequency of F98 side-chain χ1 flips between gauche(−) and gauche(+), and the gauche(+) region was now preferred. In simulations of the SL UCUUU complex, the gauche(+) conformation of F98 was associated with transient disruptions of U13 binding to the region N-terminal to α3 and subsequent changes in β4-α3 loop conformation. This also showed how RNA binding can lead to enhanced microsecond dynamics that was detected for F98 and U13 (Figured 4B and 4C). It is yet another indication of the close relationship between the β4−α3 loop and helix α3 conformations. Furthermore, it is fully consistent with reduced α3 helicity observed for SL UCGUU system. The shift in rotamer populations for F98 upon binding to different SL RNAs is reminiscent of dynamic adaption of rotamer populations at the binding interface of RRMs upon binding to different RNA sequences observed previously (69). We did not observe changes in dynamics for the U10−U14 base pair in the SL UCGUU REST2 simulations on our simulation timescale.

### U13 helps maintain the register of α3-RRM interactions

The simulations indicated that the interactions between U13 and the β4−α3 loop segment just N-terminal to α3 are important to maintain the global position of α3 in relation to the RRM. The anchoring by U13 positions residues Ala147, Leu151, and Val154 of three consecutive helical turns ideally so they can interact with hydrophobic patches formed by residues of β2 and α1 of the RRM. This maintains the α3 position and stabilizes its helical structure. In the absence of this N-terminal anchoring, helix α3 explored other docking modes, for example by rotating so that Ala149 and Ala153 formed the RRM contacts, or by shifting register so that only Ala147 and Leu151 were docked, leaving Val154 exposed. In each case, some of the potential hydrophobic contacts were not realized in these alternative arrangements and helix α3 tended to locally uncoil in such segments. Thus, the MD simulations of the free protein revealed that α3 dynamically explores locally distorted conformations, while maintaining a loose and degenerate docking to the domain. This is consistent with the low order parameters indicated by NMR and the evidence that the helix maintains a partially folded state with occasional transient unfolding, as shown by the NMR relaxation dispersion experiments. A minor shift in the balance of forces provided by RNA binding is then enough to stabilize helix α3 conformation, to varying extents depending on the RNA sequence. This is achieved through the coupling between the specific network of interactions, which the RNA binding stabilizes in the region just N-terminal to α3, and the optimal docking mode imposed by the hydrophobic residues at the RRM-α3 interface. In this fashion, binding of SL UCUUU imposes a characteristic conformation of the β4/α3 loop (Figure 8A) which stabilizes α3. Such support is not provided by the SL UCGUU where G12 interferes with the formation of these stabilizing interactions (Figure 8B). It is likewise missing in the free PTB RRM1 where there is no RNA to facilitate these stabilizing interactions (Figure 8C). Given the large sensitivity of α3 towards even minor sequence alterations of the bound RNA, the potential energy surface corresponding to these transitions is likely very flat. We speculate that the μs dynamics of the protein-RNA interface that we see in the SL-UCUUU-RRM1 complex might originate from an exchange between conformations where U13 interacts with RRM1 and a minor state where U13 transiently does not interact with RRM1, which could be similar to the conformation seen for SL UCGUU.

### PTB function *in vivo* is sensitive to the disorder-order transition of the C-terminal helix of RRM1

**Figure 9.**
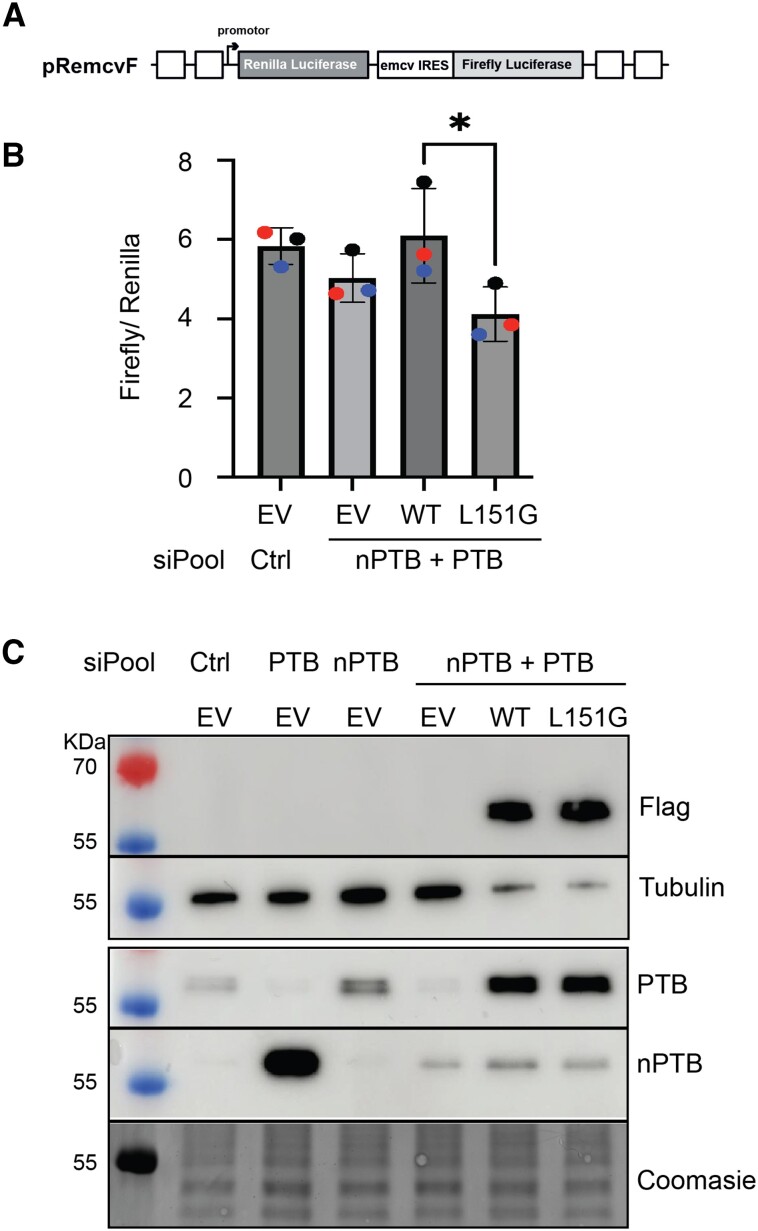
Luciferase assay measuring impact of WT and mutant PTB on IRES activity. (**A**) Hek293T cells were transfected with a dicistronic reporter plasmid pRemcvF along with cntrl siRNAs or siRNAs to knock down both PTB and nPTB. Expression of ectopic Flag-tagged wild type (WT) or mutant (L151G) PTB was achieved by transient transfections as indicated, or with an empty vector (EV) control. IRES activity was determined by measuring luminescence from firefly luciferase under the control of IRES and normalized to luminescence from Renilla luciferase to control for transfection efficiency. (**B**) Average IRES activity from three technical replicates for three independent transfections (black, red and blue) dots on the histogram are shown. Mutating L151 to G compromised the ability of PTB to activate IRES as compared to the WT protein (*P*-val 0.02 computed using a paired t-test). Results are means and error bars are standard deviation (SD) from three independent experiments. (**C**) Western blot analysis showing knock down of endogenous PTBP1 and nPTB along with ectopic expression of the respective Flag tagged proteins. Images of Tubulin and Coomasie stained membranes represent loading of total proteins probed with FLAG and PTB antibodies respectively.

To determine the importance of the disorder-to-order transition of the α3 helix of RRM1 for the functioning of PTB in a biological context, we assessed the impact of the L151G mutation in PTBs ability to enhance IRES translation. We performed IRES translation assays with HEK293T cells transfected with the dicistronic reporter plasmid pRemcvF and a second plasmid containing either an empty cassette, or expressing either wildtype PTB or a mutant PTB with the single amino acid substitution L151G (Figure 9A, B). Endogenous PTB and nPTB were knocked down using siRNA pools designed to target their respective 3′ UTRs as verified by western blots (Figure 9C). The mutant L151G PTB protein was compromised in its ability to activate the Luciferase reporter to levels similar to those observed with the WT protein (Figure 9B) thus demonstrating that the disorder-to-order transition of α3 is important for IRES function of PTB in cells.

## Discussion

### Role of dynamics in RNA binding and α3 helix stabilization

**Figure 10.**
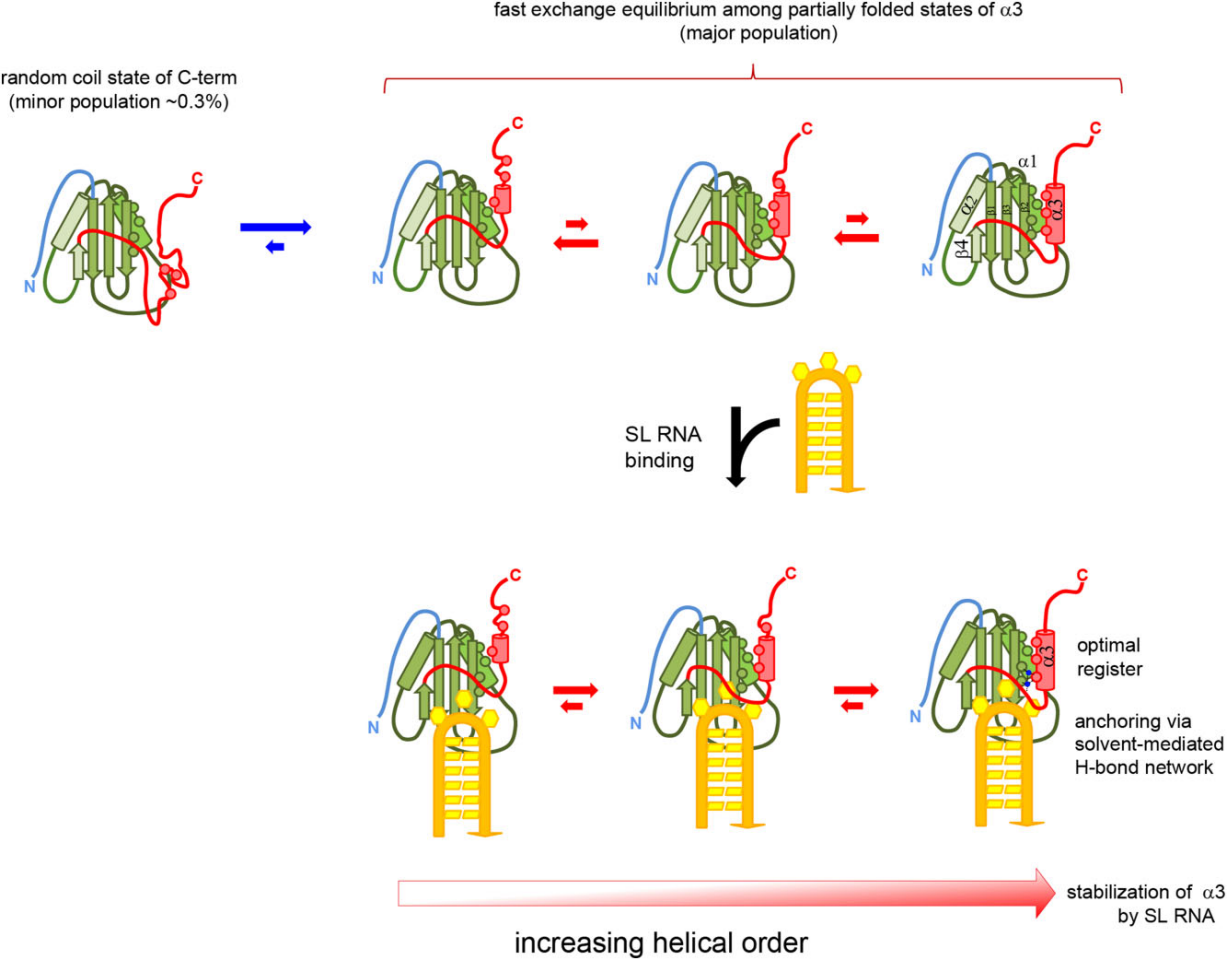
Schematic representation of the changes in dynamics and structure during the interaction of PTB RRM1 domain with stem–loop RNA. Secondary structural elements shown with lighter shades indicate regions with extensive dynamics. Slow exchange occurs between a minor population with the α3 region in an undocked, random coil state and a major population (ensemble) with a partially organized, docked α3 helix. SL RNA binding stabilizes the α3 helix conformation to different degrees; achieving maximum stability with SL UCUUU RNA whose apical loop interactions favorably position the N-terminus of α3 so that hydrophobic residues on successive helical turns have optimal register with hydrophobic residues of β2 and α1.

Binding of PTB RRM1 to SL UCUUU RNA is characterized by a major structural change namely, the folding of the C terminal residues 144−154 to form the α3 helix. Our dynamics study shows that this region exhibits some structural ordering in the free state. The presence of helical secondary structure in this region is supported by ^13^C secondary shifts (12) and characteristic medium range NOEs (Supplementary Figure S13). A periodic pattern of large *R*_ex_ is observed for the free protein in the C-terminal residues corresponding to successive turns of α3 facing the domain in the complex, and this is abolished by the mutation L151G which eliminates the helix (Figure 5). This mutation also abrogates exchange for residues in α1 and β2 that are interacting with α3 in the complex. These data suggest the presence of a partially ordered α3, which transiently unfolds and undocks from the domain in the free protein, as depicted schematically in Figure 10.

The detection of long-range NOEs between α3 and α1/β2 of the RRM are consistent with the presence of a significant population of conformations within the free protein's ensemble closely similar to the SL RNA-bound state as indicated by the calculation of the structure of the free protein (Figure 2B, D, Supplementary Figure S17 and Supplementary Figure S18, Supplementary Table S8). Upon binding to SL RNA, increased order is detected for residues 144−154, consistent with the stabilization of α3 (Figure 2). However, the order parameters and internal correlation times suggest that even in the complex, α3 is less rigid than the remaining secondary structures of RRM1. The extensive slow motion detected in the C-terminal residues of PTB RRM1 is quenched upon RNA binding with the exception of residues in the last turn of α3, indicating that there is still some mobility in this region in the complex. Together this data suggests that when SL RNA binds, the C-terminal region undergoes a population shift to an ensemble with more helical character (Figure 10). The differing degrees of helix formation observed in the C-terminus upon binding to SL RNA with different loop sequences (12) can be interpreted as a more or less restrictive selection of conformations with helical character among the ensemble. After an initial binding event, the contact with the RNA may induce additional adaptions causing a transition to a well-folded α3. As shown by the MD, the enhancement of α3-helical order upon SL UCUUU binding is facilitated by direct and water-mediated RNA contacts to the extended β4–α3 linker. These anchor the N-terminal end of α3 to the RRM, allowing the hydrophobic residues on one side of α3 to form interactions with hydrophobic residues of α1 and β2 (Figure 8). RRM1 can be regarded as a highly sensitive sensor, coupling the binding of SL RNA with the degree of ordering in a region not involved in the binding site and is reminiscent of other dynamically controlled allosteric proteins (70). Similar conclusions regarding the sensitivity of the α3 region to binding events at the β-sheet interface were also drawn from an MD study using the CHARMM36 force field with shorter 1us simulations of the free and bound states of PTB RRM1 and its L151G mutant, via perturbation response scanning (71).

### Dynamic allostery and its impact on PTB function

Much work has been devoted to understanding the role of individual domains of RNA-binding proteins in recognizing specific RNA targets. This divide-and-conquer approach has been very successful in revealing the underlying principles, as well as the diversity in strategies they use to recognize RNAs (72). However, multiple RRMs or other RNA binding motifs such as KH or Zinc fingers frequently occur in the same protein, and the coordination of these individual modules is an essential part of the function of multidomain RNA-binding proteins such as PTB (73). Attention has focused on the intervening sequences between the folded domains. These may in some cases act as passive linkers between the domains with highly disordered polymer-like conformations. However, there is evidence that these regions are functionally important (74–76). In the case of PTB and its alternatively spliced variants most of the sequence differences are located in the linkers, and the alternative spliced variants have altered activity (5). Recently, we have determined the conformation of full length PTB bound to an IRES using an integrated structural biology approach (11). This showed that both protein and RNA retain a significant degree of flexibility in the complex. None-the-less, PTB binding restricts the conformational space. In some of the conformations in this PTB/IRES ensemble, contacts occur between α3 from RRM1 and RRM2. Thus, α3 may also mediate domain-domain contacts to contribute to PTB function. Our IRES assay data with the L151G mutant clearly shows the significance of α3 in PTB function. In contrast to the previously investigated Δα3 truncation mutant (12), PTB[L151G] does not shorten the linker length between RRM1 and RRM2, and with the exception of L151, it retains all of the residues from the wildtype. Together with our dynamic study of PTB RRM1 and its L151G mutant, this furnishes a stringent test of the specific requirement of a disorder-to-order transition in the α3 region of PTB for IRES function, and clearly refutes a passive linker role.

It has been proposed that the function of multidomain proteins can be modulated by intervening disordered linkers through a cascade of conformational states they can acquire which help to preorganize the position of the domains (77). This requires that specific conformational states are encoded in the sequence of the linker region which steer towards productive conformations. We have shown that the 40-residue linker between RRM1 and RRM2 includes a highly conserved sequence, which is partially ordered in the free protein, but folds into a helix to varying degrees dependent on the sequence of the SL RNA binding at a remote site. Thus, PTB includes such a dynamically regulated allosteric linker which helps coordinate binding of RRM1 to an optimal SL with restriction of the conformational space of the neighboring RRM2. Dynamic allostery has the advantage of being responsive in a continuous way by integrating the inputs of multiple determinants of the order-disorder transition (78). In the case of PTB, the transition is influenced by the loop sequence of the bound SL RNA, but it may also be affected by other changes such as post-translational modification. Both the RRM1 β4−α3 and RRM1−RRM2 linkers are targets of a large number of post-translational modifications (79).

### Binding induced helix folding in C terminal regions of RRMs

Recently it was reported that the RRM domain of RBM20, a splicing regulator important for development in heart tissue also folds a C-terminal helix upon binding to a target single strand RNA and recognizes the sequence UCUU in a very similar fashion to PTB RRM1 (80). Although no C-terminal α-helix was detected in a structure determination of the free RBM20, similar to PTB RRM1, heteronuclear NOE data and secondary ^13^C shifts indicate the presence of a region with partial helical order at the C-terminus, which folds upon binding RNA. However, there are also significant differences between the α3 helices in these two proteins. RBM20 RRM α3 conformation is stabilized only by a specific single-stranded RNA sequence, with large decreases in affinity caused by nucleotide substitutions and no enhancement in α3 helix content observed. In contrast, PTB RRM1 is tolerant of pyrimidine to guanine substitutions at all loop positions with only 2-fold reduction in affinity, and merely a 4-fold reduction when binding a stem-loop incorporating the UUCG tetra-loop with inaccessible bases (12). This is probably because PTB RRM1 binding to stem–loop RNA is stabilized to a significant extent by non-specific contacts, which recognize RNA secondary structure. Unlike RBM20 RRM, PTB RRM1 transduces changes in base identity of the bound loop nucleotides into a change in the degree of α3 helical order.

### Correlation between dynamics changes and binding specificity

Although RNA binding reduces dynamics in most of the PTB RRM1 domain, increased slow timescale fluctuations are observed at the binding interface of the PTB RRM1/SL UCUUU complex. A similar phenomenon has been described in protein-DNA interactions with non-sequence-specific recognition of DNA (81). Two other RRM–RNA interactions whose dynamics changes on binding have been investigated in some detail are U1A RRM binding to polyadenylation inhibition element RNA (82), and CstF-64 RRM binding to downstream GU-rich sequence elements involved in regulation of mRNA polyadenylation (83). In both cases a C-terminal helix following the RRM occludes the RNA binding site and undergoes conformational changes upon RNA binding. Whereas U1A binds its cognate RNA specifically, resulting in a shift of the C-terminal helix to position it away from the RRM binding surface, CstF-64 RRM binds a range of GU-rich single strand RNAs, causing partial unfolding of the C-terminal helix. These differences are also reflected in dynamics changes. Whereas μs–ms exchange-induced line-broadening is quenched in regions of U1A making specific contacts to the RNA, for CstF-64, additional exchange appears on this timescale when the RNA is bound. Interestingly, for CstF-64 it was proposed that the RNA may also undergo additional dynamics upon binding although RNA dynamics was not measured, and this is indeed what we observe for the SL UCUUU. Generally, then for protein-RNA recognition, which involves specificity in some parts of the binding interface and non-specific interactions in other parts, increased mobility has been observed in those regions of the interface with more relaxed specificity requirements. A dynamic interface correlates with diffuse specificity, allowing a wide range of RNA sequences to be recognized. The slow exchange events observed at the PTB RRM1 binding interface in the complex, are consistent with the non-specific nature of RNA binding and the relatively similar binding affinities for SL RNAs containing a variety of pyrimidine-rich apical loop sequences (12). Among the RRMs of PTB whose dynamics has been investigated, the structurally linked RRM3 and RRM4 also show extensive μs−ms timescale motions throughout the domains in the free state as observed here for RRM1 (84). The inherent plasticity in the RRM domains and at the RNA binding interface may play a role in the ability of PTB to bind and remodel a diverse range of RNA substrates with complex topology. Examples include PTBs participation in regulation of splicing, where its N-terminal RRMs interact with SL4 of U1 snRNA (85), its role as a trans-acting factor of IRES, where the N-terminal RRMs make specific contacts to IRES domains and coordinate conformational changes important for IRES activation (86), and more recently described, its binding to long non-coding RNAs (87–89), which also exhibit complex secondary structure, to regulate cell differentiation (90).

### Impact of binding-induced dynamics changes on tertiary interactions

Our dynamics studies show that PTB RRM1 displays motions on different timescales involving a large fraction of the protein and that the C-terminal half shows dynamic features reminiscent of a partially folded protein. Upon binding to the stem-loop RNA, much of these dynamics are quenched, suggesting that interaction with the binding partner helps stabilize the RRM fold. In this respect it is similar to the Snu17p protein RRM, which together with polypeptide motifs in the proteins Bud13p and Pml1p forms the core of the retention and splicing complex: Snu17p RRM is highly dynamic as a monomer, with a particularly labile C-terminal half starting from α2 (91). Similar to PTB RRM1, a C-terminal helix forms upon binding Pml1p and leads to reduced dynamics in the domain. Both Snu17p and PTB participate in larger macromolecular complexes, which require the assembly of components in a coordinated fashion. This may be aided by dynamically controlled allosteric mechanisms involving the folding of additional secondary structure elements like the C-terminal helices of Snup17p or PTB RRM1. The picture obtained from the dynamics and MD studies of free and SL RNA-bound PTB RRM1 together with the observed systematic shift of amide signals at the α3/RRM interface (12), indicates that the degree of ordering is influenced by the identity of the loop nucleotides, and this in turn could influence the efficient assembly of productive complexes. Previously we showed that PTB enhancement of IRES mediated translation is reduced when stem-loop sequences inefficient at enhancing α3 helix formation are inserted into the IRES RNA (12). In addition, SL UCUUU binding quenches motions at the α1 and α2 helices. The surface formed by these two helices has been shown to be a binding site mediating protein-protein contacts in RRMs (7). The dynamic coupling of the RNA binding interface on one side of the RRM with this protein-interaction site on the other side may also control the recruitment of other binding partners in large macromolecular complexes. In the case of Snu17p, binding of Pml1p to the α1-α2 interface induces C-terminal helix formation and quenches motions in the RRM, resulting in enhanced RNA binding affinity. Together with our data, this observation suggests that a reciprocal relationship could operate in the case of PTB RRM1.

### Significance of a flexible RRM β4 strand

Another secondary structure element of PTB RRM1, which is dynamic in the free state, is β4. In the C-terminal RRM of SRSF1, an SR protein involved in regulation of alternative splicing, the β4 strand was shown to bind to a docking groove of the kinase SRPK1 preventing further phosphorylation of the immediately adjacent RS domain. The authors suggest that β4 of SRSF1 RRM2 may exist in equilibrium between folded and unfolded states, which would facilitate the binding of β4 to the docking site (92,93). Our dynamics data show that β4 of PTB RRM1 is a labile structural element visiting random-coil like conformations. Structural alignment of many RRMs shows that β4 is flanked by extended regions with no regular secondary structure (94) suggesting that it may show similar behavior in some of these RRMs, which could facilitate access to binding partners including modifying enzymes. In this context, it is interesting that the β4 strand of a number of RRMs including RRM4 of PTB and its paralogs was shown to contain a sequence which is a docking motif of protein phosphatase 1, and that the splicing activity of SRSF1 was impacted by mutating the docking motif in β4 of its RRM (95,96).

In conclusion, we have shown that the N-terminal RRM domain of PTB contains a highly dynamic C-terminal region including a partially folded C-terminal helix α3, whose rigidity is enhanced by SL RNA binding. Our MD simulations show how the degree of structural ordering in α3 is influenced by the apical loop sequence of the bound SL RNA. We thereby elucidate a mechanism to control the positioning of the N-terminal RRM domains of PTB by dynamic allostery involving the interdomain linker, the bound RNA, and solvent-mediated interactions at the protein-RNA interface. The significance of this dynamic allostery in PTB function is demonstrated by the reduced IRES activity of the single point mutant L151G. The dynamics observed in various regions of the RRM1 and the changes which occur on SL RNA binding are potential regulatory elements, which PTB may use to modulate its interaction with both protein and structured RNA binding partners, in its many roles in RNA biology.

## Supplementary Material

gkae713_Supplemental_File

## Data Availability

The consensus structure of PTB RRM1 and its resonance assignments have been deposited in the PDB and BMRB databases with accession codes 8BGF and 34766 respectively.
