# Supplementary material for: N-terminal domain of polypyrimidine-tract binding protein is a dynamic folding platform for adaptive RNA recognition
Source: Nucleic Acids Res. 2024 Aug 24;52(17):10683–704. doi: 10.1093/nar/gkae713 (PMC11417363; doi:10.1093/nar/gkae713)
Supplement: gkae713_Supplemental_File [file gkae713_supplemental_file.docx]

**Supporting Information**

**N-terminal domain of Polypyrimidine-tract binding protein is a dynamic folding platform for adaptive RNA recognition**

**Fred F. Damberger^§^, Miroslav Krepl^‖^, Rajika Arora^§^, Irene Beusch^§^, Christophe Maris^§,†^, Georg Dorn^§^, Jiří Šponer^‖^, Sapna Ravindranathan^‡,*^ and Frédéric H.-T. Allain^§,*^**

**^§^** Institute of Biochemistry, ETH Zurich, 8093 Zurich, Switzerland

**^‖^**Institute of Biophysics of the Czech Academy of Sciences, Kralovopolska 135, Brno 612 00, Czech Republic

**^‡^** Central NMR Facility, National Chemical Laboratory, Pune 411008, India.

[^*^damberger@bc.biol.ethz.ch](mailto:*damberger@bc.biol.ethz.ch)

^*^[allain@mol.biol.ethz.ch](mailto:allain@mol.biol.ethz.ch)

[^*^s.ravindranathan@ncl.res.in](mailto:*s.ravindranathan@ncl.res.in)

**Figure S1.** Relaxation rates of PTB RRM1 (open symbols) and PTB RRM1 when bound to SL UCUUU (filled symbols) measured at 313K on a 700 MHz spectrometer


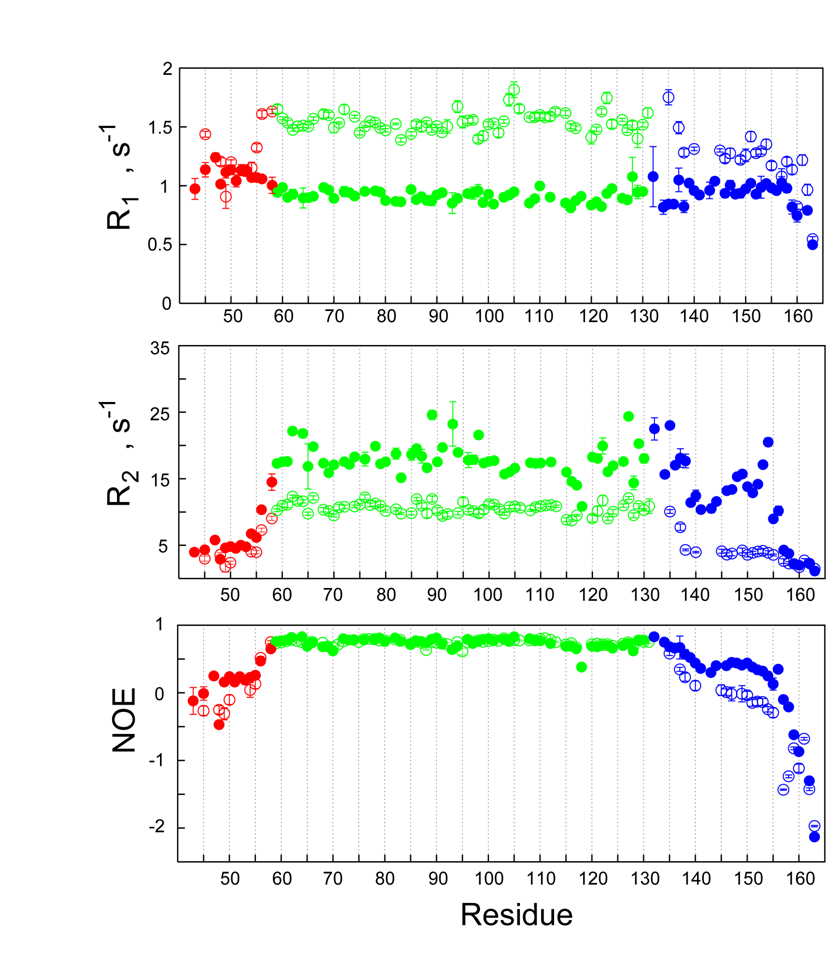


**Note S1. Reduced spectral density mapping of relaxation data**

^15^N T_1_, T_2_ and {^1^H}^15^N-NOE relaxation data were analyzed by the reduced spectral density mapping (RSDM) approach, which affords an overview of the motions occurring on a range of time scales below the correlation time of the molecule (1). The frequency spectrum of the motions of the N-H vector in amide groups contributing to the measured relaxation parameters are described by spectral density functions at five angular frequencies, J(0), J(ω_N_), J(ω_H_-ω_N_), J(ω_H_), J(ω_H_+ω_N_). In the RSDM approach, spectral densities J(0), J(ω_N_) and J(<ω_H_>) are calculated from the measured relaxation rates R_1_, R_2_ and NOE where it is assumed that J varies only very little from ω_H_-ω_N_ to ω_H_+ω_N_ so that the three spectral densities at higher frequencies can be approximated by the single value J(<ω_H_>) ~ J(0.87ω_H_) (2). In general, values of J(0) and J(ω_N_) decrease while values of J(<ω_H_>) increase with greater internal motion on ps time scales. However in the presence of slow motions (μs– ms timescales) J(0) values are significantly increased.

For RSDM analysis, the experimental relaxation rates R_1_, R_2_, and the heteronuclear cross relaxation rate, σ (derived from the NOE), were employed to calculate the spectral densities at the three frequencies, J(0), J(ω_N_) and J(<ω_H_>).

$$J\left( 0 \right)=\frac{3}{2\left( 3A+B \right)}\left[ -0.5R_{1}+0.5R_{2}-0.6\sigma\right] (S1a)$$

$$J\left( \omega_{N} \right)=\frac{1}{\left( 3A+B \right)}\left[ R_{1}-1.4\sigma\right] (S1b)$$

$$J\left( <\omega_{H}> \right)=\frac{\sigma}{5A} (S1c)$$

The constants are related to spin interaction constants by *A*=*d* ^2^/4 and *B*=*c*^2^, where *c* and *d* are the CSA and dipolar interaction constants defined above for η_xy_ measurements. Theoretical curves for the spectral densities were calculated assuming isotropic rotational diffusion of a rigid molecule,

$$J\left( \omega\right)=\frac{2}{5}\cdot\frac{\tau_{c}}{1+\left( \omega\tau_{c} \right)^{2}} (S2)$$

with correlation time τ_c_.

Figure S2 shows the J(0) versus J(ω_N_) correlation plots for PTB RRM1 in the free and SL UCUUU bound forms. For PTB RRM1 in the free state, the data points are partitioned into two distinct groups, with the residues belonging to the domain occurring at higher J(0) and J(ω_N_) values and the residues from the unstructured parts of the N and C termini at lower J(0) and J(ω_N_) values. This is typical for a protein which has reduced internal motions in the well-structured domain while undergoing increased ps motions in flexible regions. However, residues 145−155 are clustered slightly apart from the residues close to the N- and C-termini, suggesting that they are more constrained in their motions. Excluding points with J(0) values exceeding one standard deviation (SD) from the mean, a linear fit of J(0) versus J(ω_N_) intersects the parametric curve of J(0) versus J(ω_N_) calculated for isotropic rotation, at the point corresponding to a correlation time of 6.6 ns for the data points of the free protein. This represents an approximate estimate of the correlation time for global motion. Residues with elevated J(0) values are observed on the extreme right beyond the intersection of the dashed line and parametric curve, and this is indicative for the presence of slow exchange processes in the μs – ms timescales which enhances the R_2_ rate.

When PTB RRM1 binds SL UCUUU, the linear fit to the J(0) versus J(ω_N_) data, excluding residues with J(0) values exceeding one SD from the mean, gives an overall correlation time of 12.3 ns, reflecting a reduced rate of overall tumbling in the larger complex (Figure S2B). Interestingly, the slow exchange motion is not quenched on RNA binding, as several residues show increased J(0) values in the bound state as well. Moreover, many of the data points from the C-terminal residues 145−155 shift towards those from residues in the structured domain. This implies a significant decrease in flexibility of these residues on RNA binding. Their motional restriction in the bound state indicates structural ordering upon complex formation, consistent with the increased model-free order parameters (Figure 2), and the formation of the C-terminal helix α3

.

**Figure S2.** Reduced spectral density correlation plots of J(0) versus J(ω_N_) for (A) PTB RRM1 and (B) PTB RRM1 bound to SL UCUUU. Residues from the N-terminal tail, domain and C-terminal tail are shown in blue, green and red respectively. The grey curves represent the parametric plot of J(0) versus J(ω_N_) values calculated for different correlation times (τ_c_) by assuming isotropic rotational diffusion (eq. S2,). The dashed lines represent the linear fits to the J(0), J(ω_N_) data and the intercept on the parametric curve at the τ_c_ value, corresponding to the correlation time for overall motion, is indicated.


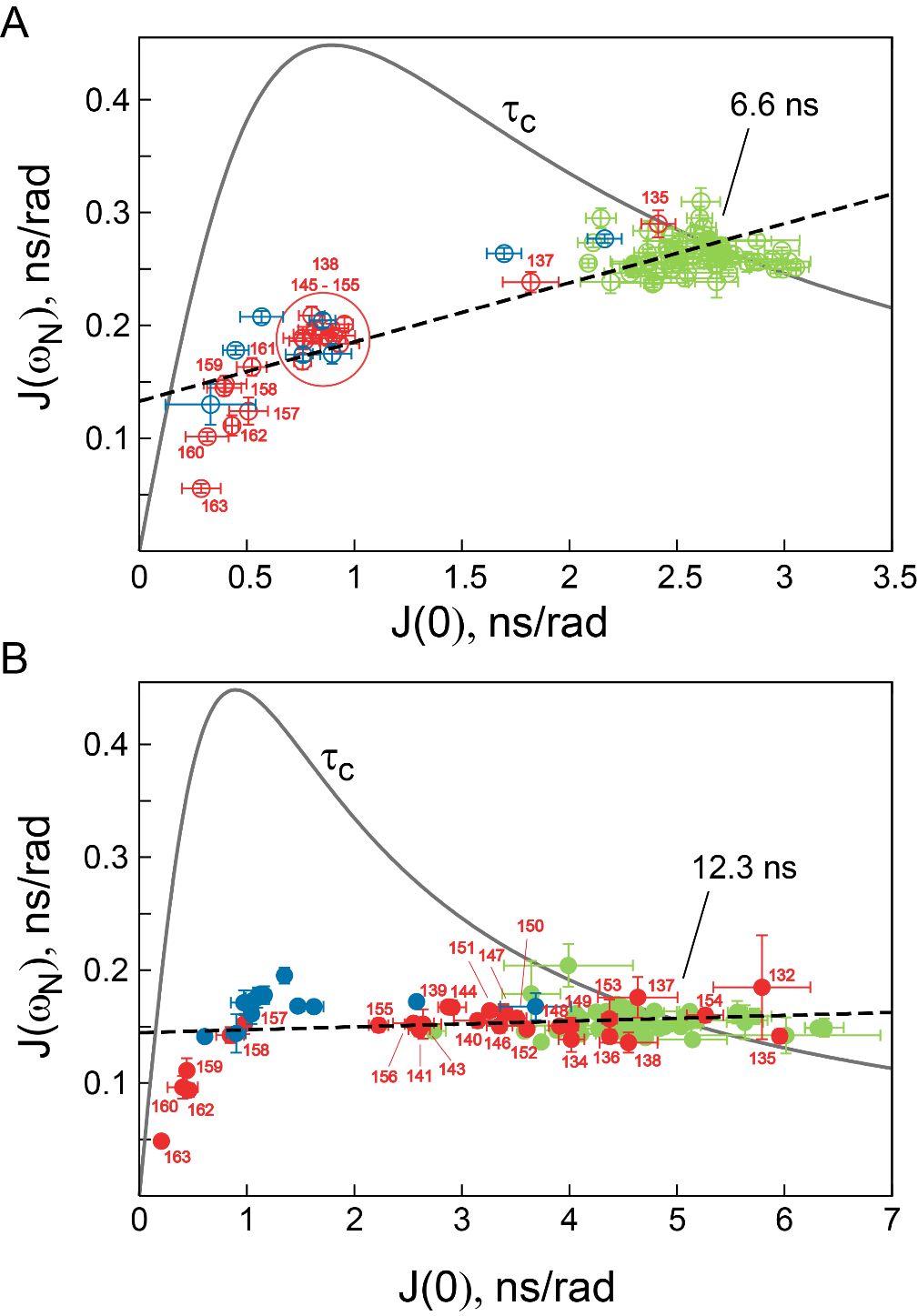


**Table S1.** Model-free internal motion parameters for PTB RRM1 at 313 K. PTB RRM1 was assumed to tumble isotropically in its free state since the use of an anisotropic rotational diffusion model did not lead to a statistically significant improvement in the fit to the experimental R_2_/R_1_ ratios. The correlation time estimated from R_2_/R_1_ ratios of residues not subject to slow conformational fluctuations and significant internal motions (3) is 6.54 ± 0.03ns, close to the estimate from reduced spectral density mapping (RSDM) analysis (Figure S2). “e” stands for error of the fitted parameter.

| Residue | ^*^S^2^=S_f_^2^S_s_^2^ | eS^2^ | τ_i_ , ps | eτ_i_ | R_ex_, s^-1^ | eR_ex_ | Model |
| --- | --- | --- | --- | --- | --- | --- | --- |
| K45 | 0.149 | 0.024 | 686.541 | 26.754 |  |  | 5 |
| K48 | 0.248 | 0.020 | 580.729 | 26.335 |  |  | 5 |
| G49 | 0.083 | 0.053 | 677.926 | 67.808 |  |  | 5 |
| D50 | 0.110 | 0.016 | 788.528 | 29.394 |  |  | 5 |
| A54 | 0.306 | 0.024 | 664.084 | 53.946 |  |  | 5 |
| G55 | 0.271 | 0.016 | 818.056 | 38.317 |  |  | 5 |
| V56 | 0.606 | 0.018 | 993.054 | 43.286 |  |  | 5 |
| S58 | 0.797 | 0.018 | 1634.060 | 212.151 |  |  | 5 |
| R59 | 0.919 | 0.014 | 854.834 | 208.191 |  |  | 2 |
| V60 | 0.957 | 0.007 | 283.056 | 103.112 | 0.665 | 0.154 | 4 |
| I61 | 0.945 | 0.008 | 88.787 | 21.904 | 0.926 | 0.241 | 4 |
| H62 | 0.913 | 0.005 | 34.804 | 7.423 | 2.509 | 0.134 | 4 |
| I63 | 0.928 | 0.005 | 58.058 | 9.892 | 1.693 | 0.288 | 4 |
| R64 | 0.927 | 0.006 | 77.480 | 11.555 | 1.622 | 0.135 | 4 |
| K65 | 0.920 | 0.005 | 72.928 | 9.926 |  |  | 2 |
| L66 | 0.959 | 0.004 | 221.152 | 98.012 | 1.796 | 0.179 | 4 |
| I68 | 0.935 | 0.014 | 645.459 | 192.266 |  |  | 2 |
| D69 | 0.905 | 0.014 | 611.903 | 109.543 |  |  | 2 |
| V70 | 0.885 | 0.009 | 96.101 | 12.909 |  |  | 2 |
| T71 | 0.934 | 0.004 | 137.193 | 16.447 |  |  | 2 |
| E72 | 0.905 | 0.020 | 1238.174 | 218.700 | 0.821 | 0.195 | 4 |
| E74 | 0.977 | 0.003 | 303.193 | 71.621 |  |  | 2 |
| V75 | 0.892 | 0.007 | 43.752 | 4.488 | 1.535 | 0.106 | 4 |
| I76 | 0.941 | 0.002 |  |  | 2.114 | 0.208 | 3 |
| S77 | 0.960 | 0.004 | 59.992 | 10.945 | 0.746 | 0.162 | 4 |
| L78 | 0.953 | 0.006 | 57.500 | 11.388 | 1.030 | 0.247 | 4 |
| G79 | 0.922 | 0.005 | 45.699 | 5.620 | 0.992 | 0.127 | 4 |
| L80 | 0.918 | 0.005 | 38.994 | 8.131 |  |  | 2 |
| F82 | 0.947 | 0.003 | 46.865 | 12.556 |  |  | 2 |
| G83 | 0.860 | 0.007 | 20.485 | 2.449 | 0.512 | 0.107 | 4 |
| V85 | 0.885 | 0.006 | 58.346 | 6.335 |  |  | 2 |
| T86 | 0.936 | 0.010 | 75.803 | 20.441 | 1.868 | 0.183 | 4 |
| N87 | 0.919 | 0.014 | 75.642 | 20.525 | 1.054 | 0.285 | 4 |
| L88 | 0.905 | 0.004 | 249.803 | 51.244 |  |  | 2 |
| L89 | 0.905 | 0.008 | 55.345 | 8.114 | 2.216 | 0.269 | 4 |
| M90 | 0.925 | 0.006 | 81.144 | 11.997 |  |  | 2 |
| L91 | 0.890 | 0.004 | 54.570 | 6.037 |  |  | 2 |
| K92 | 0.908 | 0.015 | 81.559 | 23.345 |  |  | 2 |
| K94 | 0.883 | 0.037 | 1309.561 | 686.110 |  |  | 2 |
| N95 | 0.892 | 0.025 | 306.087 | 167.881 | 1.813 | 0.431 | 4 |
| Q96 | 0.952 | 0.006 | 152.626 | 71.231 |  |  | 2 |
| A97 | 0.972 | 0.009 |  |  |  |  | 1 |
| F98 | 0.867 | 0.011 | 25.420 | 5.259 |  |  | 2 |
| I99 | 0.889 | 0.013 | 39.955 | 8.195 |  |  | 2 |
| E100 | 0.938 | 0.008 | 72.466 | 15.718 | 1.105 | 0.261 | 4 |
| M101 | 0.948 | 0.004 | 59.019 | 13.925 | 0.913 | 0.144 | 4 |
| N102 | 0.915 | 0.011 | 44.839 | 7.844 |  |  | 2 |
| T103 | 0.892 | 0.012 | 1669.933 | 352.484 |  |  | 5 |
| E104 | 0.861 | 0.026 | 2179.264 | 406.551 | 0.995 | 0.216 | 4 |
| E105 | 0.787 | 0.035 | 2715.531 | 517.689 | 1.474 | 0.28 | 4 |
| A106 | 0.928 | 0.016 | 1195.833 | 261.057 |  |  | 2 |
| N108 | 0.965 | 0.005 | 459.052 | 165.026 |  |  | 2 |
| T109 | 0.973 | 0.009 | 290.706 | 227.301 |  |  | 2 |
| M110 | 0.969 | 0.011 | 819.845 | 390.466 | 0.423 | 0.182 | 4 |
| V111 | 0.984 | 0.01 | 142.309 | 49.674 | 0.430 | 0.119 | 4 |
| N112 | 0.972 | 0.014 | 453.140 | 371.883 | 0.592 | 0.182 | 4 |
| Y113 | 0.911 | 0.014 | 1013.193 | 137.948 | 0.869 | 0.138 | 4 |
| T115 | 0.777 | 0.016 | 1347.391 | 122.353 |  |  | 5 |
| S116 | 0.779 | 0.025 | 1259.712 | 242.140 |  |  | 5 |
| V117 | 0.887 | 0.005 | 103.253 | 10.459 |  |  | 2 |
| V120 | 0.853 | 0.017 | 46.108 | 8.537 |  |  | 2 |
| L121 | 0.904 | 0.007 | 70.523 | 9.454 |  |  | 2 |
| R122 | 0.883 | 0.015 | 935.890 | 95.968 | 1.974 | 0.463 | 4 |
| G123 | 0.786 | 0.018 | 1541.872 | 122.656 |  |  | 2 |
| Q124 | 0.923 | 0.009 | 146.524 | 45.465 |  |  | 2 |
| I126 | 0.947 | 0.005 | 213.969 | 55.855 | 0.749 | 0.163 | 4 |
| Y127 | 0.903 | 0.007 | 55.752 | 8.309 | 2.377 | 0.148 | 4 |
| I128 | 0.904 | 0.010 | 113.749 | 26.840 |  |  | 2 |
| Q129 | 0.865 | 0.027 | 24.143 | 7.723 | 1.585 | 0.362 | 4 |
| F130 | 0.944 | 0.005 | 94.733 | 15.681 |  |  | 2 |
| S131 | 0.928 | 0.023 | 846.135 | 308.735 |  |  | 2 |
| E135 | 0.672 | 0.035 | 1137.009 | 88.815 | 2.007 | 0.303 | 4 |
| K137 | 0.711 | 0.036 | 503.765 | 163.835 |  |  | 2 |
| T138 | 0.316 | 0.011 | 847.699 | 39.277 |  |  | 5 |
| S140 | 0.273 | 0.008 | 791.973 | 34.822 |  |  | 5 |
| A145 | 0.292 | 0.018 | 719.188 | 39.896 |  |  | 5 |
| R146 | 0.244 | 0.018 | 734.596 | 28.462 |  |  | 5 |
| A147 | 0.259 | 0.021 | 710.891 | 46.084 |  |  | 5 |
| A149 | 0.317 | 0.023 | 636.882 | 51.100 |  |  | 5 |
| A150 | 0.240 | 0.022 | 713.633 | 32.944 |  |  | 5 |
| L151 | 0.249 | 0.016 | 666.825 | 21.372 |  |  | 5 |
| Q152 | 0.292 | 0.020 | 612.668 | 27.773 |  |  | 5 |
| A153 | 0.302 | 0.022 | 604.039 | 29.823 |  |  | 5 |
| V154 | 0.264 | 0.019 | 598.018 | 20.119 |  |  | 5 |
| N155 | 0.251 | 0.011 | 552.437 | 21.285 |  |  | 5 |
| V157 | 0.191 | 0.033 | 274.214 | 49.960 |  |  | 2 |
| Q158 | 0.128 | 0.018 | 380.243 | 13.935 |  |  | 2 |
| S159 | 0.106 | 0.024 | 492.342 | 20.520 |  |  | 5 |
| G160 | 0.091 | 0.023 | 400.473 | 28.144 |  |  | 5 |
| N161 | 0.152 | 0.019 | 505.475 | 15.615 |  |  | 5 |
| L162 | 0.137 | 0.010 | 302.282 | 10.402 |  |  | 5 |
| A163 | 0.107 | 0.002 | 75.908 | 2.264 |  |  | 2 |
| ^*^S^2^_s_ =1 for all models except 5. | | | | | | | |

**Table S2.** Model-free internal motion parameters for PTB RRM1-SL UCUUU at 313 K. A global correlation time of 11.79 ± 0.06ns and D_║_/D_┴_ ratio of 1.26 ± 0.08 was estimated for PTB RRM1 in the bound state by optimizing the axially symmetric diffusion tensor to fit experimental R_2_/R_1_ ratios. The atomic coordinates from the 3D structure of the PTB RRM1 SL UCUUU complex determined by NMR were employed in the calculation (PDB entry 2N3O).

| Residue | ^*^S^2^=S_f_^2^S_s_^2^ | eS^2^ | τ_i_ , ps | eτ_i_ | R_ex_,s^-1^ | eR_ex_ | Model |
| --- | --- | --- | --- | --- | --- | --- | --- |
| D43 | 0.167 | 0.034 | 725.897 | 59.165 |  |  | 5 |
| K45 | 0.176 | 0.023 | 800.462 | 37.762 |  |  | 5 |
| F47 | 0.258 | 0.010 | 976.706 | 13.048 |  |  | 5 |
| K48 | 0.101 | 0.008 | 612.038 | 2.857 |  |  | 5 |
| G49 | 0.187 | 0.016 | 921.211 | 33.047 |  |  | 5 |
| D50 | 0.202 | 0.009 | 990.014 | 12.716 |  |  | 5 |
| S51 | 0.181 | 0.016 | 917.964 | 33.168 |  |  | 5 |
| R52 | 0.211 | 0.014 | 984.669 | 18.774 |  |  | 5 |
| S53 | 0.205 | 0.017 | 936.699 | 29.753 |  |  | 5 |
| A54 | 0.323 | 0.011 | 883.524 | 19.852 |  |  | 5 |
| G55 | 0.279 | 0.006 | 950.086 | 18.428 |  |  | 5 |
| V56 | 0.546 | 0.005 | 962.970 | 14.621 |  |  | 5 |
| S58 | 0.761 | 0.034 | 1025.888 | 154.846 |  |  | 5 |
| R59 | 0.953 | 0.008 | 74.750 | 20.217 |  |  | 2 |
| V60 | 0.947 | 0.008 | 870.801 | 143.787 |  |  | 5 |
| I61 | 0.909 | 0.014 | 26.014 | 6.393 | 1.207 | 0.303 | 4 |
| ^**^H62 | 1 |  |  |  | 4.342 | 0.359 | 3 |
| ^**^R64 | 1 |  |  |  | 3.244 | 0.361 | 3 |
| K65 | 0.948 | 0.015 | 141.229 | 19.601 |  |  | 2 |
| L66 | 0.931 | 0.014 | 58.143 | 16.954 | 2.642 | 0.400 | 4 |
| I68 | 0.949 | 0.003 | 219.763 | 45.555 | 0.399 | 0.081 | 4 |
| D69 | 0.889 | 0.004 | 491.705 | 56.832 |  |  | 5 |
| V70 | 0.884 | 0.005 | 396.405 | 44.760 |  |  | 5 |
| E72 | 0.984 | 0.006 | 134.187 | 16.727 |  |  | 2 |
| G73 | 0.954 | 0.008 | 54.543 | 17.220 |  |  | 2 |
| E74 | 0.977 | 0.004 | 124.823 | 38.648 |  |  | 2 |
| I76 | 0.958 | 0.011 |  |  |  |  | 1 |
| L78 | 0.971 | 0.004 | 987.824 | 136.698 | 1.390 | 0.282 | 4 |
| G79 | 0.963 | 0.006 | 35.436 | 9.353 |  |  | 2 |
| L80 | 0.865 | 0.009 | 22.234 | 3.116 | 2.144 | 0.360 | 4 |
| F82 | 0.894 | 0.018 | 11.428 | 1.443 | 2.422 | 0.489 | 4 |
| G83 | 0.864 | 0.005 | 21.894 | 1.635 |  |  | 2 |
| V85 | 0.948 | 0.007 | 669.051 | 102.150 | 0.675 | 0.517 | 4 |
| T86 | 0.920 | 0.011 | 33.692 | 6.948 | 2.368 | 0.228 | 4 |
| N87 | 0.901 | 0.014 | 31.605 | 5.486 | 2.302 | 0.640 | 4 |
| L88 | 0.904 | 0.012 | 40.357 | 6.458 |  |  | 2 |
| L89 | 0.886 | 0.025 |  |  | 8.816 | 0.598 | 3 |
| M90 | 0.957 | 0.010 |  |  |  |  | 1 |
| L91 | 0.948 | 0.013 | 105.461 | 63.115 | 2.383 | 0.286 | 4 |
| G93 | 0.854 | 0.052 | 50.215 | 37.101 | 7.374 | 2.121 | 4 |
| K94 | 0.937 | 0.014 | 102.842 | 48.260 | 0.965 | 0.322 | 4 |
| Q96 | 0.943 | 0.014 | 35.235 | 12.122 |  |  | 2 |
| A97 | 0.964 | 0.010 | 170.478 | 54.678 |  |  | 2 |
| F98 | 0.956 | 0.023 | 81.695 | 18.661 | 4.423 | 0.453 | 4 |
| I99 | 0.865 | 0.018 | 13.946 | 5.575 | 1.781 | 0.560 | 4 |
| E100 | 0.960 | 0.009 | 41.539 | 16.802 |  |  | 2 |
| M101 | 0.930 | 0.009 | 37.469 | 7.851 |  |  | 2 |
| T103 | 0.879 | 0.004 | 16.174 | 1.782 |  |  | 2 |
| E104 | 0.901 | 0.007 | 31.892 | 3.404 |  |  | 2 |
| E105 | 0.931 | 0.008 |  |  |  |  | 1 |
| N108 | 0.865 | 0.014 | 11.299 | 1.911 | 1.812 | 0.256 | 4 |
| T109 | 0.894 | 0.012 | 25.560 | 3.693 | 1.208 | 0.237 | 4 |
| M110 | 0.973 | 0.006 | 463.328 | 139.181 |  |  | 2 |
| N112 | 0.896 | 0.008 | 41.124 | 4.185 | 1.433 | 0.207 | 4 |
| T115 | 0.864 | 0.012 | 39.982 | 4.757 |  |  | 2 |
| S116 | 0.787 | 0.004 | 23.371 | 1.916 |  |  | 2 |
| V117 | 0.781 | 0.007 | 544.893 | 71.465 |  |  | 5 |
| T118 | 0.571 | 0.011 | 721.132 | 22.937 |  |  | 5 |
| V120 | 0.895 | 0.007 | 52.028 | 4.217 | 0.741 | 0.204 | 4 |
| L121 | 0.924 | 0.008 | 85.140 | 12.985 |  |  | 2 |
| R122 | 0.845 | 0.013 | 32.597 | 4.043 | 3.928 | 0.732 | 4 |
| G123 | 0.902 | 0.007 | 57.979 | 5.679 |  |  | 2 |
| Q124 | 0.942 | 0.005 | 198.071 | 66.419 |  |  | 2 |
| I126 | 0.925 | 0.007 | 75.926 | 11.605 |  |  | 2 |
| Y127 | 0.929 | 0.040 | 60.751 | 5.958 | 6.668 | 0.738 | 4 |
| I128 | 0.832 | 0.032 | 48.857 | 14.135 |  |  | 2 |
| ^**^Q129 | 1 |  |  |  | 2.500 | 0.350 | 3 |
| F130 | 0.964 | 0.010 | 74.811 | 37.591 |  |  | 2 |
| ^**^N132 | 1 |  |  |  | 4.551 | 0.978 | 3 |
| K134 | 0.839 | 0.014 | 19.501 | 5.282 |  |  | 2 |
| E135 | 0.851 | 0.019 | 38.567 | 6.634 | 7.279 | 0.385 | 4 |
| L136 | 0.870 | 0.007 | 48.595 | 3.452 |  |  | 2 |
| K137 | 1 |  |  |  |  |  | global |
| T138 | 0.778 | 0.028 | 42.181 | 8.888 | 3.787 | 0.783 | 4 |
| D139 | 0.615 | 0.007 | 934.812 | 19.407 |  |  | 5 |
| S140 | 0.608 | 0.020 | 837.201 | 45.200 |  |  | 5 |
| S141 | 0.535 | 0.012 | 761.762 | 30.861 |  |  | 5 |
| N143 | 0.512 | 0.022 | 772.928 | 47.565 |  |  | 5 |
| Q144 | 0.560 | 0.014 | 884.120 | 25.033 |  |  | 5 |
| R146 | 0.660 | 0.013 | 691.536 | 32.364 |  |  | 5 |
| A147 | 0.650 | 0.011 | 840.924 | 36.905 |  |  | 5 |
| Q148 | 0.793 | 0.009 | 492.790 | 38.923 |  |  | 5 |
| A149 | 0.842 | 0.008 | 122.194 | 18.974 |  |  | 2 |
| A150 | 0.670 | 0.010 | 763.328 | 30.949 |  |  | 5 |
| L151 | 0.633 | 0.006 | 777.094 | 16.893 |  |  | 5 |
| Q152 | 0.738 | 0.006 | 475.460 | 20.142 |  |  | 5 |
| A153 | 0.812 | 0.035 | 455.281 | 36.609 | 1.071 | 0.604 | 4 |
| V154 | 0.763 | 0.017 | 515.647 | 34.131 | 5.077 | 0.469 | 4 |
| N155 | 0.466 | 0.015 | 654.317 | 40.573 |  |  | 5 |
| S156 | 0.535 | 0.016 | 760.820 | 33.127 |  |  | 5 |
| V157 | 0.183 | 0.016 | 732.888 | 16.912 |  |  | 5 |
| Q158 | 0.145 | 0.016 | 696.176 | 12.503 |  |  | 5 |
| S159 | 0.071 | 0.020 | 568.222 | 7.676 |  |  | 5 |
| G160 | 0.066 | 0.023 | 495.551 | 11.926 |  |  | 5 |
| L162 | 0.083 | 0.014 | 387.628 | 4.288 |  |  | 5 |
| A163 | 0.033 | 0.007 | 257.957 | 5.688 |  |  | 5 |
| ^*^S^2^_s_ =1 for all models except 5.  ^**^Data for these residues gave the best fit using a global motion with correlation time τ_c_ and a R_ex_ term to account for enhanced R_2_ values. | | | | | | | |

**Figure S3.** ^15^N CPMG dispersion data of PTB RRM1 domain residues at 313K. (A) ΔR_2_^eff^ = R_2_^eff^(υ_cp_=25Hz) − R_2_eff(υ_cp_=750Hz) measured at 750MHz field, plotted versus sequence for all domain residues. (B) Fits of the Fast exchange equation (see footnote, Table S4) to ^15^N CPMG dispersion data obtained at 900 MHz (triangle) and 750 MHz (circle). (C) Fits of the General Exchange equation (eq. 2, main text) to ^15^N CPMG dispersion data obtained at 900 MHz (triangle) and 750 MHz (circle). Obtained fitting parameters for B and C are listed in tables S3 and S4 respectively.


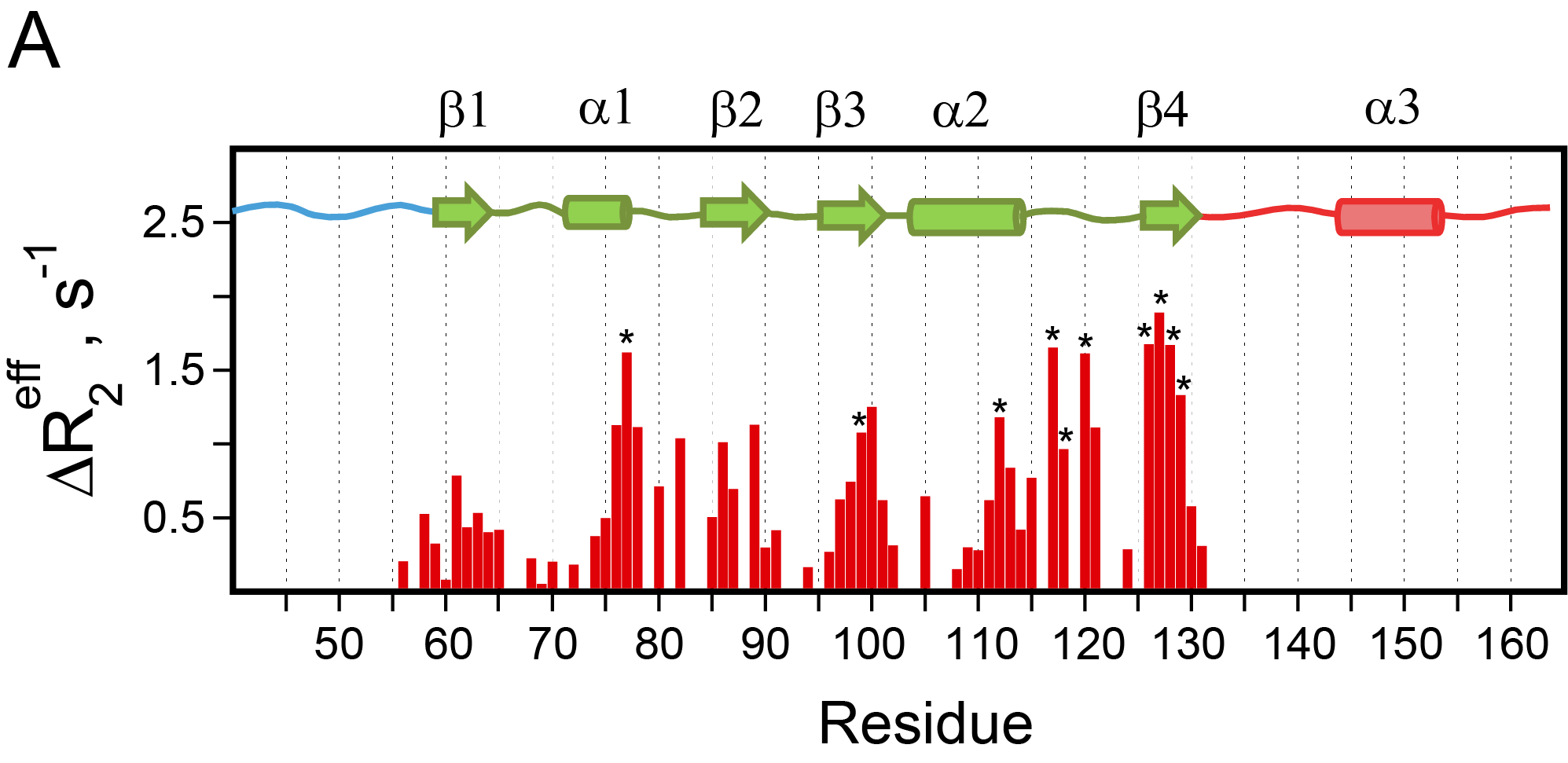


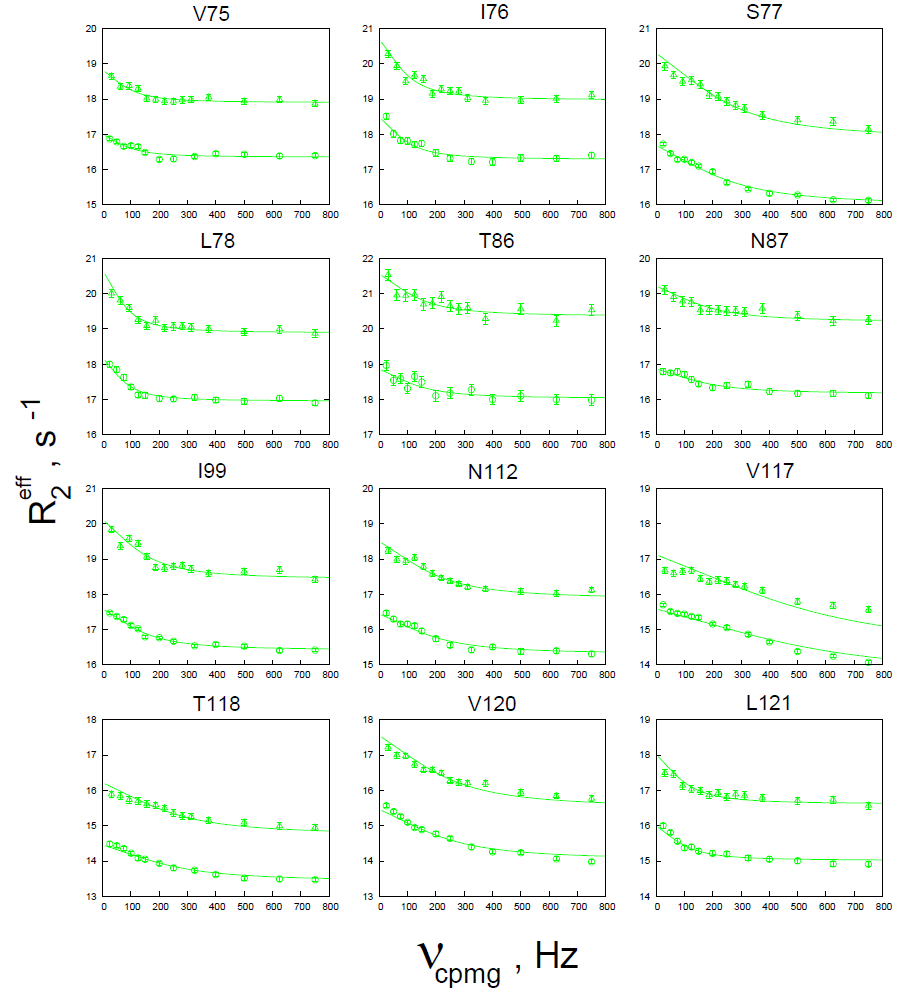
B

C

| **Table S3.** Two site Fast Exchange parameters from fits to CPMG dispersion data of domain residues of PTB RRM1 at 313K. | | | | | |
| --- | --- | --- | --- | --- | --- |
| Residue | R_2_^0^(750), s^-1^ | R_2_^0^(900), s^-1^ | φ_ex_^CP^=p_a_p_b_Δω^2^ | k_ex_, s^-1^ | Reduced χ^2^ |
| V75 | 16.36 ± 0.03 | 17.90 ± 0.04 | 377 ± 55 | 584 ± 108 | 1.20 |
| I76 | 17.29 ± 0.04 | 18.97 ± 0.05 | 730 ± 86 | 607 ± 89 | 1.61 |
| S77 | 16.00 ± 0.06 | 17.88 ± 0.09 | 2646 ± 265 | 1570 ± 134 | 1.83 |
| L78 | 16.96 ± 0.03 | 18.89 ± 0.04 | 626 ± 52 | 505 ± 57 | 0.89 |
| T86 | 18.03 ± 0.06 | 20.36 ± 0.08 | 730 ± 165 | 876 ± 213 | 0.95 |
| N87 | 16.87 ± 0.04 | 18.21 ± 0.06 | 787 ± 136 | 1120 ± 188 | 0.72 |
| I99 | 16.42 ± 0.04 | 18.43 ± 0.06 | 1072 ± 109 | 914 ± 97 | 1.92 |
| N112 | 15.31 ± 0.05 | 16.88 ± 0.06 | 1395 ± 162 | 1232 ± 134 | 1.40 |
| V117 | 13.69 ± 0.33 | 14.40 ± 0.49 | 6434 ± 2695 | 3395 ± 892 | 5.56^a^ |
| T118 | 13.44 ± 0.05 | 14.75 ± 0.07 | 1559 ± 209 | 1524 ± 176 | 1.21 |
| V120 | 14.05 ± 0.07 | 15.52 ± 0.10 | 2097 ± 289 | 1478 ± 178 | 2.93^a^ |
| L121 | 15.02 ± 0.03 | 16.62 ± 0.05 | 640 ± 77 | 655 ± 94 | 1.37 |
| I126 | 16.91 ± 0.11 | 18.63 ± 0.17 | 3153 ± 600 | 1931 ± 289 | 4.06^a^ |
| Y127 | 17.48 ± 0.16 | 19.25 ± 0.24 | 4031 ± 977 | 2280 ± 407 | 2.27^a^ |
| I128 | 15.31 ± 0.18 | 16.29 ± 0.26 | 3446 ± 1109 | 2405 ± 557 | 2.63^a^ |
| Q129 | 16.13 ± 0.06 | 17.68 ± 0.08 | 1439 ± 213 | 1253 ± 172 | 1.87 |
| Data were fitted to the fast exchange equation (4):  $R_{2}^{eff}(\nu_{CP})=R_{2}^{0}+\frac{\Phi_{ex}^{CP}}{k_{ex}}\left\{ 1-\frac{2tanh\left[ k_{ex}/2\nu_{CP} \right]}{k_{ex}/\nu_{CP}} \right\}$  Errors refer to the uncertainty in the fit parameters.  ^a^ Several residues which show strong dispersion are poorly fit by fast exchange model. | | | | | |

| **Table S4.** Two site General Exchange parameters from individual fits to CPMG dispersion data of domain residues of PTB RRM1 at 313K and comparison to global fits^§^ | | | | | | |
| --- | --- | --- | --- | --- | --- | --- |
| Residue | R_2_^0^(750), s^-1^ | R_2_^0^(900), s^-1^ | Δω, ppm | p_b_, % | k_ex_, s^-1^ | Reduced χ^2^ |
| S77 | 16.02 ± 0.04 | 18.00 ± 0.07 | 1.90 ± 0.20 | 0.36 ± 0.05 | 1008 ± 171 | 1.15 |
| I99 | 16.43 ± 0.04 | 18.50 ± 0.06 | 1.25 ± 0.21 | 0.39 ± 0.03 | 522 ± 188 | 1.41 |
| N112 | 15.29 ± 0.03 | 16.95 ± 0.04 | 1.73 ± 0.13 | 0.29 ± 0.02 | 587 ± 142 | 0.74 |
| V117 | 13.90 ± 0.10 | 14.95 ± 0.15 | 3.91 ± 0.28 | 0.19 ± 0.04 | 1249 ± 348 | 1.83 |
| T118 | 13.44 ± 0.03 | 14.85 ± 0.05 | 1.93 ± 0.17 | 0.22 ± 0.02 | 777 ± 170 | 0.62 |
| V120 | 14.05 ± 0.05 | 15.62 ± 0.08 | 1.77 ± 0.25 | 0.34 ± 0.06 | 897 ± 225 | 2.11 |
| I126^ǂ^ | 16.93 ± 0.06 | 18.84 ± 0.10 | 2.50 ± 0.20 | 0.28 ± 0.02 | 905 ± 236 | 2.07 |
| Y127^ǂ^ | 17.54 ± 0.07 | 19.57 ± 0.10 | 2.77 ± 0.19 | 0.30 ± 0.03 | 783 ± 233 | 1.03 |
| I128^ǂ^ | 15.33 ± 0.10 | 16.50 ± 0.14 | 3.07 ± 0.34 | 0.21 ± 0.02 | 1017 ± 410 | 1.67 |
| Q129^ǂ^ | 16.12 ± 0.04 | 17.76 ± 0.06 | 1.68 ± 0.20 | 0.30 ± 0.03 | 630 ± 207 | 1.27 |
| Global fit of General Exchange equation to data from β4 strand residues (see Figure 3, main text) | | | | | | |
| I126 | 16.93 ± 0.05 | 18.83 ± 0.07 | 2.59 ± 0.12 | 0.27 ± 0.01^*^ | 892 ± 53^*^ | 2.07 |
| Y127 | 17.53 ± 0.09 | 19.54 ± 0.10 | 2.90 ± 0.17 |  |  | 1.13 |
| I128 | 15.44 ± 0.07 | 16.63 ± 0.08 | 2.23 ± 0.15 |  |  | 2.17 |
| Q129 | 16.08 ± 0.05 | 17.67 ± 0.06 | 1.80 ± 0.08 |  |  | 1.52 |
| Errors refer to the uncertainty in the fit parameters.  ^§^At 313K, NMR samples of PTB RRM1 in the free state were not sufficiently stable to allow CPMG measurements of long durations at the different irradiation offsets required to obtain dispersion curves free from offset related distortions. Therefore, only data from residues which show relatively strong dispersion profiles and a maximal offset of ±2.0 ppm from the CPMG irradiation frequency were analyzed.  ^ǂ^The β4 strand residues do not show significant chemical shift changes when the L151G mutation is introduced and R_ex_ is of similar magnitude as in the wild type (Figure 5A). The small size of these chemical shift changes (due to mutation) relative to Δω obtained from fitting the CPMG curves rule out exchange due to unfolding of a partially formed α3 as the source of msec timescale motions causing exchange in β4.  ^*^Common parameters in global fit to dispersion data from β4 residues. | | | | | | |

**Figure S4.** Secondary structure prediction of α-helix and β-strand based on backbone chemical shifts of PTB RRM1 (A) and PTB RRM1-SL UCUUU (B) using the program δ2D (5). The probabilities for 3^10^-helical and poly-proline II coil conformations are omitted for clarity.

**
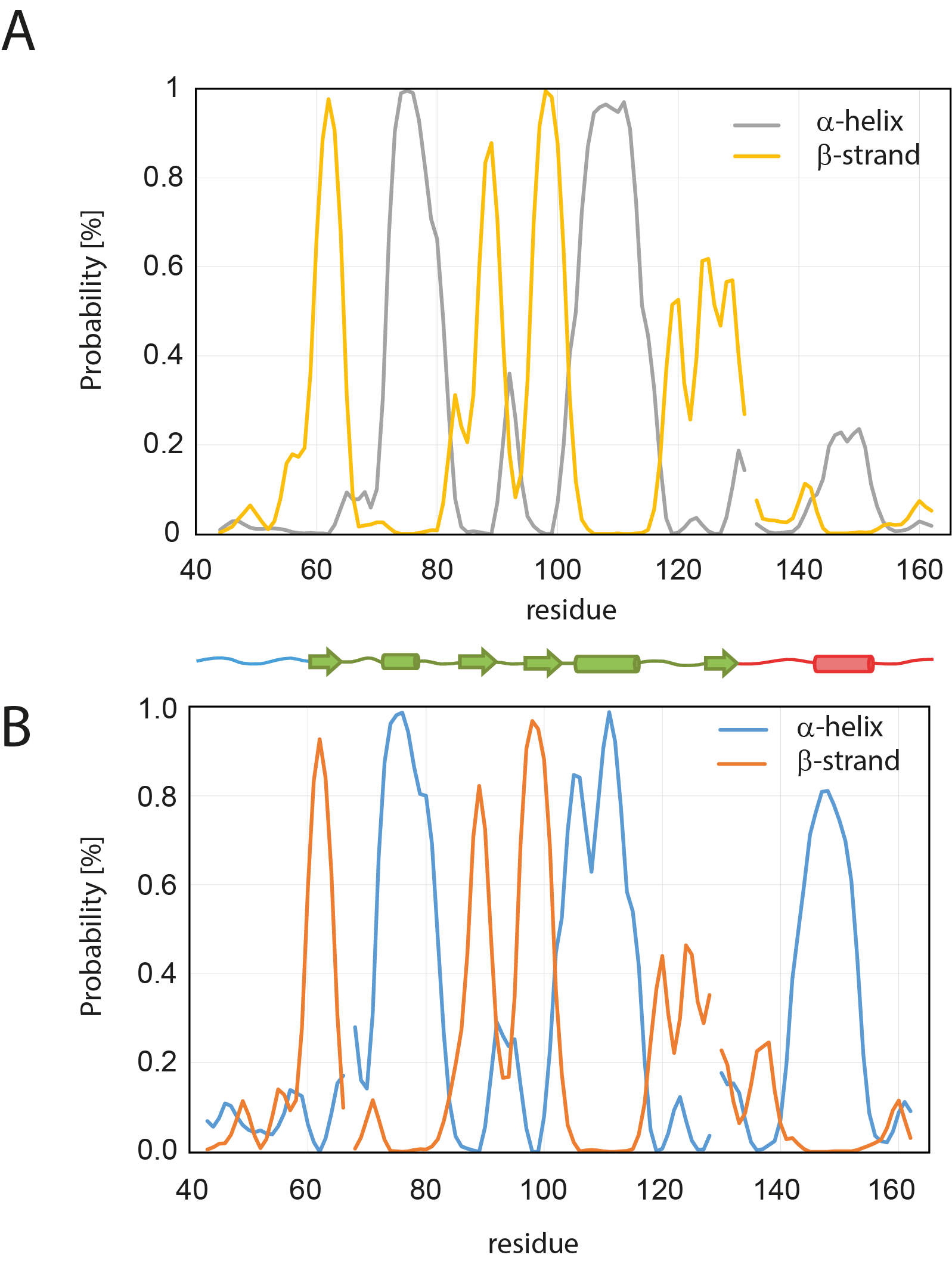
**

**Figure S5.** ^15^N R_1ρ_ dispersion data of PTB RRM1 SL-UCUUU at 313K. (A) ΔR_2_^eff^ = R_2_^eff^(ω^2^_eff_=low) − R_2_^eff^(ω^2^_eff_=high) plotted versus sequence. (B) Individual fits of two site Fast Exchange model to ^15^N R_1ρ_ dispersion data of PTB RRM1 SL-UCUUU at 313K.


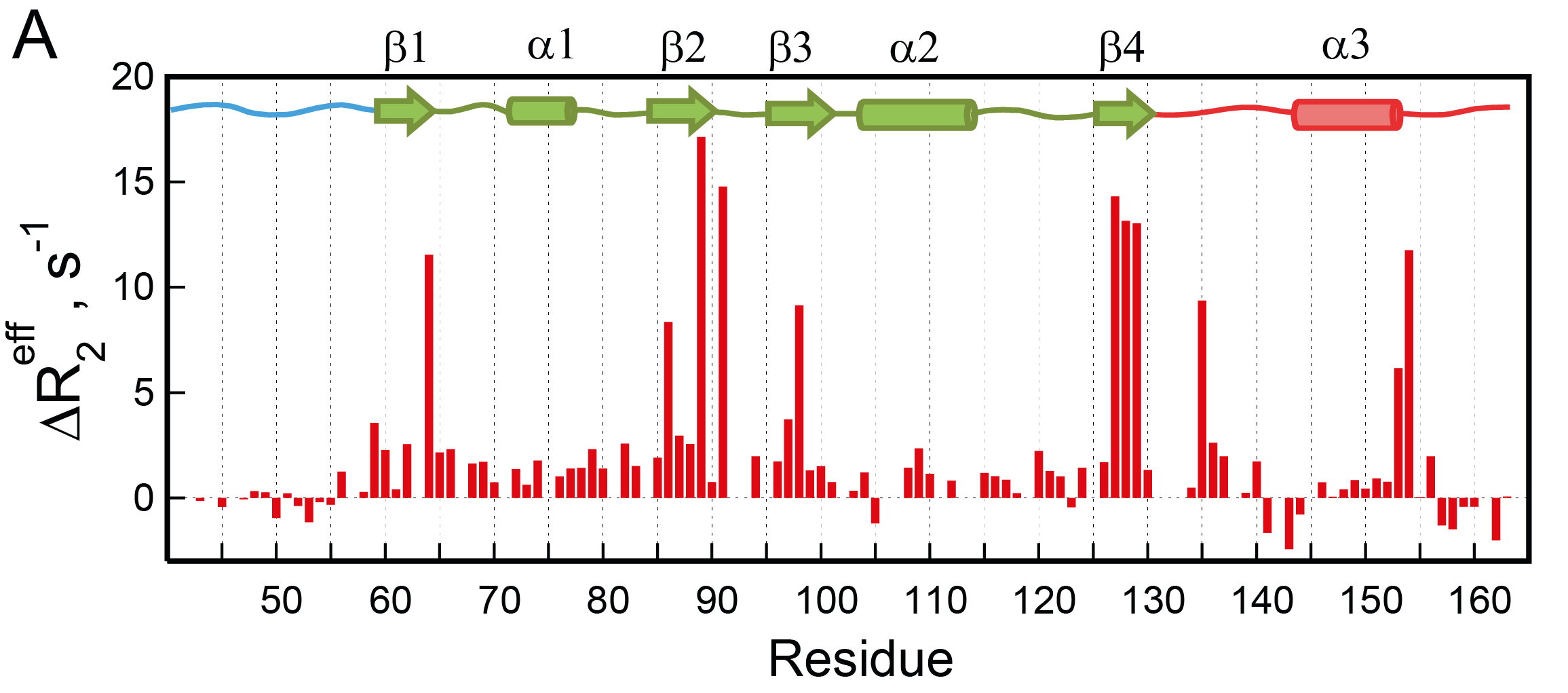


B

**Figure S6.** Comparison of R_2_^eff^ rates measured at two effective field values for SL UCUUU in the free (A) and PTB RRM1 bound states (B). Colored and black bars correspond to ω^2^_eff_ = ω^2^_SL_ + ω^2^_off_ values of ~1×10^8^ and ~14×10^8^ (rad^/^s)^2^ respectively.

**Figure S7.** ^13^C R_1ρ_ dispersion data for loop nucleotides. (A) C1′ carbons in SL UCUUU in the free state, (B) C6 carbons in SL UCUUU in the free state, and, (C) C6 carbons in SL UCUUU in the bound state.

**Figure S8.** Individual fits of two site Fast Exchange model to ^13^C R_1ρ_ dispersion data for C1′ signals of SL UCUUU bound to PTB RRM1 at 313K.

| **Table S5.** Two site Fast Exchange parameters from fits to R_1ρ_ dispersion data from PTB RRM1 (^15^N) and SL UCUUU (^13^C) in the bound state at 313K. | | | | | | | |
| --- | --- | --- | --- | --- | --- | --- | --- |
| Residue | R_1_,s^-1 *^ | R_2_^0^, s^-1**^ | φ^SL^_ex_,  10^4^ s^-1 a^ | k_ex_, 10^3^ s^-1 a^ | Reduced χ^2^ | R_2_^0^, s^-1 **^ | φ^SL^_ex_,  10^4^ s^-1 b^ |
| R64 | 0.90±0.09 | 17.45± 1.11 | 11.46 ± 0.27 | 6.27 ± 0.84 | 0.32 | 17.45± 1.11 | 11.56 ± 0.26 |
| T86 | 0.88±0.02 | 16.67± 0.58 | 8.97 ± 0.38 | 6.83 ± 1.08 | 1.47 | 16.67± 0.58 | 8.96 ± 0.36 |
| L89 | 0.87±0.04 | 18.06± 1.94 | 17.11 ± 1.00 | 6.61 ± 1.45 | 1.13 | 18.06± 1.94 | 17.14 ± 0.94 |
| L91 | 0.94±0.03 | 14.73± 0.29 | 13.65 ± 0.49 | 7.65 ± 0.80 | 1.59 | 14.73± 0.29 | 13.56 ± 0.47 |
| F98 | 0.96±0.05 | 14.64± 0.43 | 22.29 ± 2.40 | 7.02 ± 1.93 | 0.49 | 14.64± 0.43 | 22.41 ± 0.77 |
| Y127 | 0.88±0.02 | 16.65± 0.90 | 18.09 ± 1.29 | 7.14 ± 2.05 | 1.25 | 16.65± 0.90 | 18.18 ± 0.99 |
| I128 | 1.08±0.16 | 14.58± 0.57 | 13.14 ± 0.45 | 6.81 ± 1.06 | 0.91 | 14.58± 0.57 | 13.14 ± 0.43 |
| Q129 | 0.95±0.06 | 15.70± 0.42 | 13.05 ± 0.78 | 8.90 ± 1.43 | 1.49 | 15.70± 0.42 | 12.43 ± 0.51 |
| E135 | 0.84±0.03 | 16.69± 0.83 | 14.96 ± 1.48 | 11.05 ± 3.20 | 5.31 | 16.69± 0.83 |  |
| A153 | 0.99±0.09 | 11.44± 0.32 | 15.08 ± 0.78 | 10.87 ± 1.12 | 2.99 | 11.44± 0.32 |  |
| V154 | 1.02±0.03 | 10.38± 0.15 | 32.39 ± 5.70 | 5.30 ± 1.58 | 1.61 | 10.38± 0.15 | 28.41 ± 0.64 |
|  |  |  |  |  |  |  |  |
| Nucleotide | R_1_,s^-1 *^ | R_2_^0^, s^-1 a^ | φ^SL^_ex_,  10^4^ s^-1 a^ | k_ex_, 10^3^ s^-1 a^ | Reduced χ^2^ | R_2_^0^, s^-1 b^ | φ^SL^_ex_,  10^4^ s^-1 b^ |
| U12 | 1.71± 0.03 | 47.24 ± 0.47 | 7.05 ± 0.71 | 5.63 ± 0.51 | 1.1 | 46.42 ± 0.41 | 9.14 ± 0.46 |
| U13 | 2.23± 0.02 | 35.81 ± 0.24 | 7.18 ± 0.45 | 6.45 ± 0.43 | 1.12 | 35.60 ± 0.16 | 7.63 ± 0.21 |
| U14 | 2.27± 0.03 | 35.74 ± 0.39 | 15.57 ± 0.75 | 7.52 ± 0.34 | 0.54 | 36.26 ± 0.28 | 14.39 ± 0.35 |
| Errors refer to the uncertainty in the fit parameters.  ^*^Experimental R_1_  ^**^ estimated from κη_xy_  ^a^ individual fits  ^b^ global fit with k_ex_ = 6.92 ± 0.23 10^3^ s^-1^ (E135 and A153 were not included due to poor fit of their individual R1_ρ_ dispersion data) | | | | | | | |

**Figure S9.** Simultaneous fit of the two-site fast exchange model (eq 3, main text) to (a) spin-lock field and (b) offset dependence of ^13^C R_1ρ_ rates of the C1′ carbon of U14 of SL UCUUU in the bound state. The spin-lock dependence was measured in an on-resonance experiment while the offset dependence was measured for a spin-lock field of 214 Hz. The measured rates are symmetric with respect to positive and negative spin-lock offsets and a single maximum occurs at zero offset as expected for fast exchange. Data were measured at 313K and ^1^H frequency of 500 MHz. The solid curves correspond to a global fit with a k_ex_ value of 6967 ± 447s^-1^.

**Figure S10.** Dependence of exchange parameter, Ф_ex_ on the square of chemical shift difference between bound and free states for PTB RRM1 residues listed in table S5. A linear correlation is expected in the fast exchange limit if the observed relaxation dispersion is due to exchange between the bound and free states (see equation 3, main text).

**
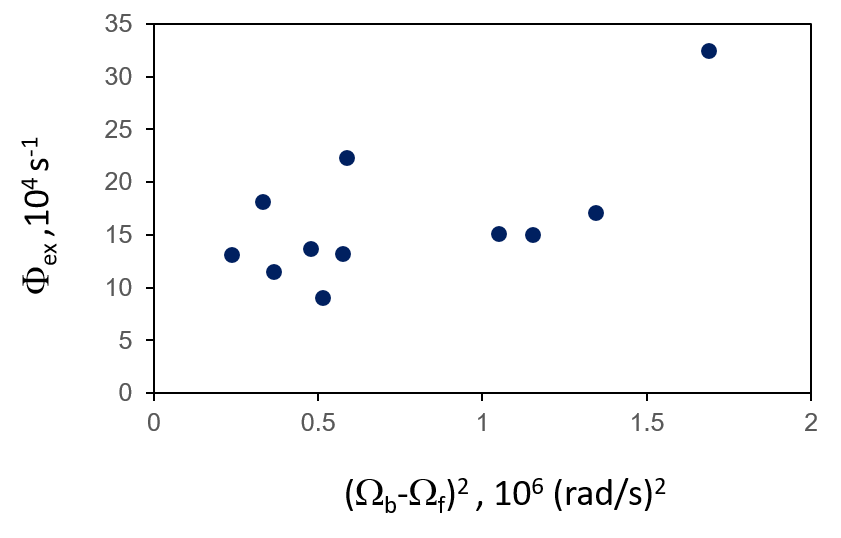
**

**Table S6**. R_ex_ estimated from RCZZ I_z_S_z_ experiment for wild type PTB RRM1 and PTB RRM1 L151G at 298K. “e” stands for error of the estimated parameter.

|  | PTB RRM1 | | PTB RRM1 L151G | |
| --- | --- | --- | --- | --- |
| Residue | R_ex_, s^-1^ | eR_ex_ | R_ex_, s^-1^ | eR_ex_ |
| K46 | 1.57 | 0.07 | 2.85 | 0.18 |
| F47 | 1.30 | 0.07 | 2.83 | 0.11 |
| G49 | 1.58 | 0.08 | 5.58 | 0.49 |
| D50 | 1.12 | 0.07 | 1.99 | 0.08 |
| A54 | 0.77 | 0.07 | 1.65 | 0.10 |
| G55 | 1.51 | 0.06 | 1.72 | 0.10 |
| V56 | 1.10 | 0.07 | 0.57 | 0.06 |
| R59 | 1.85 | 0.21 | 1.33 | 0.31 |
| V60 | 0.02 | 0.29 | 0.30 | 0.13 |
| I61 | -0.32 | 0.34 | -0.23 | 0.16 |
| H62 | 0.55 | 0.35 | 0.03 | 0.22 |
| I63 | 0.96 | 0.36 | 1.23 | 0.30 |
| R64 | 1.04 | 0.23 | 1.28 | 0.13 |
| K65 | 1.25 | 0.31 | 1.26 | 0.24 |
| I68 | 2.94 | 0.24 | 2.45 | 0.21 |
| D69 | 1.95 | 0.30 | 1.56 | 0.33 |
| V70 | 4.51 | 0.18 | 4.47 | 0.13 |
| T71 |  |  | 1.31 | 0.15 |
| E72 | 1.29 | 0.31 | 0.96 | 0.17 |
| G73 | 1.16 | 0.20 | 1.17 | 0.14 |
| E74 | 1.85 | 0.27 | 1.79 | 0.23 |
| V75 | 1.07 | 0.21 | 1.52 | 0.21 |
| I76 | 1.54 | 0.33 | 1.77 | 0.15 |
| V77 | 3.28 | 0.23 | 2.42 | 0.17 |
| L78 | 2.05 | 0.29 | 1.21 | 0.16 |
| G79 | 2.24 | 0.29 | 0.51 | 0.15 |
| L80 | 2.20 | 0.31 | 1.61 | 0.20 |
| F82 | 2.31 | 0.30 | 2.14 | 0.25 |
| G83 | 3.24 | 0.16 | 2.77 | 0.06 |
| K84 |  |  | 1.49 | 0.08 |
| V85 | 1.05 | 0.22 | 0.83 | 0.13 |
| T86 | 6.01 | 0.80 | 2.79 | 0.17 |
| N87 |  |  | 1.38 | 0.15 |
| L89 | 4.11 | 0.57 | 2.46 | 0.18 |
| M90 | 0.92 | 0.19 | 1.01 | 0.09 |
| L91 | 2.58 | 0.24 | 2.19 | 0.12 |
| K92 |  |  | 1.71 | 0.11 |
| K94 | 2.63 | 0.26 | 3.46 | 0.19 |
| Q96 | 1.22 | 0.19 | 0.94 | 0.12 |
| F98 | 2.32 | 0.26 | 1.35 | 0.15 |
| I99 | 4.87 | 0.54 | 4.29 | 0.16 |
| E100 | 1.72 | 0.47 | 1.23 | 0.34 |
| M101 | 1.37 | 0.39 | 3.27 | 0.34 |
| N102 | 2.52 | 0.25 | 1.58 | 0.16 |
| T103 | 1.06 | 0.19 | 1.04 | 0.14 |
| E104 | 0.95 | 0.31 | 1.00 | 0.16 |
| E105 | 1.55 | 0.23 | 1.33 | 0.19 |
| A106 |  |  | 0.45 | 0.12 |
| A107 |  |  | 0.26 | 0.13 |
| N108 | 0.91 | 0.20 | 0.47 | 0.10 |
| T109 | 1.05 | 0.23 | 0.88 | 0.19 |
| M110 | 0.48 | 0.25 | 0.75 | 0.17 |
| V111 | 1.25 | 0.24 | 1.68 | 0.13 |
| N112 | 1.21 | 0.18 | 0.96 | 0.17 |
| Y113 | 0.95 | 0.28 | 0.42 | 0.19 |
| T115 | 1.01 | 0.21 | 2.10 | 0.12 |
| S116 | 1.71 | 0.34 | 1.32 | 0.22 |
| V117 | 2.37 | 0.19 | 1.80 | 0.06 |
| T118 | 1.39 | 0.10 | 0.72 | 0.03 |
| V120 |  |  | 1.77 | 0.16 |
| L121 | 1.69 | 0.13 | 2.85 | 0.22 |
| R122 | 5.91 | 0.74 | 5.46 | 0.43 |
| G123 | 2.02 | 0.12 | 1.35 | 0.13 |
| Q124 | 1.78 | 0.18 | 1.45 | 0.12 |
| I126 | 5.11 | 0.39 | 4.47 | 0.19 |
| Y127 | 6.53 | 0.53 | 6.42 | 0.36 |
| I128 | 2.32 | 0.20 | 1.77 | 0.11 |
| Q129 |  |  | 0.83 | 0.21 |
| S131 | 2.15 | 0.27 | 1.29 | 0.33 |
| E135 | 11.98 | 0.79 | 9.61 | 0.28 |
| L136 | 5.11 | 0.17 | 5.16 | 0.15 |
| K137 | 5.37 | 0.13 | 2.49 | 0.17 |
| T138 | 1.70 | 0.08 | 2.27 | 0.12 |
| D139 | 1.59 | 0.10 |  |  |
| S141 | 1.28 | 0.07 | 1.96 | 0.20 |
| A145 | 1.67 | 0.07 | 1.71 | 0.10 |
| R146 | 2.02 | 0.07 | 1.39 | 0.09 |
| A147 | 1.60 | 0.08 | 1.68 | 0.15 |
| Q148 | 2.74 | 0.08 | 1.41 | 0.11 |
| A149 | 3.78 | 0.08 | 1.68 | 0.15 |
| A150 | 1.15 | 0.06 | 1.61 | 0.09 |
| L151 | 1.75 | 0.04 | 2.42 | 0.27 |
| Q152 | 2.46 | 0.05 | 1.70 | 0.10 |
| A153 | 5.29 | 0.09 | 1.34 | 0.11 |
| V154 | 6.86 | 0.05 | 0.84 | 0.08 |
| N155 | 3.34 | 0.08 | 2.79 | 0.08 |
| V157 | 0.97 | 0.06 | 1.27 | 0.10 |
| Q158 | 1.30 | 0.07 | 1.85 | 0.12 |
| S159 | 1.52 | 0.06 | 3.48 | 0.08 |
| G160 | 0.88 | 0.06 | 2.46 | 0.21 |
| N161 | 1.10 | 0.05 | 2.38 | 0.12 |
| L162 | 0.90 | 0.05 | 1.34 | 0.09 |
| A163 | 0.25 | 0.01 | 0.30 | 0.01 |

**Figure S11.** Fits of General Exchange equation to ^15^N CPMG dispersion data of PTB RRM1 obtained at two fields (900 and 750MHz; triangles and circles respectively). Dispersion data for the L151G mutant at 750MHz is shown in blue.

| **Table S7** Two site general exchange parameters from fits to CPMG dispersion data of C terminus residues^ǂ^ of PTB RRM1at 298K. | | | | | | |
| --- | --- | --- | --- | --- | --- | --- |
| Residue | R_2_^0^(750), s^-1^ | R_2_^0^(900), s^-1^ | Δω, ppm | p_b_, % | k_ex_, s^-1^ | Reduced χ^2 §^ |
| I126^*^ | 25.75 ± 0.11 | 27.87 ± 0.18 | 0.90 ± 0.86^a^ | 0.72 ± 0.12 | 672 ± 466 | 1.61 |
| E135 | 26.89 ± 0.32 | 33.07 ± 0.39 | 3.01 ± 0.43^a,d^ | 0.54 ± 0.10 | 938 ± 493 | 2.91 |
| K137 | 17.58 ± 0.11 | 24.00 ± 0.16 | 0.578^b,d^ | 2.21 ± 0.60 | 1401 ± 374 | 1.17 |
| Q148 | 13.59 ± 0.04 | 22.68 ± 0.07 | 0.970^b^ | 0.32 ± 0.05 | 795 ± 242 | 0.62 |
| A149 | 13.78 ± 0.07 | 23.05 ± 0.10 | 1.388^b^ | 0.26 ± 0.03 | 718 ± 359 | 1.87 |
| A153 | 13.70 ± 0.08 | 22.30 ± 0.11 | 1.396^b^ | 0.38 ± 0.06 | 1095 ± 307 | 1.98 |
| V154 | 13.61 ± 0.04 | 19.74 ± 0.06 | 1.492^b^ | 0.36 ± 0.02 | 683 ± 153 | 0.79 |
|  |  |  |  |  |  |  |
| Q148 | 13.56 ± 0.06 | 22.64 ± 0.09 | 1.491^c^ | 0.18 ± 0.03 | 901 ± 419 | 0.79 |
| A149 | 13.61 ± 0.18 | 22.84 ± 0.28 | 2.615^c^ | 0.14 ± 0.05 | 1530 ± 1308 | 3.00 |
| A153 | 13.36 ± 0.27 | 21.88 ± 0.43 | 3.083^c^ | 0.19 ± 0.08 | 2116 ± 1344 | 3.55 |
| A154 | 13.00 ± 0.46 | 18.93 ± 0.72 | 3.615^c^ | 0.20 ± 0.01 | 2609 ± 1927 | 3.56 |
| ^ǂ^The CPMG dispersion curves for the domain residues have a much smaller amplitude and could not be analyzed by the general exchange equation. Moreover, for some of the domain residues the larger offsets with respect to the CPMG irradiation frequency causes offset related distortions in the dispersion data preventing their analysis.  ^§^ The ratio of the χ^2^ statistic for the global fit to that for the individual fits is less than 1.5 for the four residues Q148, A149, A153 and V154. Setting the parameter Δω to the chemical shift difference between the PTB RRM1-SL UCUUU complex and the mutant, corresponding to an exchange involving a fully folded α3 helix and random coil, resulted in a poorer fit to the experimental data. Statistical comparison of the fits was based on χ^2^ and Akaike information criterion (AIC) (6).  ^*^Dispersion is weak and shifted to low ν_CP_ values. p_b_ and Δω show a correlation coefficient ~1 and do not give a unique fit.  ^a^Δω is a fit parameter.  ^b^Δω is set to the chemical shift difference between wild type PTB RRM1 and L151G PTB RRM1.  ^c^Δω is set to the chemical shift difference between wild type PTB RRM1 SL-UCUUU complex and L151G PTB RRM1.  ^d^ The dispersion data for residue E135 gives the best fit with Δω values higher than the chemical shift difference between wild type and mutant. For Lys137 on the other hand, treating Δω as a free parameter does not give a significantly better fit compared to the fit obtained when Δω is fixed to the chemical shift difference between the wild type and mutant forms. | | | | | | |

**Figure S12.** ^13^C-CPMG relaxation-dispersion data of sidechain methyl groups in PTB RRM1 at 900MHz and temperature 298K. Methyl groups of Ile and Leu, and Val residues were selectively labelled with ^13^C by using ^13^C_2_- and ^13^C_1_-glucose in the growth media as carbon source respectively (7). Otherwise, samples were prepared as described for uniformly ^13^C-labeled samples. Data from residues in the domain and C-terminal segment are shown in green and red respectively. Sidechain ^13^C relaxation dispersion indicative of slow dynamics is mostly observed in residues (or neighboring residues) which also showed backbone ^15^N relaxation dispersion. Of these I76, V85, L89, V154 are at the RRM domain-α3 interface.

**
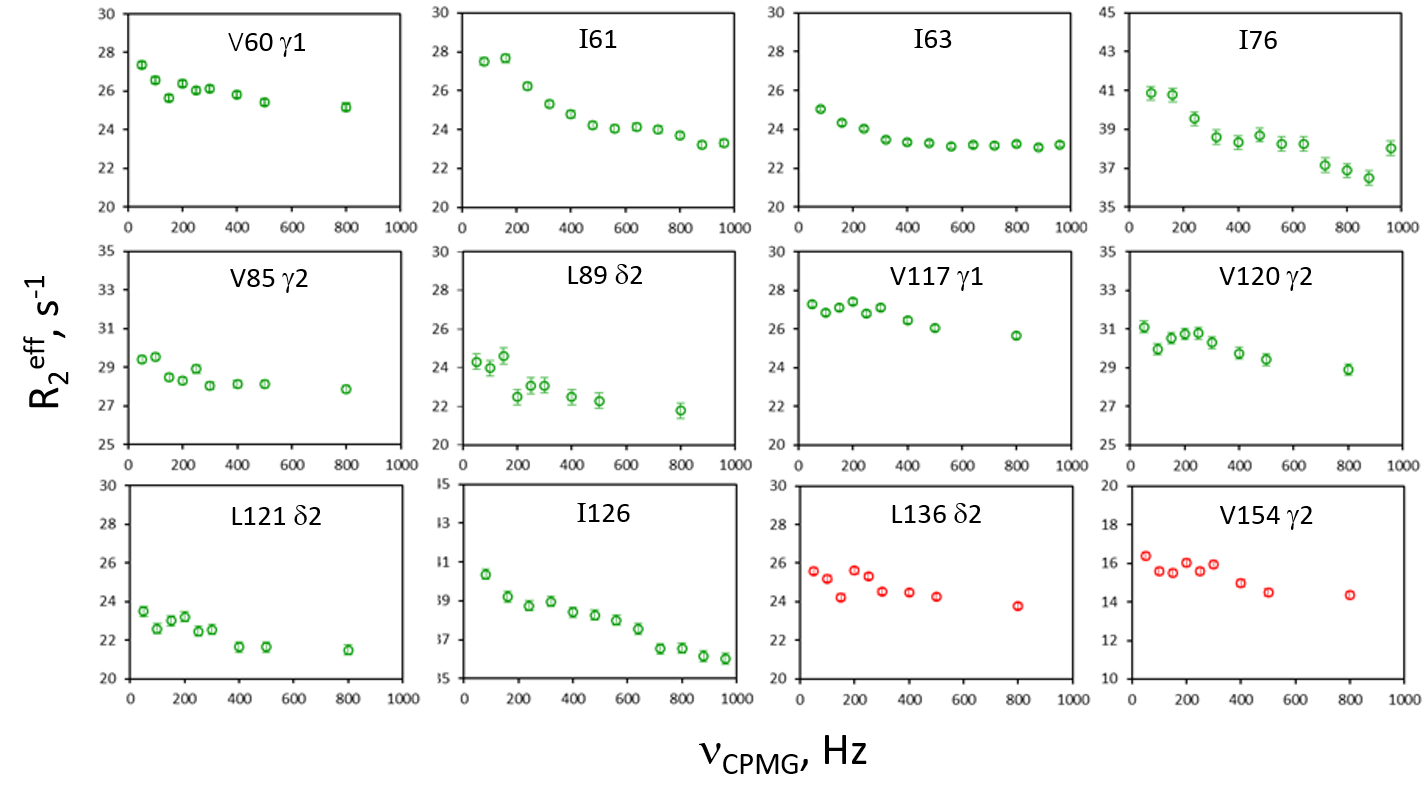
**

**Figure S13.** ^13^C-resolved NOESY HA/CA strips of free PTB RRM1 at 298K with positions of medium range HA-HN NOEs supporting a helical conformation in the C-terminal residues Q144−V154**.** Intraresidue HA-HN NOEs are indicated by a solid circle, and expected positions of medium range HA-HN NOEs are indicated by crosses and the residue of the HN atom.

**
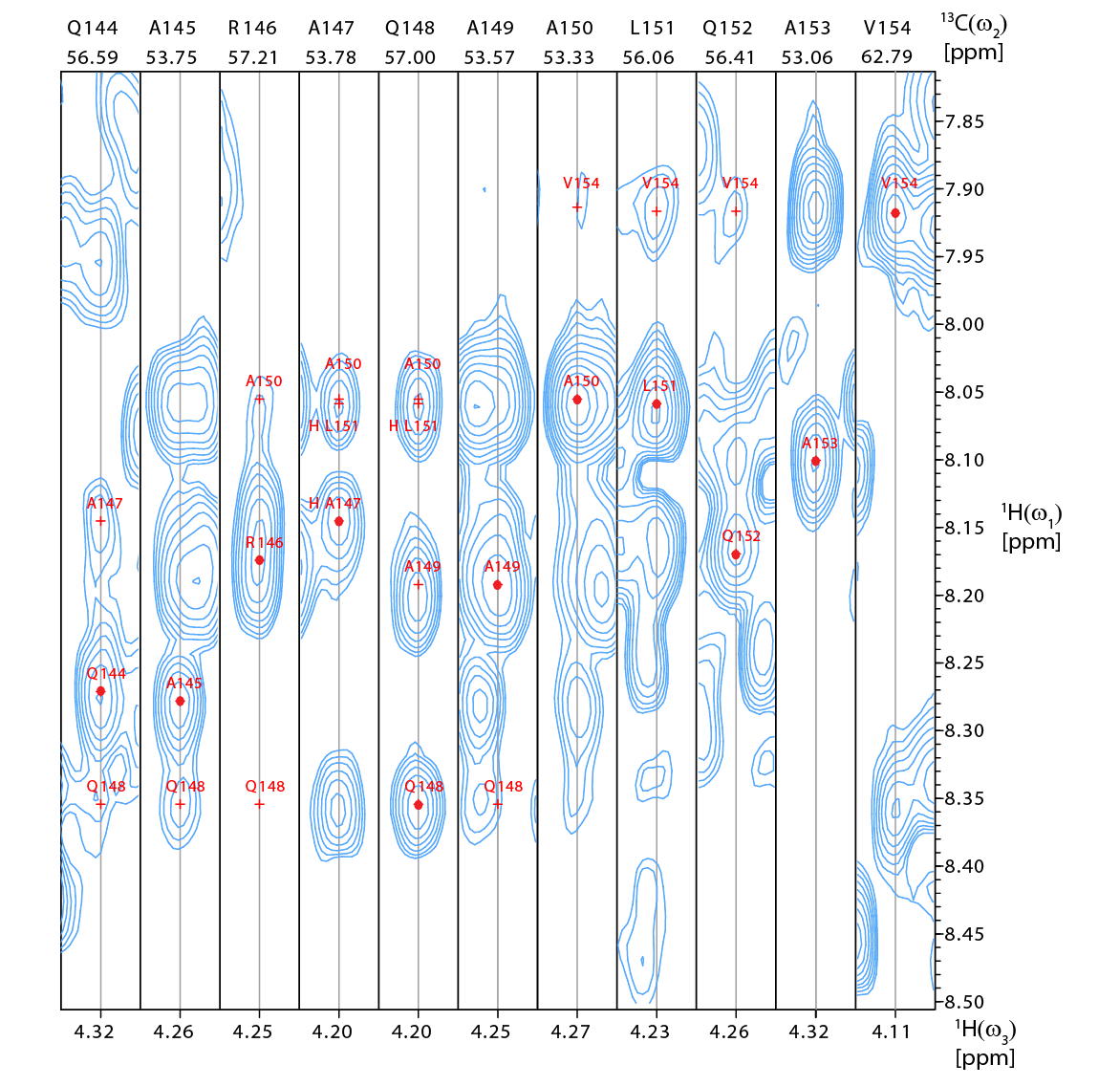
**

**Figure S14.** ^1^H,^13^C strips of residues in the C-terminus of free PTB RRM1 from the ^13^C-resolved NOESY at 298K showing NOEs supporting RRM-α3 contacts that were also observed in the complex. Red crosses show expected positions and assignments of NOEs corresponding to long-range RRM-α3 contacts found in the complex. Most of these contacts are observed suggesting the dynamic helix in the free PTB RRM1 makes similar contacts to the domain as those seen in the complex.


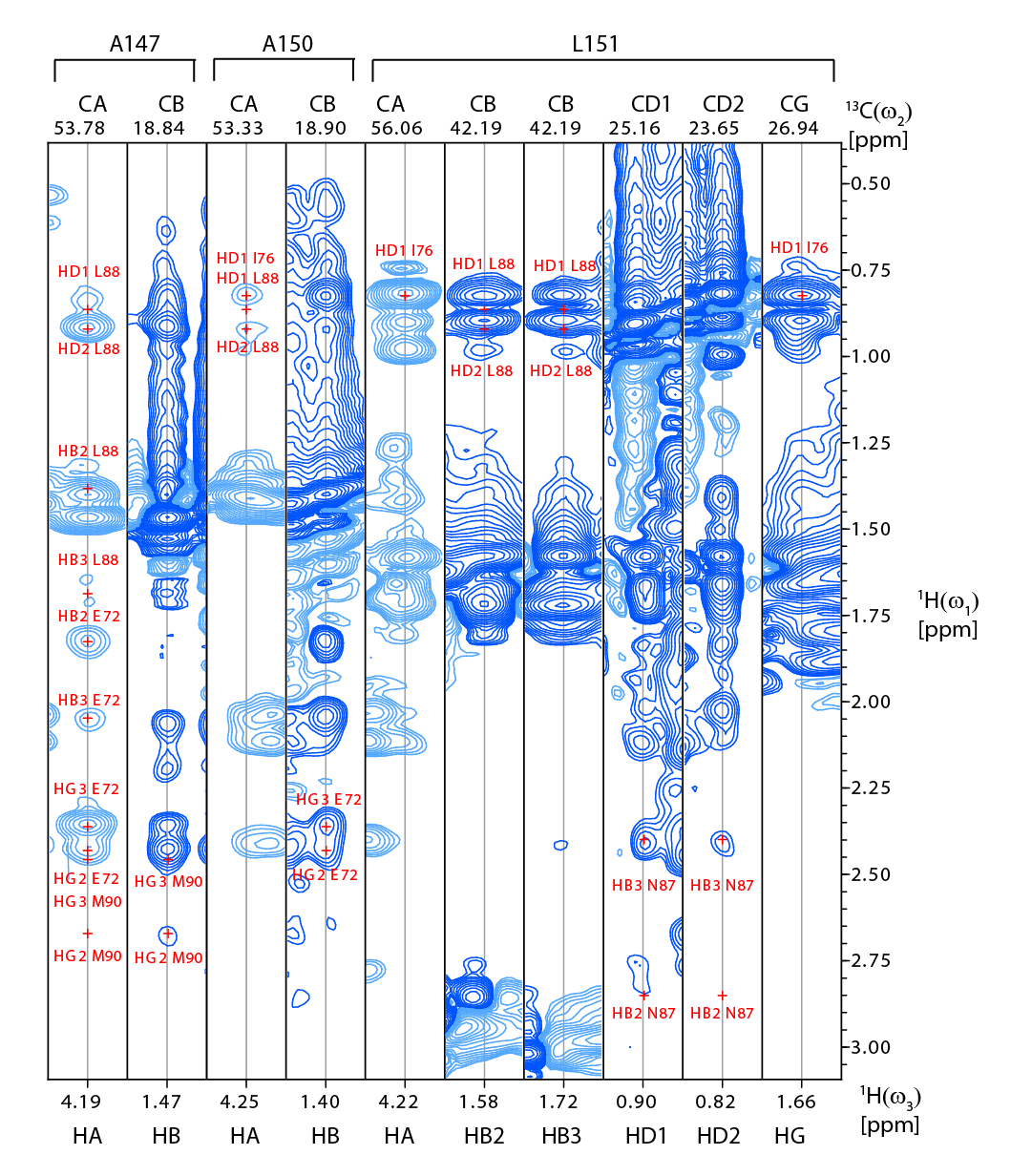


**Figure S15.** ^1^H,^13^C-strips of residues in the RRM domain of free PTB RRM1 from the ^13^C-resolved NOESY at 298K. Positions marked with crosses indicate the predicted location of NOEs connecting residues in the RRM domain to the C-terminus based on RRM-α3 contacts observed in the complex.


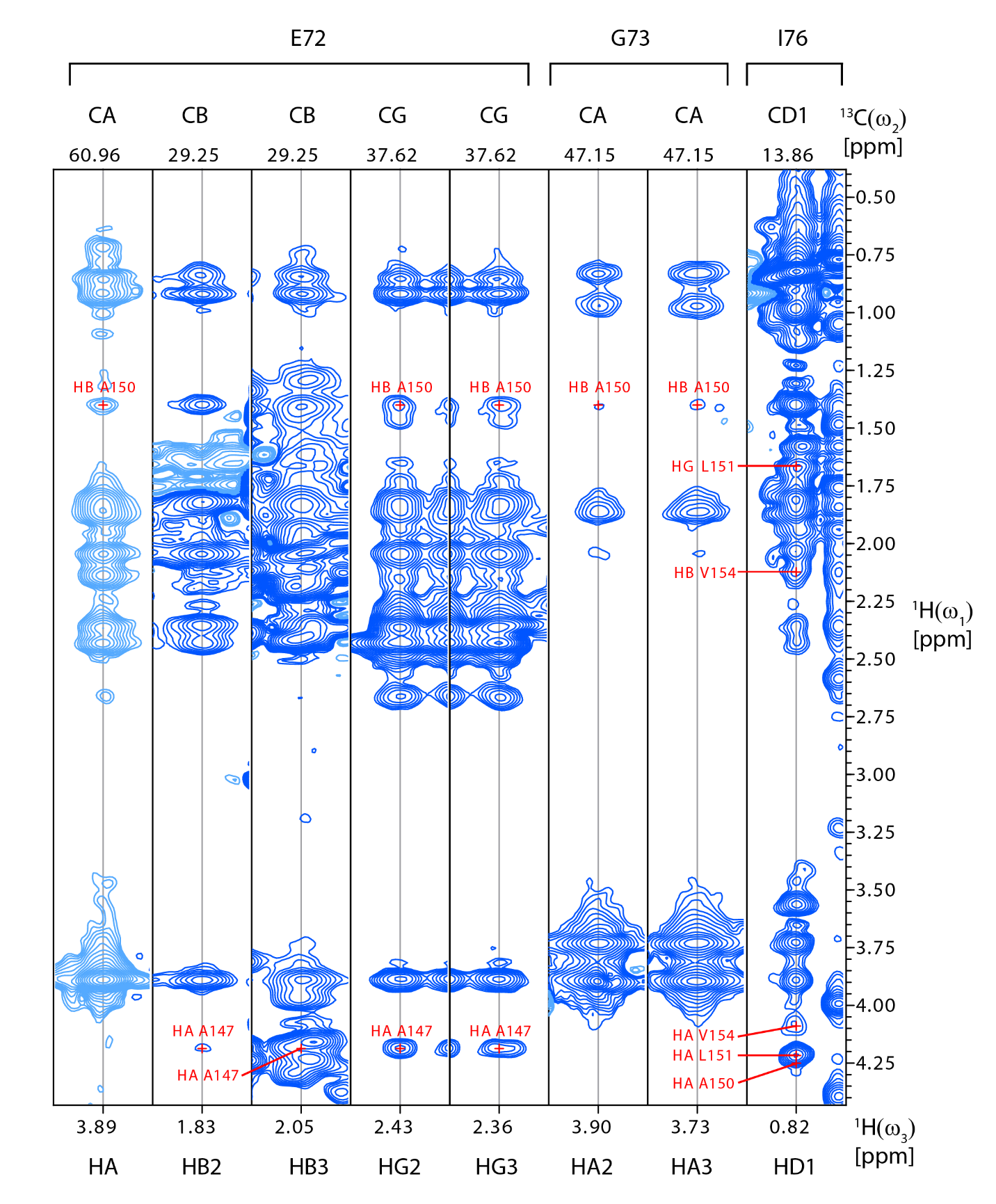


**Figure S16.** ^15^N-resolved NOESY HN,N-strips of free PTB RRM1 at 298K with positions marked corresponding to NOEs connecting RRM domain to α3 in the complex.


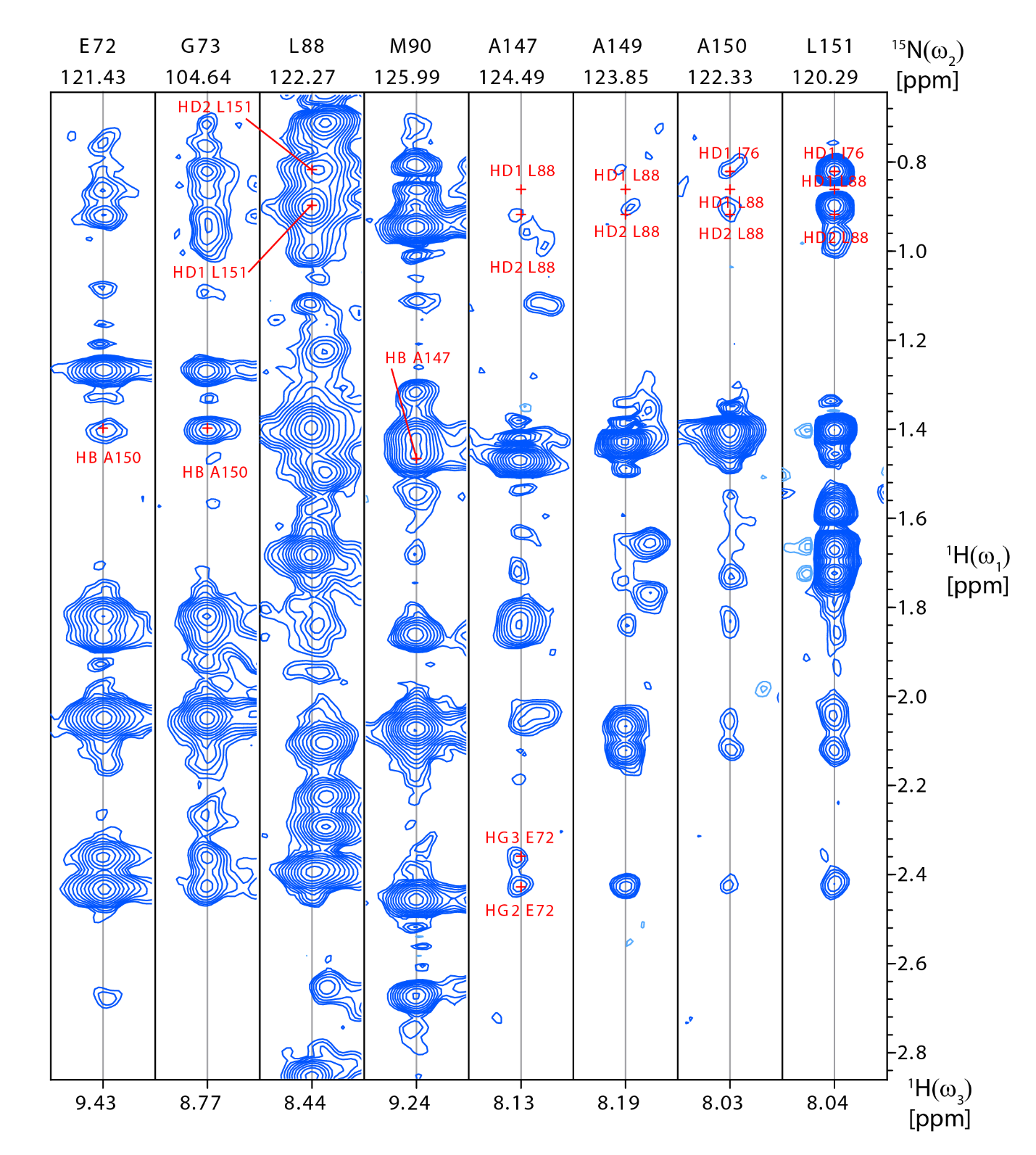


| **Table S8**. NMR structure statistics for PTB RRM1 at 298K. | | | |
| --- | --- | --- | --- |
| **NMR restraints** | Ambiguous restraint ^a^ | Assignments in class ^b^ | All assignments ^c^ |
| Distance Restraints | 3670 | 5555 | 5555 |
| intraresidual | 609 | 866 | 962 |
| sequential (\|i-j\|=1) | 976 | 1284 | 1647 |
| medium range (1<\|i-j\|<5) | 831 | 1096 | 1457 |
| long range (\|i-j\|>=5) | 1683 | 2309 | 2780 |
| long range (RRM to α3) ^d^ | 111 (22^e^) | 164 | 354 |
| Torsion angles |  | 0 |  |
| **Structure statistics** |  |  |  |
| Distance constraint violations  > 0.1-0.2 Å  Maximal violation Å  Target function (Å^2^) |  | 3 ± 2  0.14 ± 0.02  1.82 ± 0.05 |  |
| **Ramachandran plot statistics** |  |  |  |
| Residues in most favored regions (%)  Residues in additionally allowed regions (%)  Residues in generously allowed regions (%)  Residues in disallowed regions (%) |  | 64.7 ± 2.3  32.4 ± 2.5  2.6 ± 0.9  0.3 ± 0.5 |  |
| **RMSD to mean structure statistics** |  |  |  |
| RRM + α3 (58−155)  Backbone atoms  Heavy atoms  RRM domain only (58−129)  Backbone atoms  Heavy atoms |  | 0.51 ± 0.10  0.81 ± 0.08  0.21 ± 0.06  0.50 ± 0.06 |  |
| ^a^ Ambiguous restraints contain multiple assignments. A restraint including an assignment in a given class is included in the total and may be counted again in another class.  ^b^ Number of assignments in the class associated with ambiguous restraints (e.g., intraresidual). The sum total of all classes equals the total number of assignments (5555).  ^c^ Total of all assignments associated with the ambiguous restraints in a given class.  ^d^ Restraints connecting residues 60−129 (RRM) to 144−155 (α3).  ^e^ Number of constraints which were unambiguously assigned between RRM domain and α3. | | | |

**Figure S17.** Comparison of unambiguous RRM-α3 constraints identified in structures of PTB RRM1 in the free state and bound to SL UCUUU RNA. Upper distance limits derived from NOEs are shown as magenta lines connecting positions of atoms in the RRM and the C-terminal α3 region in a representative member of the ensemble of structures calculated for (A) the free PTB RRM1 and (B) PTB RRM1 in the complex with UCUUU stemloop. Only restraints derived from NOEs which were assigned exclusively as long-range contacts between atoms in the RRM domain and the α3 helix are shown. Restraints which were ambiguously assigned to either a long-range RRM-α3 distance or a distance connecting atoms within one of these two regions in the calculation of free PTB RRM1 were excluded in A. No ambiguous restraints are present in the final ensemble of structures for the PTB RRM1-SL UCUUU complex, which was calculated using the standard CYANA protocol


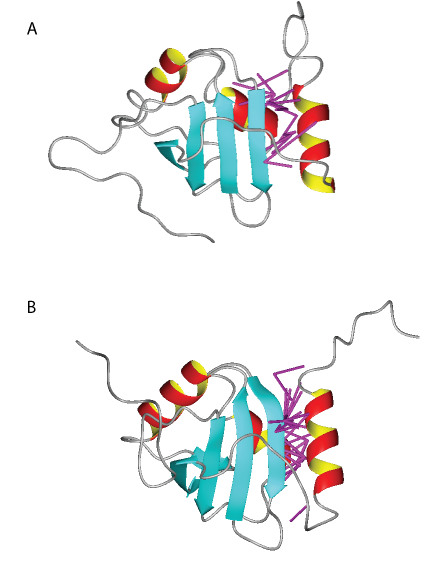


**Figure S18.** Comparison of the backbone coordinates of the ensemble of free and SL UCUUU-bound states of PTB RRM1. (A) Superposition for best fit of free (magenta) and bound structure (blue), shown in stick representation, onto the average coordinates of backbone atoms from residues 60−129 and 144−154 of the SL UCUUU-bound state of PTB. RMSD to the average coordinates for these residues are 0.45Å and 0.33Å for free and bound ensembles respectively. (B) and (C) Alignments of the ensemble of free and bound state ensembles shown in ribbon representation respectively onto the same average coordinates.


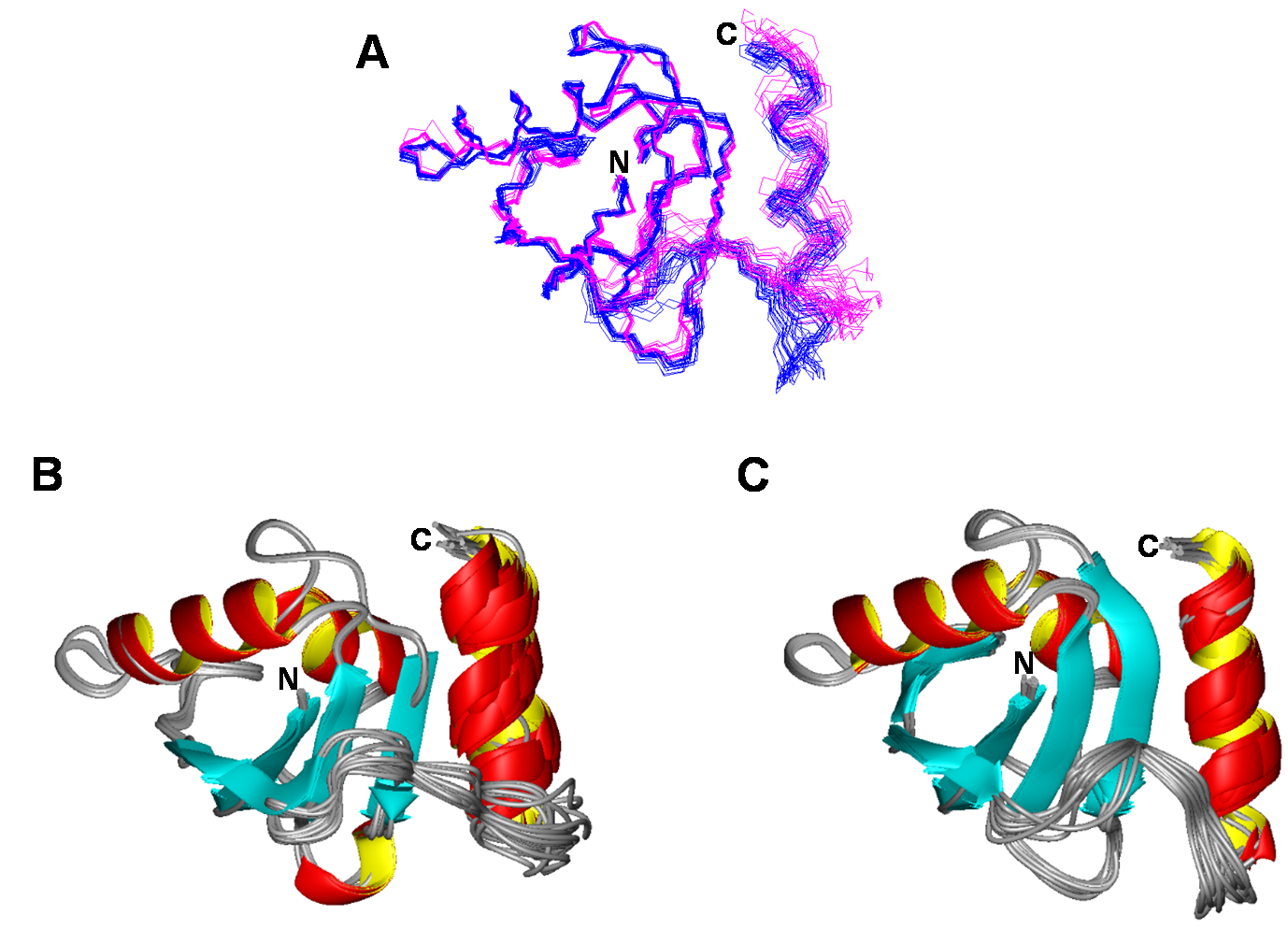


| **Table S9.** Summary of MD simulations | | |
| --- | --- | --- |
| **Standard MD simulation** | | |
| **system** | **number × length [μs]** | **short summary** |
| **free protein starting from complex structure^a^** | | |
| free_protein | 1 × 10 | α3 remains folded. |
| linker removed^b^ | 1 × 10 | enhanced dynamics in α3, rotation of α3 relative to domain, i.e., unstable binding register of α3. The secondary structure of the helix remained stable. |
| L151G | 1 × 2 | L151G mutation destabilizes the binding of α3 to the domain followed by loss of helical structure in part of the helix proximal to the G151 residue |
| **free protein starting from free conformers^c^** | | |
| conformer1 | 2 × 2 | α3 remains folded. |
| conformer3 | 1 × 20  1 × 2 | Partially folded α3 folds in run #1, unfolds in the run #2. In run #1, α3 initially shows alternative docking to domain. At the end of the simulation, it takes on the native binding pattern observed in the complex structure. |
| conformer10 | 2 × 2 | α3 remains folded. |
| **free RNA^d^** | | |
| free RNA | 5 × 2 | U10−U14 bp is lost in one of five simulations and can be transiently disrupted in the others. The C11, U12, U13 nucleotides sample many different conformations. |
| **complex (protein + SL UCUUU)^e^** | | |
| complex | 3 × 10 | α3 remains folded. Reversible changes in its binding register observed in response to temporary disruption of U13 binding to the protein. The U10−U14 base pair can be transiently disrupted and U10 makes new contacts to the protein while U14 contacts U13. |
| complex with  U10−U14 base pair stabilized by restraints | 1 × 2 | α3 remains folded. No changes in protein-RNA H-bond network except H-bonds facilitated directly by U10 and U14 bases. |
| helix α3 removed^b^ | 1 × 1 | Greatly increased fluctuations of the β4−α3 linker. Interactions with U12 and U13 gradually disrupted. |
| complex L151G | 1 × 5 | L151G mutation destabilizes α3 by introducing kink from which the helix unwinds |
| **complex (protein + SL UCGUU)** | | |
| complex | 1 × 10 | α3 is stable similar to the SL UCUUU complex. Altered dynamics of the protein-RNA interface residues is observed due weakened interactions between U13 and the protein. |
| **REST2 simulations on α3 region^f^** | |  |
| **system** | **replicas × length [μs]** | **short summary of conclusions** |
| **free protein** | | |
| free_protein^a^ | 6 × 10 | α3 unfolds in five out of six simulations. |
| delete β4−α3 linker | 6 × 2 | α3 loses orientation to domain, sometimes unfolds. |
|  |  |  |
| **complexes** |  |  |
| complex SL UCUUU | 6 × 10 | α3 unfolds in one out of six simulations.  α3 stabilized via waters bridging contacts to the binding pocket of U13 formed by β4−α3 linker. Loss of these interactions leads to observed conformational changes in α3. During restoration of these contacts, similar interactions with U12 can also temporarily serve to facilitate the necessary stabilization. |
| complex SL UCGUU | 6 × 10 | α3 unfolds in two out of six simulations  α3 somewhat less stable than with U12 because G12 is able to form intramolecular interactions with U13 which can compete with U13 interactions with its binding pocket |
|  |  |  |
| ^a^ Starting coordinates obtained by removing RNA from the complex structure.  ^b^ β4-α3 linker and α3 refer to residues 133−139 and 143−155, respectively.  ^c^ Simulations of the free protein based on conformers 1, 3 and 10 of the combined ensemble of the free protein obtained from 20 independent structure calculations.  d Starting coordinates obtained by removing protein from the complex structure.  ^e^ See supplementary note S2 below.  ^f^ Please see Kuhrova *et al.* 2019, for a discussion of technical details of the REST2 calculations (8). | | |

**Note S2. Behavior of the U10**−**U14 base pair in MD simulations.**

In simulations of the complex, we observed a reversible opening of the U10-U14 base pair which however was not associated with any changes in the interactions between the RNA nucleotides of the apical loop and the protein. The U10 nucleotide would subsequently form additional interactions with the protein (Figure 6A, B). We also observed loss of this base pair in one out of five simulations of the free RNA, but it was only transiently disrupted in the others. To further show that the instability of the U10−U14 base pair does not compromise the rest of the protein-RNA interface in simulations of the complex, we performed a simulation of the complex in which we stabilized the base pair by restraints. In this simulation, we observed an identical network of protein/RNA interactions formed by the RNA nucleotides of the apical loop, suggesting that binding to the RRM is not compromised.

**Table S10.** Agreement of MD trajectories with NOE Upper distance limits of NMR structure.^a,b^

| Condition | Simulation | Protein | RNA | Protein/RNA |
| --- | --- | --- | --- | --- |
| complex | all^c^  (30 μs) | 90% | 79% | 74% |
| complex REST2 | reference replica  (10 μs) | 92% | 78% | 85% |
| free_protein  started from complex structure | all  (10 μs) | 88% | - | - |
| free_protein REST2  started from complex structure | reference replica  (10 μs) | 91% | - | - |
| free_protein  started from free protein structure  conformer 1 | all (4 μs) | 88% | - | - |
| free_protein  started from free protein structure  conformer 3 | simulation 1  (2 μs) | 86% | - | - |
| free_protein  started from free protein structure  conformer 3 | simulation 2  (20 μs) | 86% | - | - |
| free_protein  started from free protein structure  conformer 10 | all (4 μs) | 86% | - | - |
| ^a^ Percentage of NOE constraints satisfied within 0.3 Å of the upper distance limit.  ^b^ Distances in MD trajectories were calculated with <1/r^6^>^-6^, where the brackets indicate an average over the entire MD trajectory, and r is the distance in a given snapshot.  ^c^ all indicates that agreement to upper distance limits was calculated for all simulations of a given condition. | | | | |

**Figure S19.** Per-residue RMSF to mean coordinates of selected 10μs-long standard MD simulations of PTB RRM1-SL UCUUU and free PTB RRM1. All-heavy atom RMSF was calculated after aligning to residues 58−155.


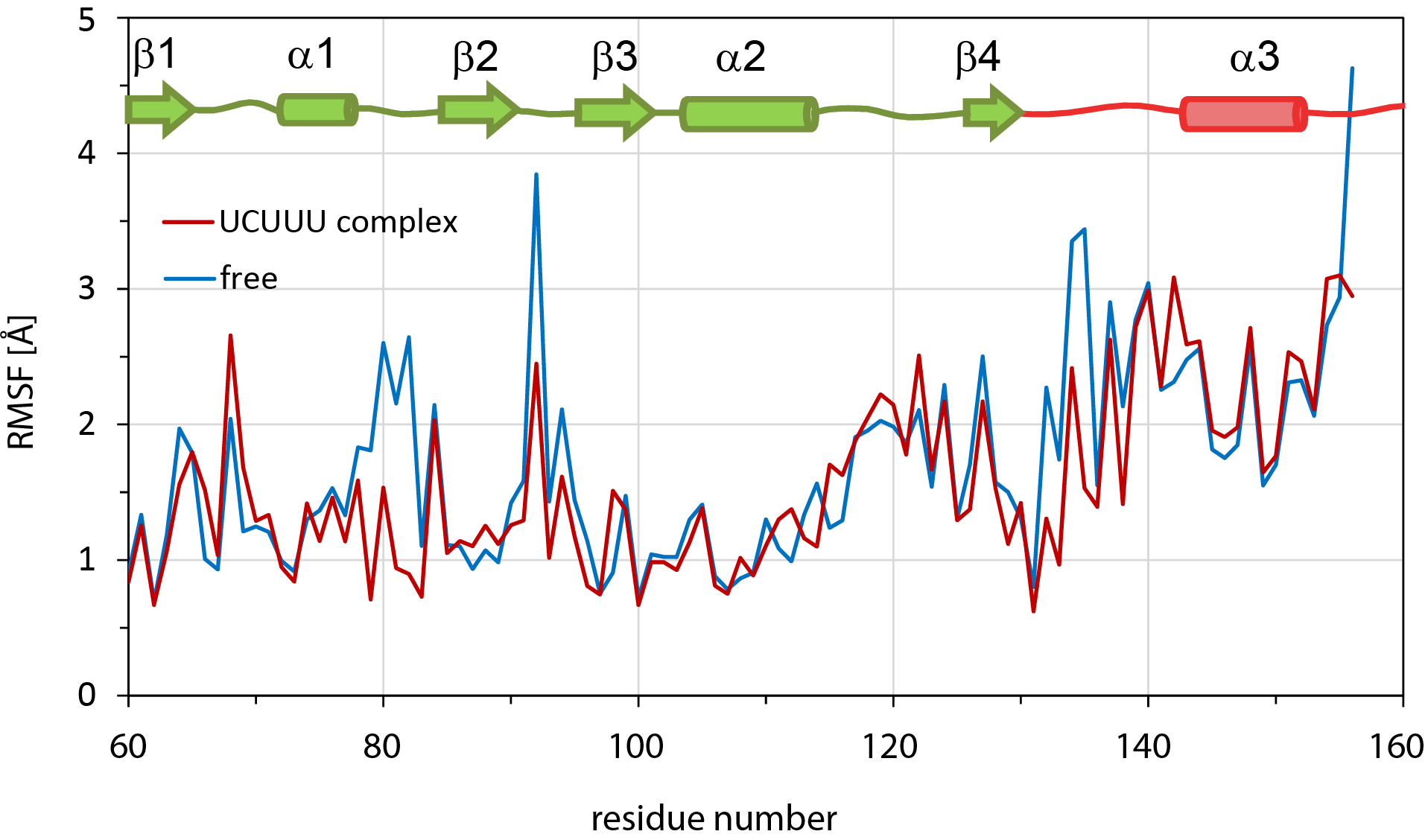


**Figure S20.** Standard MD runs starting from conformers 1,3, and 10 of the free PTB RRM1 combined ensemble obtained from three independent structure calculations shows different converged structures in the left column. The α3 helix was packed against the RRM domain like in the PTB RRM1/SL UCUUU complex at the end of both 2 μs MD runs starting from conformers 1 and 10. In contrast, MD simulations starting from conformer 3 showed an unfolded C-terminus after 2 μs in MD run #1 and an α3 helix with an alternative docking arrangement to that observed in the complex at 2 μs in the second run. We extended this simulation to 20 μs. The alternative arrangement was stable for 6 us and then gradually changed into the native one at the end of the run.


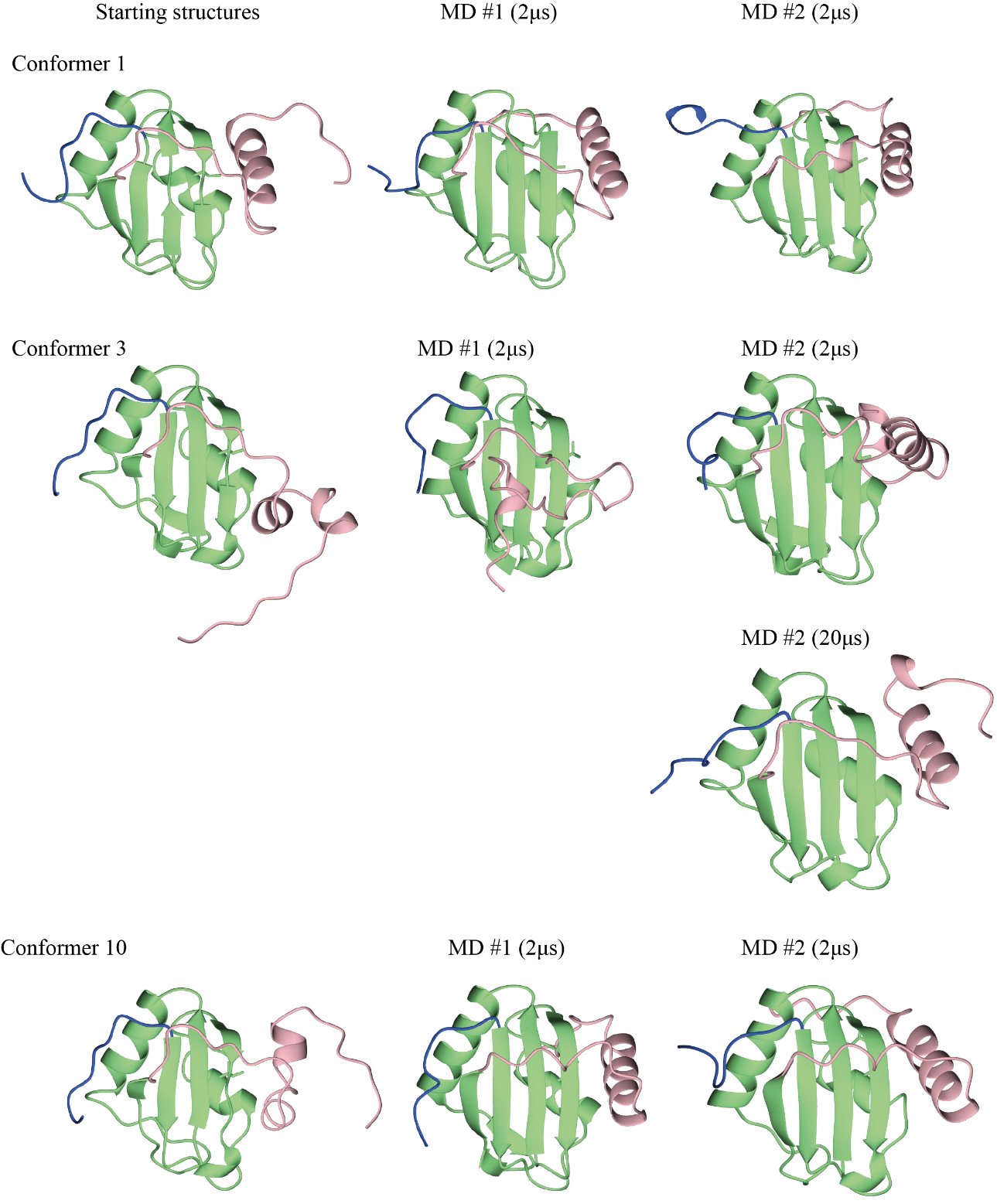


**Figure S21.** Secondary structure of α3 region in the six demultiplexed replicas (continuous trajectories traveling through the replica ladder) of the REST2 calculations for PTB RRM1 bound to SL UCUUU, PTB RRM1 bound to SL UCGUU, and free PTB RRM1.

**
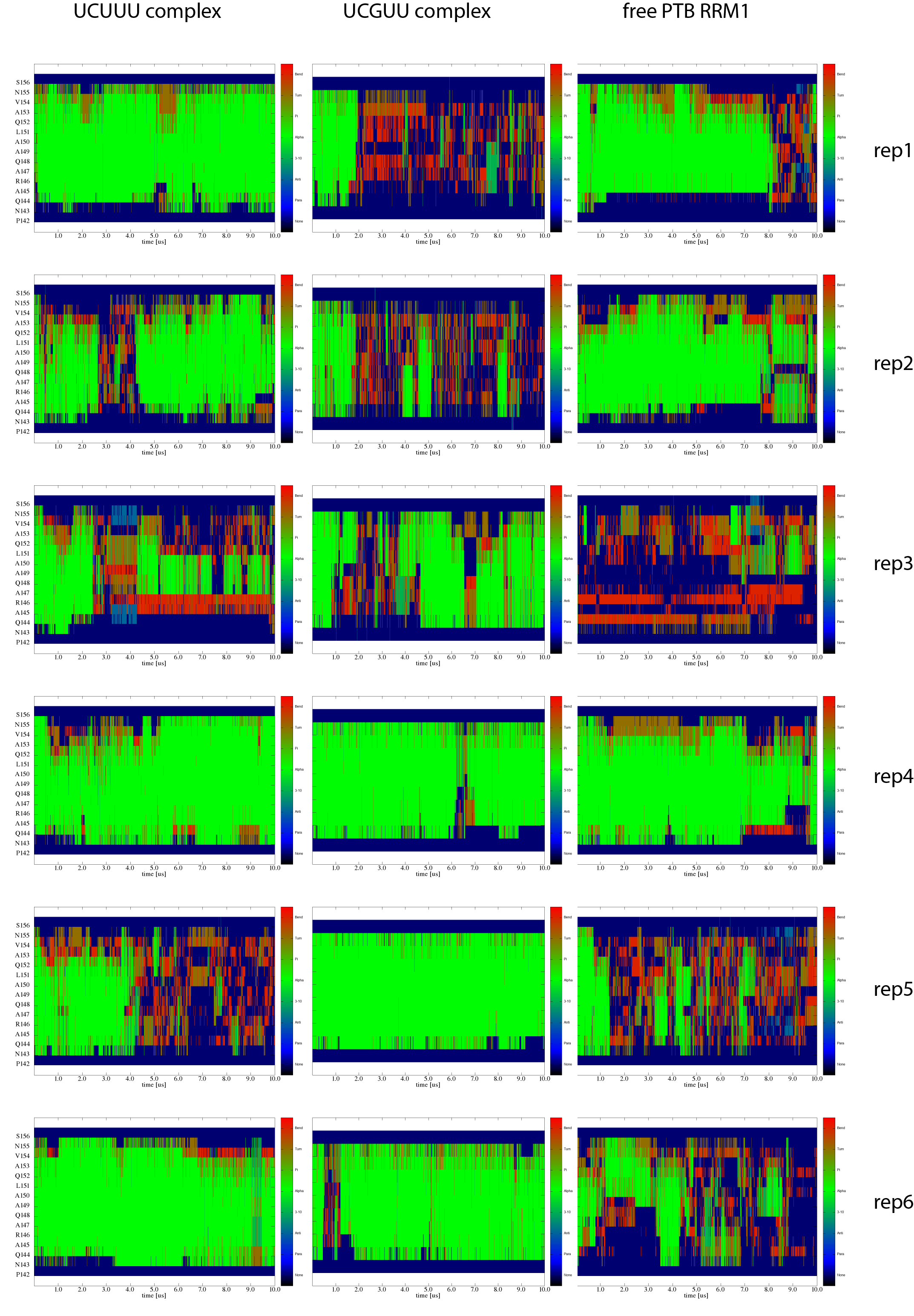
**

**Figure S22.** Average order parameters for residues of α3 (143−155) for REST2 trajectories from the reference replica (the discontinuous replica with λ = 1 which corresponds to the unbiased state) for PTB RRM1 bound to SL UCUUU, PTB RRM1 bound to SL UCGUU, and free PTB RRM1. Order parameters are calculated over a 10 ns moving window as described previously (9).
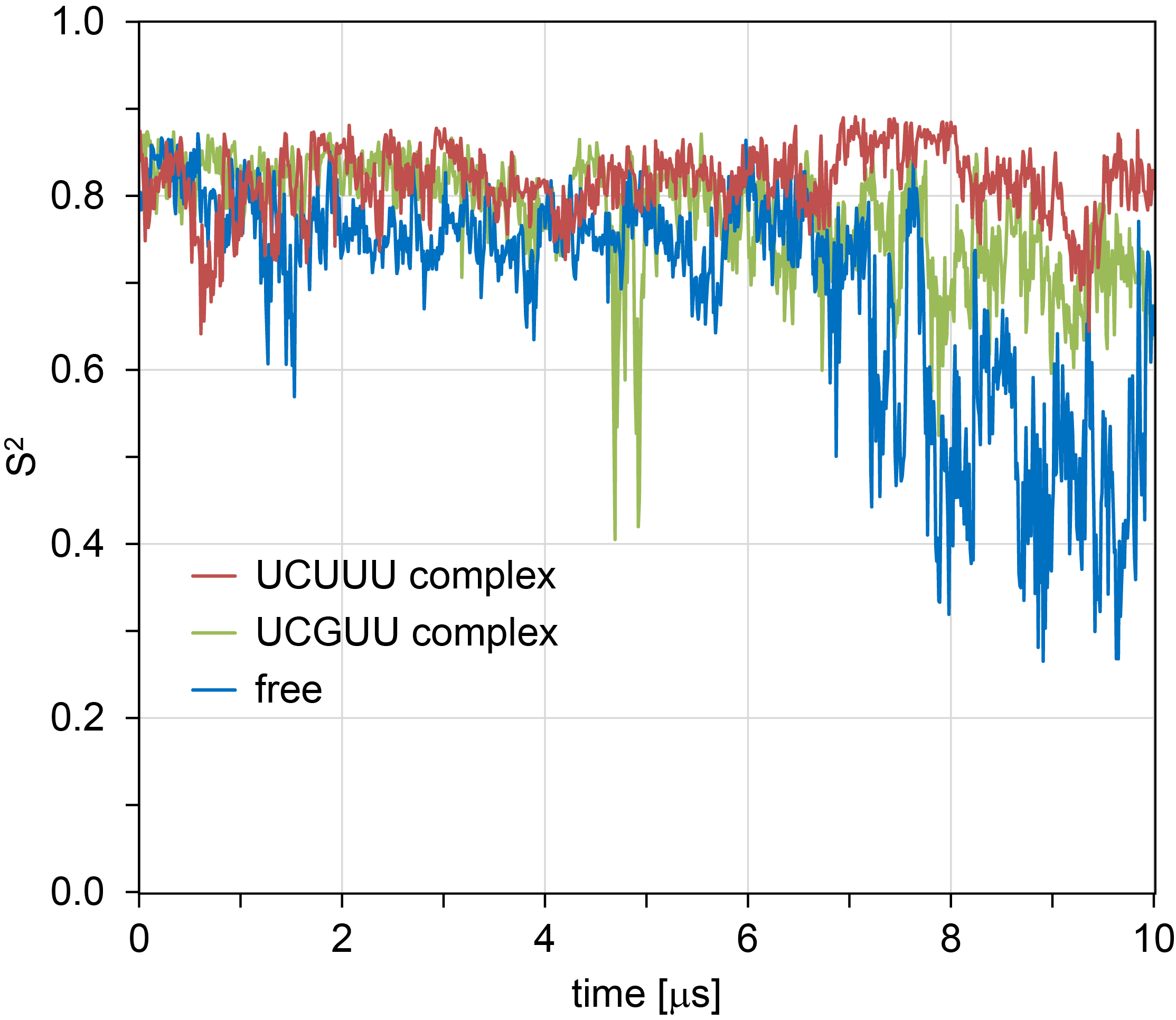


**REFERENCES**

1. Atkinson, R.A. and Kieffer, B. (2004) The role of protein motions in molecular recognition: insights from heteronuclear NMR relaxation measurements. *Prog. Nucl. Magn. Reson. Spectrosc.*, **44**, 141-187.

2. Farrow, N.A., Zhang, O.W., Szabo, A., Torchia, D.A. and Kay, L.E. (1995) Spectral density function mapping using N-15 relaxation data exclusively. *J. Biomol. NMR*, **6**, 153-162.

3. Mandel, A.M., Akke, M. and Palmer, A.G. (1995) Backbone Dynamics of *Escherichia coli* ribonuclease HI: correlations with structure and function in an active enzyme. *J. Mol. Biol.*, **246**, 144-163.

4. Wang, C.Y., Grey, M.J. and Palmer, A.G. (2001) CPMG sequences with enhanced sensitivity to chemical exchange. *J. Biomol. NMR*, **21**, 361-366.

5. Camilloni, C., De Simone, A., Vranken, W.F. and Vendruscolo, M. (2012) Determination of secondary structure populations in disordered states of proteins using nuclear magnetic resonance chemical shifts. *Biochemistry*, **51**, 2224-2231.

6. d'Auvergne, E.J. and Gooley, P.R. (2003) The use of model selection in the model-free analysis of protein dynamics. *J. Biomol. NMR*, **25**, 25-39.

7. Lundstrom, P., Teilum, K., Carstensen, T., Bezsonova, I., Wiesner, S., Hansen, D.F., Religa, T.L., Akke, M. and Kay, L.E. (2007) Fractional 13C enrichment of isolated carbons using [1-13C]- or [2- 13C]-glucose facilitates the accurate measurement of dynamics at backbone Calpha and side-chain methyl positions in proteins. *J. Biomol. NMR*, **38**, 199-212.

8. Kuhrova, P., Mlynsky, V., Zgarbova, M., Krepl, M., Bussi, G., Best, R.B., Otyepka, M., Sponer, J. and Banas, P. (2019) Improving the Performance of the Amber RNA Force Field by Tuning the Hydrogen-Bonding Interactions. *J Chem Theory Comput*, **15**, 3288-3305.

9. Showalter, S.A. and Bruschweiler, R. (2007) Validation of molecular dynamics simulations of biomolecules using NMR spin relaxation as benchmarks: Application to the AMBER99SB force field. *J. Chem. Theory Comput.*, **3**, 961-975.
